# Long-term follow-up demonstrates the curative potential of dual CD19/CD22 CAR-T-cell therapy alone or combined with autologous stem cell transplantation in TP53-altered relapsed/refractory B-cell non-Hodgkin lymphoma

**DOI:** 10.1038/s41392-025-02571-7

**Published:** 2026-02-13

**Authors:** Zekai Mao, Juan Peng, Yang Cao, Na Wang, Jue Wang, Yang Yang, Jinghuan Xu, Fankai Meng, Liting Chen, Xia Mao, Jiaqi Guo, Xiaoxi Zhou, Yicheng Zhang, Jia Wei

**Affiliations:** 1 Department of Hematology, Tongji Hospital, Tongji Medical College, Huazhong University of Science and Technology, Wuhan, Hubei China; 2 Immunotherapy Research Center for Hematologic Diseases of Hubei Province, Wuhan, Hubei China; 3 Research Institute of Huazhong University of Science and Technology in Shenzhen, Shenzhen, China; 4 Department of Hematology, Shanxi Bethune Hospital, Shanxi Academy of Medical Sciences, Tongji Shanxi Hospital, Third Hospital of Shanxi Medical University, Taiyuan, Shanxi China

**Keywords:** Haematological cancer, Cancer therapy

## Abstract

The prognostic implications of TP53 alterations in patients with relapsed or refractory (r/r) aggressive B-cell non-Hodgkin lymphoma (B-NHL) treated with chimeric antigen receptor (CAR) T-cell therapy remain inadequately characterized, particularly with respect to long-term outcomes. We report extended follow-up (median: 77.77 months) of 122 patients with r/r B-NHL who received either dual-targeted CD19/CD22 CAR-T-cell therapy alone (cohort A, *n* = 65) or following sequential autologous stem cell transplantation (ASCT; cohort B, *n* = 57). TP53 alterations were identified in 59 patients (48.4%). Within both cohorts, overall survival (OS) and progression-free survival (PFS) did not significantly differ between the TP53-altered subgroup and the wild-type subgroup (*P* >0.05). Notably, compared with CAR-T-cell monotherapy, the sequential ASCT-CAR-T-cell approach (cohort B) was associated with improved 5-year OS (70.2% vs. 40.0%) and PFS (64.9% vs. 35.4%). The 5-year cumulative incidence of nonrelapse mortality was 10.7% overall (9.2% in cohort A vs. 12.3% in cohort B). Secondary malignancies occurred in 2.5% of patients, whereas serious infection-related events beyond 3 months post-infusion were observed in 13.6%, supporting a favorable long-term safety profile. Multivariate analysis identified treatment options and the presence of bulky disease as independent adverse prognostic factors for OS and PFS. These findings suggest that dual-target CD19/CD22 CAR-T-cell therapy, particularly when integrated with ASCT, may mitigate the adverse prognostic influence of TP53 alterations, offering sustained clinical benefit with manageable long-term toxicity in r/r aggressive B-NHL.

## Introduction

The TP53 gene encodes a critical tumor suppressor protein responsible for preserving genomic stability through the modulation of cellular responses to genotoxic stress, including DNA damage repair, cell cycle arrest, apoptosis, metabolic regulation, senescence, and autophagy.^1^ Somatic alterations in TP53 are frequently identified in cases of relapsed or refractory (r/r) aggressive B-cell non-Hodgkin lymphoma (B-NHL), particularly in diffuse large B-cell lymphoma (DLBCL), where mutation rates range from ~30 to 63%.^2,3^ These genetic aberrations are well established as high-risk molecular biomarkers and are consistently associated with resistance to standard cytotoxic regimens, an aggressive clinical course, and significantly inferior overall survival (OS) and progression-free survival (PFS) compared with their wild-type TP53 counterparts.^2,4^ Despite intensive therapeutic modalities, including high-dose chemotherapy (HDT) followed by autologous stem cell transplantation (ASCT), long-term outcomes remain unsatisfactory in this high-risk group.^5–7^ For example, in the DLCL04 phase III trial evaluating HDT/ASCT as a first-line consolidation strategy for high-risk DLBCL, 5-year OS and failure-free survival (FFS) rates were substantially lower among patients with TP53 mutations (33% and 19%, respectively) than among those with wild-type TP53 (81% and 73%).^8^ Similarly, conventional targeted therapies, including lenalidomide and Bruton’s tyrosine kinase (BTK) inhibitors, have demonstrated only modest efficacy in TP53-mutant DLBCL.^9,10^ Recent investigations have focused on novel treatment approaches, such as the combination of selinexor with salvage chemotherapy, in patients with refractory DLBCL harboring TP53 alterations. In a recent clinical study, this regimen yielded an overall remission rate (ORR) of 60.4%, including a complete remission (CR) rate of 14.6%.^11^ Nonetheless, its long-term therapeutic impact remains to be determined and warrants further evaluation.

CD19-directed chimeric antigen receptor T-cell (CAR-T) therapy has become an integral second-line modality for r/r aggressive B-NHL, offering sustained remission and the potential for long-term disease control.^12^ However, its therapeutic efficacy in patients harboring TP53 mutations remains a subject of debate.^5,13^ A retrospective analysis of relapsed or refractory (r/r) DLBCL patients indicated improved survival outcomes among those with TP53 mutations treated with CD19-targeted CAR-T cells compared with nonrecipients.^14^ Conversely, other studies have reported limited benefit in this population,^13^ potentially attributable to diminished CAR-T-cell expansion, reduced persistence, or impaired antigen recognition mechanisms compared with patients receiving first-line treatment, such as in Zuma-12.^15–17^ Moreover, the adverse prognostic impact of TP53 alterations appears to persist across treatment lines, although it may vary depending on the disease phase and the accumulation of additional genetic abnormalities.^18^ Notably, downregulation of the CD19 antigen on malignant B cells occurs more frequently in TP53-altered cases, further challenging the efficacy of monospecific CAR-T-cell therapies.^19,20^ One study revealed no significant difference in OS between TP53-mutant and wild-type cohorts treated with CD19 CAR-T cells, underscoring the variability in clinical remission within this subgroup.^21^ To address these limitations, dual-target CAR-T-cell constructs, such as CD19/CD22 or CD19/CD20 combinations, have been developed and have demonstrated enhanced efficacy in r/r B-NHL by mitigating antigen escape and improving CAR-T-cell persistence.^22–27^ These bispecific approaches have yielded more durable remissions than single-antigen CAR-T-cell therapies and are particularly promising for high-risk subsets, including those with TP53 alterations.^28–30^ Furthermore, emerging evidence supports the integration of ASCT with CAR-T-cell therapy as a means of optimizing salvage outcomes in r/r aggressive B-NHL patients.^3,31,32^ With respect to high-risk phenotypes such as double-hit lymphoma and central nervous system involvement, CAR-T-cell therapy plus ASCT has shown encouraging efficacy and acceptable safety, suggesting a potential synergistic effect.^29,30^ This therapeutic integration appears to facilitate in vivo CAR-T-cell expansion and effector differentiation while concurrently mitigating T-cell exhaustion.^3^

Although CAR-T-cell therapy may induce early CR, data specifically addressing TP53-mutated r/r DLBCL remain limited and show discrepancies among reported outcomes,^13,14,21^ largely due to insufficient sample sizes and short follow-up. Consequently, the long-term outcomes and potential for late relapse in this high-risk molecular subgroup remain incompletely characterized. Recent extended follow-up data from landmark clinical trials, including ZUMA-1 and reports from the U.S. Lymphoma CAR-T Consortium, have confirmed the sustained survival benefit of CAR-T-cell therapy in large B-cell lymphoma (LBCL) populations.^33–35^ However, despite these promising outcomes, there remains a clear need for therapeutic refinement. Importantly, these studies did not stratify survival outcomes by TP53 mutation status, leaving the long-term efficacy of CAR-T-cell therapy in this molecularly high-risk subgroup insufficiently defined. In parallel, a recent long-term follow-up analysis^36^ demonstrated that sequential ASCT following CAR-T-cell therapy could improve survival in clinically high-risk patients characterized by SD/PR after salvage or bridging therapy, suggesting the potential value of consolidation strategies in populations with otherwise poor outcomes. Nevertheless, the long-term benefit of such approaches, especially for TP53-mutated disease, remains unknown. Our previous investigations indicated that dual-target CD19/CD22 CAR-T-cell therapy may confer improved disease control in patients with TP53 aberrations.^28^ Furthermore, the sequential administration of CAR-T-cell therapy followed by ASCT has shown promise in enhancing the durability of treatment remission.^28^ In the present study, we report long-term follow-up outcomes from two patient cohorts with TP53-altered r/r aggressive B-NHL treated with CD19/CD22 dual-target CAR-T-cell therapy either as monotherapy or in combination with ASCT. In addition, we present comprehensive safety data, including the incidence of infectious complications and secondary malignancies, and identify prognostic factors relevant to clinical outcomes in this high-risk population.

## Results

### Long-term survival outcomes

A total of 122 patients were enrolled between September 2016 and September 2020. As shown in Table 1, baseline characteristics were generally balanced between cohort A and cohort B, except for the presence of bulky disease, which differed significantly. In addition, although not statistically significant, patients aged older than 60 years tended to be more common in cohort A. Furthermore, baseline characteristics among the four subgroups stratified by TP53 alteration status within cohorts A and B are summarized in Supplementary Table 1. In cohort A, 65 patients received CD19/CD22 dual-target CAR-T-cell therapy alone, 31 (47.7%) of whom carried TP53 alterations. In cohort B, the median interval from ASCT to CAR-T-cell infusion was 3 days (range, 2–6 days). The median CD34⁺ cell dose infused during ASCT was 7.4 (range, 2.2–13.0) ×10⁶/kg. A total of 57 patients received CAR-T-cell therapy following ASCT, among whom 28 (49.1%) harbored TP53 aberrations. A total of 54 patients (44.3%) received maintenance therapy: 33 patients (50.8%) in cohort A and 21 patients (36.8%) in cohort B. These regimens predominantly included programmed cell death protein-1 (PD-1) inhibitors, small-molecule targeted agents, radiotherapy, or combinations, with similar distribution patterns across both study arms (Supplementary Table 2).

**Table 1:** Patient characteristics

Characteristics	cohort A^a^	cohort B^b^	*p*
Patients number	65	57	
Age: ≥60	12 (18.5%)	5 (7.7%)	0.123
Gender (M/F)	42/23	34/23	0.572
ECOG PS			0.679
0–1	41 (63.1%)	38 (66.7%)	
2	24 (36.9%)	19 (33.3%)	
Pathologic subtype			0.760
DLBCL,NOS	45 (69.2%)	40 (70.2%)	
tFL-DLBCL	9 (13.8%)	8 (14.0%)	
HGBL DHL	5 (7.7%)	5 (8.8%)	
HGBL,NOS	3 (4.6%)	0 (0.0%)	
Burkitt lymphoma	2 (3.1%)	2 (3.5%)	
MCL	1 (1.5%)	0 (0.0%)	
Others^c^	1 (1.5%)	2 (3.5%)	
TP53 alteration^d^			0.875
altered	31 (47.7%)	28 (49.1%)	
Bulky disease			0.036
yes	29 (44.6%)	15 (26.3%)	
Disease status			0.426
Primary refractory	17 (26.2%)	20 (35.1%)	
First relapse	22 (33.8%)	20 (35.1%)	
≥2nd relapse	26 (40.0%)	17 (29.8%)	
No.of treatment lines			0.802
2	13 (20.0%)	12 (21.1%)	
3	22 (33.8%)	22 (38.6%)	
≥4	30 (46.2%)	23 (40.4%)	
Baseline LDH ratio			0.409
<1 × ULN	25 (38.5%)	19 (33.3%)	
1–3 × ULN	33 (50.8%)	27 (47.4%)	
>3 × ULN	7 (10.8%)	11 (19.3%)	
Disease stage^e^			0.568
I or II	10 (15.4%)	11 (19.3%)	
III or IV	55 (84.6%)	46 (80.7%)	
IPI score			0.914
0–2	28 (43.1%)	24 (42.1%)	
3–5	37 (56.9%)	33 (57.9%)	

*WT* Wild Type, *ECOG PS* Eastern Cooperative Oncology Group (ECOG) performance status, *DLBCL NOS* diffuse large B-cell lymphoma, not otherwise specified, *tFL-DLBCL* DLBCL transformed from follicular lymphoma (FL), *HGBL DHL* high-grade B-cell lymphoma with MYC and BCL2 and/or BCL6 rearrangements, *HGBL NOS* high-grade B-cell lymphoma, not otherwise specified, *MCL* mantle cell lymphoma, *ASCT* autologous stem cell transplantation, *LDH* lactate dehydrogenase, *ULN* upper limit of normal, *PR* partial response, *SD* stable disease, *PD* progressive disease, *IPI* international prognostic index, *GCB* germinal center B-cell like

^a^Cohort A: patients who received CD19/CD22 CAR-T-cell therapy alone

^b^Cohort B: patients who received CD19/CD22 CAR-T-cell therapy followed by autologous stem cell transplantation (ASCT)

^c^Others, including one B-cell lymphoma, unclassified showing characteristics overlapping DLBCL and classical Hodgkin lymphoma, and one B-cell lymphoblastic lymphoma

^d^Baseline TP53 alteration status was categorized as absent vs altered

^e^Disease stage was determined according to the modified Ann Arbor staging system

**Fig. 1:**
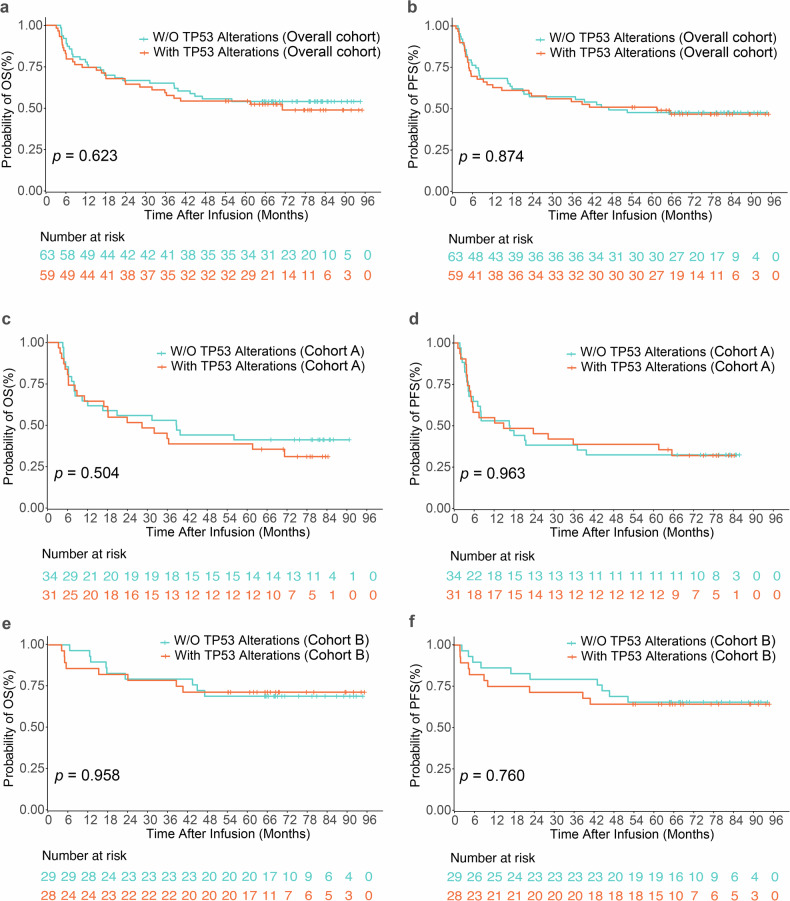
Kaplan–Meier survival analyses of overall survival (OS) and progression-free survival (PFS) according to TP53 alterations. OS (**a**) and PFS (**b**) in the overall cohort; OS (**c**) and PFS (**d**) in cohort A; OS (**e**) and PFS (**f**) in cohort B

As of the data cutoff, with a median follow-up of 77.77 months, the OS and PFS outcomes for patients enrolled in cohorts A and B are presented in Fig. 1. In the overall population, there were no statistically significant differences in OS or PFS between patients with and without TP53 alterations (*p* = 0.623 and 0.874, respectively) (Fig. 1a, b). In cohort A, which evaluated CD19/CD22 CAR-T-cell therapy as monotherapy, the median follow-up was 81.17 months (range, 3.10–89.50 months). Among the 65 patients with r/r B-NHL, the median OS and PFS were 31.90 months (range, 3.10–89.50 months) and 16.53 months (range, 1.07–84.73 months), respectively (Fig. 1c, d). The estimated 5-year OS and PFS rates were 40.0% (95% CI: 29.7–53.8%) and 35.4% (95% CI: 25.5–49.2%), respectively. Among these patients, those harboring TP53 alterations had a median OS and PFS of 28.23 and 14.83 months, respectively, which were not significantly different from those of patients without TP53 alterations (OS: *p* = 0.504; PFS: *p* = 0.963). These findings suggest that dual-target CAR19/22 T-cell therapy may attenuate the adverse prognostic effect associated with TP53 disruption in aggressive B-NHL.

In cohort B, which investigated CAR-T-cell therapy in combination with ASCT, the median follow-up duration was 69.32 months (range, 4.00–94.57 months). For the 57 enrolled patients, the median OS and PFS were not reached. The estimated 5-year OS and PFS rates were 70.2% (95% CI: 59.3–83.1%) and 64.9% (95% CI: 53.6–78.6%), respectively (Fig. 1e, f). Consistent with cohort A, no significant differences in OS or PFS were observed between patients with and without TP53 alterations (OS: *p* = 0.959; PFS: *p* = 0.760), reinforcing the potential of this treatment strategy to overcome the historically poor prognosis associated with TP53 mutations.

Notably, in the overall population (Supplementary Fig. 1a, b) as well as patients with TP53 alterations (Supplementary Fig. 1c, d), those in cohort B exhibited significantly superior OS and PFS compared with those in cohort A, highlighting the potential added benefit of this treatment strategy across the overall patient population, including those with TP53 alterations. In cohort A, 28 of 54 patients who initially achieved CR or partial remission (PR) achieved remission at 24 months. In contrast, in cohort B, 45 of 53 patients experienced remission over the same interval. No statistically significant differences in remission duration were observed between TP53-mutated and wild-type patients in either cohort. Nevertheless, cohort B demonstrated a more favorable overall remission profile.

### Long-term nonrelapse mortality profiles and causes of death

**Fig. 2:**
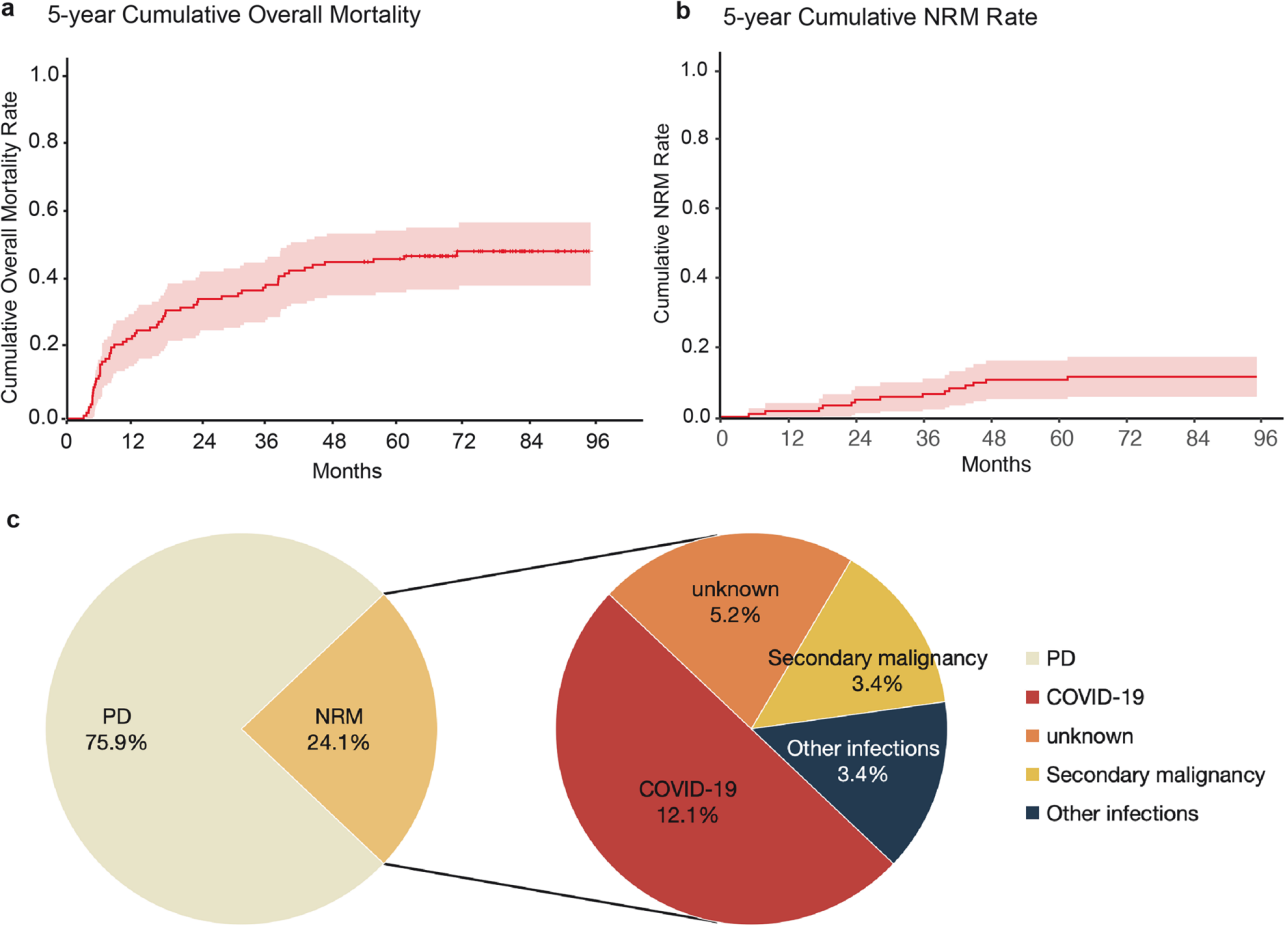
Kaplan‒Meier curves and distribution of causes of mortality. K‒M curves showing the 5-year cumulative overall mortality (**a**) and NRM rates (**b**). **c** Combined pie charts illustrating the distribution of all reported causes of death and the specific causes of nonrelapse mortality (NRM)

As of the final follow-up cutoff, a total of 56 patients had died, corresponding to a 5-year cumulative mortality rate of 45.9% (95% CI: 36.3–54.1%) and a 5-year cumulative nonrelapse mortality (NRM) rate of 10.7% (95% CI: 5.1–16.2%) (Fig. 2a, b). Moreover, the 5-year NRM was 9.2% (95% CI: 2.1–16.4%) in cohort A and 12.3% (95% CI: 3.7–20.9%) in cohort B, with no statistically significant difference between the two cohorts (*p* = 0.576), whereas the 5-year cumulative mortality was significantly lower in cohort B (Supplementary Fig. 2a, c). Cumulative mortality and NRM did not differ significantly between patients with or without TP53 alterations (Supplementary Fig. 2b, d), indicating that TP53 status did not influence long-term NRM.

As shown in Fig. 2c, disease progression remained the predominant cause of death, accounting for 75.9% of the mortality events. COVID-19-related complications contributed to 12.1% of deaths, whereas 5.2% were attributed to unknown causes in patients who were in remission at their last follow-up. These findings may partially reflect underreporting or follow-up loss during pandemic-related lockdowns. Secondary malignancies and other severe infections were each responsible for 3.4% of the deaths.

### Late hematologic toxicity

**Fig. 3:**
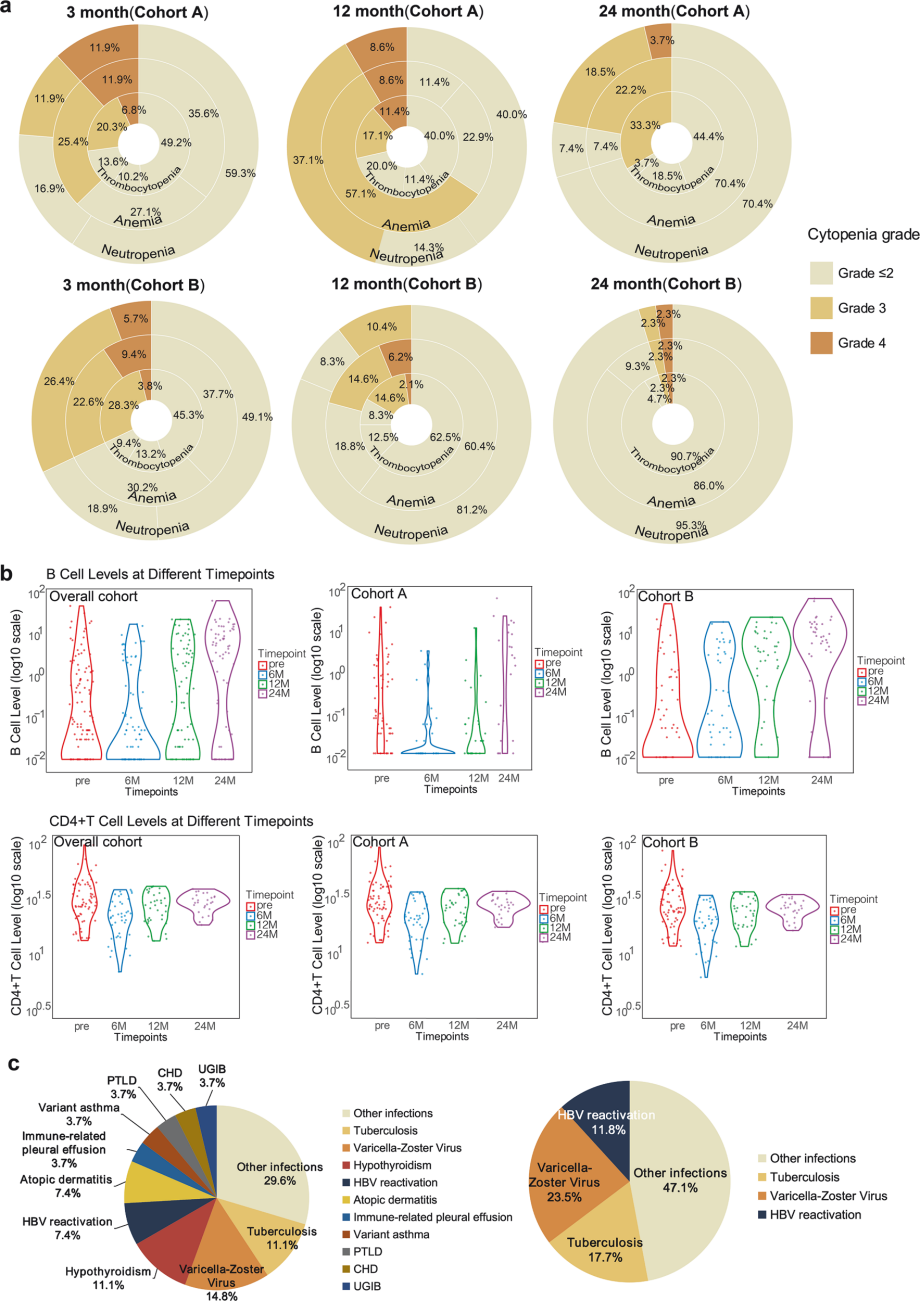
Hematologic toxicity, immune cell dynamics, and late adverse events after CAR-T-cell infusion. **a** Circular charts showing hematologic toxicity grades at 3, 12, and 24 months after infusion in cohorts A and B. **b** Violin plots illustrating B-cell and CD4⁺ T-cell levels preinfusion and at 6, 12, and 24 months post-infusion in the overall population and by cohort. **c** Pie charts displaying the distribution of all late adverse events (AEs) in the overall population (left) and infection types among all patients (right)

The incidence of hematologic toxicity at 3, 12, and 24 months post CAR-T-cell infusion is summarized in Fig. 3a. At 3 months, the rates of grade ≥3 neutropenia (cohort A: 23.7%; cohort B: 32.1%), grade ≥2 thrombocytopenia (cohort A: 40.7%; cohort B: 41.5%), and grade ≥2 anemia (cohort A: 37.3%; cohort B: 32.1%) were comparable between the two treatment arms. By 12 months, neutropenia (45.7% vs. 10.4%) and anemia (65.7% vs. 20.8%) occurred more frequently in cohort A, with a numerically greater incidence of thrombocytopenia (48.6% vs. 25.0%). At 24 months, thrombocytopenia remained more common in cohort A (37.0%) than in cohort B (4.7%), whereas differences in neutropenia (22.2% vs. 4.7%) and anemia (22.2% vs. 4.7%) were less common. Additionally, hematologic toxicity grades did not differ substantially between patients with and without TP53 alterations in either cohort (Supplementary Fig. 3).

### Immune reconstitution and late infections

The immune reconstitution dynamics following CAR-T-cell therapy are depicted in Fig. 3b and Supplementary Fig. 4. Postinfusion, both B-cell counts and CD4⁺ T-cell counts decreased markedly across both cohorts, followed by gradual recovery over time. Notably, patients in cohort B exhibited earlier B-cell reconstitution than those in cohort A did, while CD4⁺ T-cell recovery kinetics were similar between the two cohorts. The CD8⁺ T-cell and NK-cell frequencies remained relatively stable and showed no significant intergroup differences. Additionally, immune reconstitution patterns did not differ substantially between patients with and without TP53 alterations in either cohort.

Beyond the first 3 months following CAR-T-cell infusion, 12.3% (15/122) of patients developed new-onset severe infections necessitating hospitalization or intravenous antimicrobial therapy (Fig. 3c). Pneumonia and bloodstream infections were the most frequent clinical manifestations (15 cases), and a single case of central nervous system infection was also documented. The specific infectious etiologies included three cases of tuberculosis (two pulmonary and one tuberculous meningitis), four cases of herpes zoster, two cases of hepatitis B virus reactivation, and nine cases of COVID-19 infection. Owing to limitations in diagnostic capacity and overlap with other clinical syndromes during the COVID-19 pandemic, the true incidence of SARS-CoV-2 infection may have been underestimated. Infection-related complications resulted in nine deaths, including seven directly attributed to COVID-19. Overall, infections accounted for 64.3% (9/14) of NRM cases (Fig. 2c).

### Secondary malignancies and other late adverse events

Three patients (2.4%) developed secondary malignancies (Supplementary Table 3). One patient was diagnosed with acute monocytic leukemia (AML-M5) after receiving transplantation followed by sequential CAR-T-cell therapy; this patient achieved remission following AML-directed treatment at an external institution and remains alive. A second patient was diagnosed with gastrointestinal adenocarcinoma following pleural effusion biopsy and opted for palliative management. The third patient was diagnosed with poorly differentiated squamous cell carcinoma of the left upper lung and received combination chemotherapy and radiotherapy.

In addition to infectious complications, other severe late-onset adverse events (AEs) are associated primarily with immune dysregulation (Supplementary Fig. 5). These included hypothyroidism (*n* = 3) and atopic dermatitis (*n* = 2). Additional isolated events included immune-mediated pleural effusion, asthma, posttransplant lymphoproliferative disorder (PTLD), and coronary artery disease. All patients with these complications received appropriate therapeutic management and subsequently recovered. The incidence and distribution of these late AEs did not differ significantly between the two cohorts or different TP53 statuses (Supplementary Tables 4, 5).

### Key predictors of poor long-term prognosis

Univariate and multivariate Cox proportional hazards regression analyses were performed to identify clinical and treatment-related variables associated with OS and PFS in the entire study population (Table 2) or in either cohort (Supplementary Tables 6, 7). In the total population, factors including treatment option, Eastern Cooperative Oncology Group (ECOG) performance status, and the presence of bulky disease demonstrated significant associations with both survival measures according to univariate analysis. Notably, TP53 mutation-related variables were not significantly associated with survival in the univariate analysis. Multivariate analysis revealed that treatment option (OS: HR = 2.15 95% CI: 1.19–3.89, *p* = 0.011; PFS: HR = 2.09, 95% CI: 1.20–3.65, *p* = 0.009) and the presence of bulky disease were independent predictors of inferior OS and PFS.

**Table 2:** Univariate and multivariate Cox regression analyses of prognostic factors associated with 5-year overall survival (OS) and progression-free survival (PFS) in the overall study population

Variable	OS Univariate		OS Multivariate		PFS Univariate		PFS Multivariate	
	HR (95% CI)	*p*	HR (95% CI)	*p*	HR (95% CI)	*p*	HR (95% CI)	*p*
Sex: Male vs. Female	1.32 (0.76–2.29)	0.318			1.22 (0.73–2.04)	0.454		
Age: <60 vs. ≥60	1.52 (0.79–2.94)	0.210			1.67 (0.89–3.13)	0.110		
Treatment option: cohort A vs. cohort B	2.82 (1.60–4.97)	<0.001	2.15 (1.19–3.89)	0.011	2.74 (1.61–4.66)	<0.001	2.09 (1.20–3.65)	0.009
ECOG PS: 0–1 vs. 2	2.11 (1.25–3.55)	0.005	1.69 (0.99–2.87)	0.054	2.03 (1.24–3.33)	0.005	1.63 (0.98–2.71)	0.060
Bulky disease: Present vs. Absent	2.23 (1.33–3.73)	0.002	1.80 (1.06–3.04)	0.029	2.26 (1.38–3.70)	0.001	1.86 (1.13–3.08)	0.015
TP53 altered: Present vs. Absent	1.14 (0.68–1.90)	0.624			1.04 (0.64–1.70)	0.874		
Double expression: Present vs. Absent	1.36 (0.80–2.32)	0.260			1.26 (0.76–2.09)	0.371		
CNS involvement: Present vs. Absent	1.50 (0.68–3.30)	0.318			1.26 (0.58–2.77)	0.561		
Burkitt lymphoma: Yes vs. No	1.07 (0.26–4.40)	0.922			1.01 (0.25–4.15)	0.984		
Double-Hit: Present vs. Absent	1.29 (0.65–2.55)	0.469			1.09 (0.56–2.14)	0.802		
TP53 missense mutation groups: Nonmissense vs. Missense	0.98 (0.41–2.35)	0.962			1.14 (0.48–2.70)	0.767		
TP53 disruptive mutation groups: Nondisruptive vs. Disruptive	1.77 (0.80–3.93)	0.158			1.45 (0.68–3.09)	0.338		
EAp53 score: High risk vs. Low risk	0.76 (0.30–1.95)	0.569			0.62 (0.26–1.51)	0.294		
Del(17p): Yes vs. No	0.94 (0.37–2.39)	0.897			0.67 (0.29–1.55)	0.347		
Treatment line number : 2 lines vs. ≥3 lines	1.08 (0.55–2.14)	0.815			1.10 (0.58–2.11)	0.770		

*OS* overall survival, *PFS* progression-free survival, *HR* hazard ratio, *CI* confidence interval, *ECOG PS* Eastern Cooperative Oncology Group performance status, *TP53* altered any TP53 mutation or deletion, *Double expression* concurrent MYC and BCL2 overexpression, *CNS* central nervous system, *Double hit* lymphoma with MYC and BCL2/BCL6 rearrangements, *Del(17p)* deletion of chromosome 17p, *EAp53 score* evolutionary action score of TP53

To assess the prognostic value of posttreatment factors, a 3-month landmark analysis was performed (Supplementary Table 8). Univariate analysis revealed that treatment options, bulky disease, NRR3 (not reaching remission at 3 months), and cytokine release syndrome (CRS) demonstrated statistical relevance to both OS and PFS, whereas ECOG performance status predicted worse OS, and age ≥60 years was linked to poorer PFS. In the multivariate analysis, treatment option and NRR3 remained independent predictors of inferior OS and PFS.

## Discussion

In this study, we conducted an extended follow-up analysis to assess the long-term efficacy of CD19/CD22 dual-target CAR-T-cell therapy, administered either as monotherapy or in combination with ASCT, in patients with r/r aggressive B-NHL harboring TP53 alterations. With a median follow-up duration of 77.77 months, this investigation offers one of the most comprehensive longitudinal evaluations to date in this high-risk patient population.

In the present study, patients receiving CAR-T-cell therapy alone (cohort A) had 5-year OS and PFS rates of 40.0% and 35.4%, respectively. In contrast, those treated with CAR-T-cell therapy in combination with ASCT (cohort B) achieved improved outcomes, with 5-year OS and PFS rates of 70.2% and 64.9%, respectively. Importantly, within both treatment cohorts, patients with TP53 alterations presented survival outcomes comparable to those of their TP53 wild-type counterparts. To provide additional context for the comparison between cohorts, we performed statistical analyses to account for potential confounders, including univariate and multivariate Cox regression as well as supplementary landmark analyses. Baseline characteristics were generally balanced between cohorts, which supports the reliability of these comparisons. Although these findings are observational, they indicate a meaningful potential benefit of integrating ASCT with CAR-T-cell therapy.

To place these results in context, we summarized previously reported CAR-T-cell studies with a median follow-up exceeding 60 months (Table 3).^33,35^ Among the limited long-term datasets currently available, only ZUMA-1 and the U.S. Lymphoma CAR-T Consortium, with 5-year OS and PFS rates ranging from 40–43% and 29–36%, respectively, in broader unselected patient populations,^33,35^ have reported outcomes beyond 5 years. No prior study has evaluated either the combination of CAR-T-cell therapy with ASCT or long-term outcomes, especially in patients harboring TP53 alterations. Our findings suggest that dual-target CAR-T-cell therapy alone may achieve disease control comparable to that reported in broader r/r LBCL cohorts, even among patients harboring TP53 aberrations, potentially offset the historically adverse impact of TP53 mutations. In addition, the addition of ASCT appears to further enhance long-term outcomes in the high-risk subgroup.

**Table 3:** Overview of CAR-T-cell therapy long-term follow-up in patients with B-cell non-Hodgkin lymphoma (B-NHL)^a^

Study	Treatment strategy	Patient population (*n*)	Median follow-up (range), months	5- year OS	5- year PFS	Secondary malignancies	NRM rate	Author, year
ZUMA-1	Axi-cel (CD19)	r/r LBCL, *n* = 101	63.1 (58.9–68.4)	42.6%	31.8%	1 (1%)	14 (14%)	Neelapu et al., Blood 2023^35^
US Lymphoma CAR-T Consortium	Axi-cel (CD19)	r/r LBCL, *n* = 275	58.0 (0.16–68.7)	40%	29%	24 (9%)	16.2% (5-year)	Jain et al., J Clin Oncol 2024^33^
ZUMA-5	Axi-cel (CD19)	r/r iNHL, *n* = 159	64.6 (32.3–81.4)	69.0%	50.4%	6 (4%)	33 (21%)	Neelapu et al., J Clin Oncol. 2025^48^
Present study	CD19/CD22 CAR-T ± ASCT	r/r B-NHL, *n* = 122	77.77 (3.10–95.07)	54.1% in overall cohort 70.2% in cohort B	49.2% in overall cohort 64.9% in cohort B	3 (2%)	10.7% (5-year)	Present study

*Axi-cel* axicabtagene ciloleucel, *r/r* relapsed or refractory, *LBCL* large B-cell lymphoma, *iNHL* indolent non-Hodgkin lymphoma, *B-NHL* B-cell non-Hodgkin lymphoma, *OS* overall survival, *PFS* progression-free survival, *NRM* nonrelapse mortality

^a^Studies with a median follow-up of ~5 years or longer were included, providing long-term outcome data on efficacy and safety

In aggressive B-NHL, TP53 mutations disrupt the canonical tumor suppressor functions of the “guardian of the genome,” impairing apoptosis, DNA repair mechanisms, and genomic stability.^5,6,37^ These molecular aberrations contribute to intrinsic resistance against both standard chemotherapy and intensified regimens, including HDT followed by ASCT.^8,18^ Additionally, TP53 dysregulation has been implicated in the downregulation of CD19 antigen density on malignant B cells, facilitating antigen-negative relapse and reducing the efficacy of single-target CD19-directed CAR-T-cell therapies.^13,33^

Current evidence from CAR-T studies in patients with TP53-mutated r/r B-NHL primarily reports early remission and short-term survival outcomes, yielding heterogeneous conclusions regarding the prognostic significance of TP53 alterations.^13^ However, the long-term outcomes and potential for late relapse in this high-risk molecular subgroup remain incompletely characterized, emphasizing the importance of extended follow-up. Seminal CAR-T trials such as ZUMA-1 and ZUMA-7, along with 5-year follow-up data from the U.S. Lymphoma CAR-T Consortium, have established benchmark survival rates in unselected cohorts of LBCL patients treated with CAR-T-cell therapy. However, none of these studies have stratified outcomes on the basis of TP53 mutational status.^33,34,38^ To the best of our knowledge, the present study represents the first report to delineate survival outcomes beyond 5 years in patients with TP53-altered r/r aggressive B-NHL receiving CAR-T-cell therapy.

Dual-target CD19/CD22 CAR-T-cell constructs offer mechanistic advantages by concurrently engaging two distinct B-cell surface antigens, thereby mitigating the risk of antigen escape and enhancing the stability of immune synapse formation. This dual-antigen recognition broadens tumor coverage in the context of heterogeneous antigen expression and facilitates sustained CAR-T-cell activation. These features are associated with improved remission durability and reduced relapse rates compared with traditional single-target CAR-T-cell therapies.^22–27,39^ Moreover, the integration of sequential ASCT following CAR-T-cell infusion may confer additional therapeutic benefits. This strategy facilitates effective tumor debulking, potentially eliminates CAR-T-resistant malignant clones, and induces a myeloablative reset of the immune microenvironment, factors that may enhance CAR-T-cell persistence and functional efficacy.^3,40^ The favorable long-term outcomes observed in patients receiving CAR-T-cell therapy combined with ASCT indicate that this integrated approach may confer broader survival benefits, especially in patients with TP53 alterations who have historically faced poor prognoses. Our extended follow-up revealed a 5-year cumulative NRM rate of 10.7%, with infection-related complications accounting for 64.3% of NRM events and secondary malignancies accounting for 14.3% of NRM events. Notably, NRM did not significantly differ between the CAR-T-cell monotherapy (cohort A) and CAR-T-cell plus ASCT (cohort B) arms, nor was it influenced by the TP53 mutational status. These findings compare favorably with the 5-year NRM of 16% reported in real-world cohorts receiving axi-cel, as well as with a pooled NRM estimate of 16.2% from systematic reviews of long-term CAR-T survivors, wherein infections and secondary malignancies accounted for 50.9% and 7.8% of NRM, respectively.^33,41^ Similarly, the incidence of secondary primary malignancies in our cohort (2.5%) aligns with that reported in recent studies of lymphoma and myeloma CAR-T recipients, such as the 4.9% rates reported by Tix et al.^42^ These data support the notion that dual-target CD19/CD22 CAR-T-cell therapy, whether administered alone or in conjunction with ASCT, maintains a long-term safety profile that is comparable to that of established single-target CAR-T-cell regimens in high-risk B-NHL patients.

Despite the strengths of this analysis, several limitations warrant consideration. The retrospective, single-center nature of the study, the relatively modest sample size, and heterogeneity in therapeutic regimens may limit the generalizability of our findings. Patient assignment to treatment arms was nonrandomized and influenced by clinical parameters such as performance status, tolerance to HDT, and success of stem cell mobilization, factors that may introduce selection bias. Moreover, the administration of maintenance therapies in a subset of patients across both cohorts presents a potential confounder in isolating the true efficacy of CAR-T-cell monotherapy versus combination treatment with ASCT. While evidence remains limited, preclinical studies suggest that TP53 loss may enhance programmed cell death protein 1 (PD-1)/programmed death-ligand 1–mediated immune escape (PD-L1)–mediated immune escape, providing a potential biological rationale for checkpoint inhibition in this context.^43^ Future prospective, multicenter trials are required to further substantiate these results and optimize treatment algorithms for this population with poor prognostic characteristics.

In summary, this long-term follow-up study provides the first comprehensive evidence that dual-target CD19/CD22 CAR-T-cell therapy, particularly when combined with ASCT, achieves durable survival outcomes with a consistent safety profile in patients with TP53-mutated r/r aggressive B-NHL. The comparable 5-year OS and PFS rates observed between TP53-mutant and wild-type patients within each treatment cohort, along with the significant survival advantage noted in the CAR-T-cell therapy plus ASCT arm, underscore the curative potential of this therapeutic combination. As the therapeutic landscape of B-NHL continues to evolve, with the emergence of antibody‒drug conjugates, bispecific antibodies, and next-generation CAR-T platforms, it remains to be determined whether these novel immunotherapeutic agents can further circumvent TP53-mediated resistance. Continued research integrating molecular risk stratification with advanced cellular therapies will be critical for improving outcomes in patients with aggressive, TP53-altered B-NHL.

## Materials and methods

### Study design

This was a single-center, investigator-initiated study comprising two independent clinical trials designed to evaluate the efficacy and safety of dual-target CD19/CD22 CAR-T-cell therapy administered either as monotherapy (cohort A) or in combination with ASCT (cohort B) in patients with r/r aggressive B-NHL in whom TP53 mutation and deletions of chromosome 17p [del(17p)] were screened through next-generation sequencing or fluorescence in situ hybridization.^28^ The inclusion and exclusion criteria for both trials have been previously published.^28^ Clinical remission rates, long-term efficacy outcomes, safety parameters, and immune cell subset profiles were comprehensively evaluated in relation to disease and treatment characteristics. Ethical clearance for both studies was provided by the Institutional Review Board of Tongji Hospital, Tongji Medical College, Huazhong University of Science and Technology. The studies were additionally recorded in the Chinese Clinical Trial Registry (ChiCTR) under the identifier ChiCTR-OPN-16008526 and ChiCTR-OPN-16009847. Written informed consent was obtained from all participants, and all study procedures conformed to the ethical principles outlined in the Declaration of Helsinki.

### Procedures and long-term follow-up

Leukapheresis was performed for the generation of autologous CAR-T-cell products. In cohort A, patients received a lymphodepletion regimen comprising fludarabine and cyclophosphamide prior to CAR-T-cell infusion. In cohort B, hematopoietic stem-cell mobilization and collection were completed before lymphocyte leukapheresis to adapt to the clinical conditions. The participants then underwent BEAM (carmustine, etoposide, cytarabine, and melphalan) conditioning, which also served as lymphodepleting therapy, followed by ASCT (designated day 0). CAR-T cells were subsequently infused between day +2 and day +6 after stem-cell reinfusion. CRS was graded according to the criteria established by Lee et al.^44^, whereas immune effector cell-associated neurotoxicity syndrome (ICANS) was assessed in accordance with the American Society for Transplantation and Cellular Therapy (ASTCT) Consensus Guidelines and the National Cancer Institute’s Common Terminology Criteria for Adverse Events (CTCAE) version 5.0.^45,46^ Disease staging and remission assessments were conducted on the basis of the National Comprehensive Cancer Network (NCCN) Guidelines and the Lugano Classification for lymphoma remission evaluation. As part of the investigator-initiated trial framework, maintenance therapy was administered at the discretion of the treating physician.

Patients were monitored until death, loss to follow-up, or withdrawal of consent. For this analysis, data collection was finalized on December 31, 2024. Late AEs were defined as new or ongoing toxicities first emerging at or beyond 3 months after CAR-T-cell infusion. The surveillance window for late AEs extended from 3 months post-infusion until disease progression, patient death, loss to follow-up, or 5 years, whichever occurred first. The classification of late AEs followed the framework proposed by Camacho-Arteaga et al.^47^ Immune reconstitution was assessed by serial quantification of peripheral immune cell subsets, including B cells, CD4⁺ T cells, CD8⁺ T cells, and natural killer (NK) cells, at baseline and at 6, 12, and 24 months post-infusion. Monitoring continued until disease relapse, death, or completion of a 2-year follow-up period, after which patients were not routinely evaluated. Hematologic toxicity was tracked through peripheral blood counts at 3, 12, and 24 months following CAR-T-cell therapy. The definitions of cytopenias were as follows: grade ≥3 neutropenia (absolute neutrophil count <1000/μL), grade ≥2 thrombocytopenia (platelet count <75 × 10⁹/L), and grade ≥2 anemia (hemoglobin <10 g/dL). Severe long-term complications were prospectively documented, excluding NRM, and defined as events necessitating hospitalization, including secondary malignancies, serious infections, and autoimmune conditions, given their relevance to post-CAR-T-cell safety. All AEs were categorized and graded per the CTCAE version 5.0 criteria, with particular attention given to severe infections (e.g., pneumonia, bacteremia, and central nervous system infections) and secondary malignancies. These complications were recorded during scheduled follow-up visits, which were typically conducted every 3–6 months. The management and outcomes of severe events were captured from clinical documentation and institutional assessments.

### Statistical analysis

The unpaired two-tailed Student’s *t* test was applied for comparisons of continuous data, whereas categorical variables were examined via either the chi-square test or Fisher’s exact test, as appropriate. The ordinal data were evaluated via rank sum (Mann‒Whitney *U*) tests. OS and PFS were defined as the intervals from CAR-T-cell infusion to death and disease progression, respectively. ORRs were calculated along with their corresponding 95% confidence intervals (CIs) via the Clopper‒Pearson method. Survival distributions were estimated via the Kaplan‒Meier method, and intergroup comparisons were assessed via the log-rank test. In the presence of competing risks, the cumulative incidence function (CIF) method was applied to estimate the cumulative incidence of NRM, and Gray’s test was used to compare CIF curves between groups.

Cox proportional hazards regression models were employed for both univariate and multivariate analyses to identify prognostic factors. Variables with *P* <0.1 in univariate analyses, as well as clinically relevant covariates (including treatment group, ECOG performance status, and bulky disease), were included in multivariate models. A 3-month landmark Cox analysis was additionally performed to minimize bias from early events and to assess long-term outcomes.

A two-sided *P* <0.05 was considered statistically significant. Analyses were performed via SPSS version 22.0 (IBM Corp., Chicago, IL, USA). K‒M analyses for OS and PFS were conducted with the “survminer” package (version 4.3.3) in R software (version 4.3.1), and violin plots for immune reconstitution were generated via “ggplot2” (version 3.5.1).

### Ethics approval

This study was approved by the Ethics Committee of Tongji Hospital, Tongji Medical College, Huazhong University of Science and Technology (Approval No. TJ-IRB202506040), in accordance with Good Clinical Practice and all applicable laws and regulations. All methods were carried out in accordance with relevant guidelines and regulations. Written informed consent was obtained from all patients.

## Supplementary information

Study protocols

Supplementary Materials

## Data Availability

The raw clinical data are protected and are not available due to data privacy laws. The study protocols are provided in the Supplementary Materials. Deidentified individual participant data supporting the findings described in this article will be accessible following publication for a period of up to 6 years. Data will be provided to researchers submitting scientifically sound proposals. Interested researchers should contact the corresponding author directly to request access.
