# Supplementary material for: Long-term follow-up demonstrates the curative potential of dual CD19/CD22 CAR-T-cell therapy alone or combined with autologous stem cell transplantation in TP53-altered relapsed/refractory B-cell non-Hodgkin lymphoma
Source: Signal Transduct Target Ther. 2026 Feb 13;11:53. doi: 10.1038/s41392-025-02571-7 (PMC12902020; doi:10.1038/s41392-025-02571-7)
Supplement: Supplementary file 1 — Study protocols [file 41392_2025_2571_MOESM1_ESM.pdf]

# Protocol

## An open-label, single-center, single-arm clinical study evaluating sequential infusion of anti-CD22 and anti-CD19 CAR T-cell (CAR19/22 T-cell) in patients with relapsed or refractory (r/r) B-cell malignancies

**Registration number:** ChiCTR-OPN-16008526

**PI/Department:** Professor Jianfeng Zhou /Department of Hematology.

**Study sponsor and monitor:** Tongji Hospital, Tongji Medical College, Huazhong University of Science and Technology

**Technical Partners:** Wuhan Bioraid Co., Ltd.

**declaration of secrecy**

This protocol is classified as a confidential document. All information contained herein is the property of the research institution (Tongji Hospital Affiliated to Tongji Medical College, Huazhong University of Science and Technology) and is provided exclusively for review by the investigators, collaborating researchers, the ethics committee, and relevant regulatory authorities. No dissemination or disclosure is permitted without prior written approval from the research institution.

# catalogue

Abstract ........ 3
Treatment Flowchart ........ 8
I. Research Background ........ 19
II. Research Objectives ........ 22
III. Research Plan ........ 22
3.1 Overview of the Research Plan ........ 22
3.2 Rationale for the Research Plan ........ 24
IV. Study Population ........ 24
4.1 Eligibility Criteria ........ 24
4.2 Exclusion Criteria ........ 26
4.3 Withdrawal Criteria ........ 26
4.4 Suspension Criteria ........ 27
V. Treatment Protocol ........ 27
5.1 Patient Screening, Cell Collection, Modification, and Amplification ........ 28
5.2 Non-myeloablative pretreatment ........ 28
5.3 CAR-T Cell Infusion ........ 29
5.4 Adverse Reactions and Management ........ 29
5.5 Monitoring and Follow-up ........ 31
VI. Concomitant Therapy and Contraindicated Drugs ........ 32
VII. Types of Trial Designs and Blinding Methods ........ 32
VIII. Treatment Compliance ........ 32
IX. Evaluation Indicators and Criteria ........ 33
9.1 Primary End Points ........ 33
9.2 Secondary End Points ........ 33
9.3 Safety Assessment ........ 34
X. Data Management ........ 38
10.1 Case Report Filling and Handover ........ 38
10.2 Data Entry and Modification ........ 38
10.3 Data Review and Locking ........ 38
XI. Statistical Analysis ........ 39
11.1 Determination of Sample Size ........ 39
11.2 Statistical Analysis Population ........ 39
11.3 Statistical Analysis Plan ........ 39
XII. Quality Assurance and Quality Control ........ 42
XIII. Ethical Requirements and Informed Consent of Subjects ........ 44
XIV. Provisions on the Preservation and Ownership of Clinical Research Data ........ 45
XV. References ........ 46

# Abstract

| | |
|---|---|
| **research topic** | An open-label, single-center, single-arm clinical study evaluating sequential infusion of anti-CD22 and anti-CD19 CAR T-cell (CAR19/22 T-cell) in patients with relapsed or refractory (r/r) B-cell malignancies |
| **purpose of research** | Primary research objectives:<br>To evaluate the safety of sequential infusion of anti-CD19 CAR-T and anti-CD22 CAR-T in the treatment of r/r B-cell malignancies.<br>Secondary research objectives:<br>1. To evaluate the clinical efficacy of sequential infusion of anti-CD19 CAR-T and anti-CD22 CAR-T in the treatment of r/r B-cell malignancies;<br>2. To evaluate the expansion effect and persistence of anti-CD19 CAR-T/anti-CD22 CAR-T cells in the subjects. |
| **research design** | An open-label, single-center, single-arm clinical study |
| **Subject** | 500 cases of B-cell malignancies patients. |
| **trial period** | including screening, treatment and follow-up.<br>The screening and enrollment phase lasted 1 week.<br>The observation period lasted until 6 months after reinfusion (short-term follow-up).<br>The medium-and long-term follow-up is expected to be 1 year. |
| **Inclusion criteria** | Participants must meet all of the following:<br>(1) Signed informed consent obtained before any study procedures.<br>(2) Age ≥ 18 years.<br>(3) Pathologically confirmed $CD19^+/CD22^+$ B-ALL or B-NHL.<br>(4) Relapsed or refractory B-cell malignancy, including: B-ALL; Aggressive B-cell lymphomas: DLBCL, BL, MCL, or transformed B-cell lymphoma.<br>(5) At least one of the following: |

| | |
|---|---|
| | a) Failed ≥2 lines of salvage therapy<br>b) Relapsed/refractory post-HSCT.<br>c) High-risk features (e.g., double -hit lymphoma)<br>d) Ineligible for allo-HSCT (B-ALL) or auto-HSCT (B-NHL)<br>(6) At least one measurable lesion.<br>(7) Adequate organ function, defined as creatinine<2.5mg/dl; aspartate transaminase/ alanine transaminase <3 × upper limit of normal; SiO2 ≥ 95%; bilirubin < 2.0 mg/dl; LVEF > 40%.<br>(8) Sufficient venous access for leukapheresis.<br>(9) ECOG performance status ≤ 2.<br>(10) Estimated survival of ≥ 3 months. |
| **exclusion criteria** | 1. Pregnant or lactating women, or planning pregnancy within 1 year<br>2. Active HBV, HCV, or HIV infection.<br>3. Uncontrolled systemic infections.<br>4. Systemic steroid use within 4 weeks.<br>5. Known allergy to cytokines or antibodies.<br>6. Participation in other clinical trials within 6 weeks.<br>7. Active graft-versus-host disease.<br>8. History of psychiatric disorders or another primary malignancy<br>9. Substance abuse or addiction.<br>10. Other conditions deemed unsuitable by the investigator. |
| **Test** | Test products: anti-CD19 CAR-T cells; anti-CD22 CAR-T cells; |

| | |
|---|---|
| **product** | Reinfusion dose: The investigator determines the reinfusion dose based on the subject's individual/disease condition and in vitro preparation status.<br><br>Administration route: Intravenous infusion/push for 30 minutes. |
| **stages of research** | 1. Screening period (Visit 1: -21 to-7 days, duration 2 weeks): Participants sign the informed consent form at Visit 1 and complete a series of examinations (see Flowchart Visit 1 for details). The investigator evaluates whether the participant meets the inclusion criteria based on the examination results and exclusion criteria, ensuring no participant fails to meet the exclusion criteria. Additionally, the investigator determines whether partial examination results from the month prior to the informed consent date can be accepted, depending on the participant's condition.<br>2. Non-myeloablative conditioning (Visit 2-3: Days-4 to-1, 4-day duration): The necessity and regimen of conditioning are determined based on the patient's condition, with the FC regimen being the most commonly used. After returning to the research center for hospitalization as notified by the investigator, patients receive fludarabine and cyclophosphamide therapy from Days-4 to-2 of the trial. On Day-1, the regimen is suspended for 1 day, and a series of examinations (see Flowchart Visit 3) are completed to obtain baseline information for patients undergoing non-myeloablative conditioning.<br>3. Short-term follow-up period (Visits 4-10:0-180 days, duration 6 months): Subjects returned to the research center monthly for a series of examinations (see Flowchart Visits 5-10) within 6 months after receiving the expected dose of anti-CD19 CAR-T/anti-CD22 CAR-T cells on Days 0 and 2 of the trial. Investigators evaluated the short-term safety and efficacy of the anti-CD19 CAR-T/anti-CD22 CAR-T therapy based on the subjects' examination results. |

| | |
|---|---|
| | 4. Medium-to long-term follow-up period (Visits 11-13:180-360 days, duration 6 months): Within 6 months after completing the short-term follow-up, participants return to the research center every 2 months for a series of examinations (see the flowchart for Visits 11-13). Investigators will evaluate the medium-to long-term safety and efficacy of the anti-CD19 CAR-T/anti-CD22 CAR-T therapy based on the participants' examination results.<br>5. Withdrawal from Visit (at Any Time): All subjects may withdraw from the study at any stage of the trial, regardless of providing a reason. If a subject withdraws from the study after receiving anti-CD19 CAR-T/anti-CD22 CAR-T cell infusion, the withdrawal requirements specified in the visit flowchart must be completed. If a subject withdraws from the visit before receiving anti-CD19 CAR-T/anti-CD22 CAR-T cell infusion, only the safety check items need to be completed, and the investigator must document the reason for withdrawal in detail. If a subject discontinues treatment due to an adverse event (AE), the investigator must conduct necessary safety follow-up until the AE returns to baseline levels or reaches a stable state. |
| **evaluating indicator** | Main evaluation indicators:<br>The incidence of treatment-related adverse events (grade 3 or 4 adverse reactions, CTCAE V4.0 criteria). This includes adverse events associated with anti-CD19 CAR-T/anti-CD22 CAR-T therapy, serious adverse events, and clinically significant laboratory abnormalities.<br>Secondary evaluation indicators:<br>1. Objective response rates (ORR) of tumors at 3 months, 6 months, and 1 year post-treatment follow-up: complete response (CR) and partial response (PR);<br>2. overall survival (OS); |

| | |
|---|---|
| | 3. progression-free survival (PFS);<br>4. event-free survival (EFS);<br>5. The temporal variation characteristics of anti-CD19 CAR-T/anti-CD22 CAR-T cell expansion levels in subjects (peripheral blood, bone marrow, cerebrospinal fluid, and lymph nodes, etc.).<br>6. the duration of anti-CD19 CAR-T/anti-CD22 CAR-T cells 'persistence in the subject's body (peripheral blood, bone marrow, cerebrospinal fluid, and lymph nodes, etc.)<br>7. The characteristics of lymphocyte depletion in the subjects. |
| **statistical analysis** | Statistical analysis was performed with SAS 9.2 software.<br>The primary and secondary study endpoints were analyzed using the ITT (Intention-to-Treat) population. The statistical analysis for the primary endpoint involved describing the number of treatment-related adverse events and their incidence rates, as well as comparing the differences in vital signs and laboratory parameters before and after treatment to determine whether these differences were statistically significant.<br>The statistical analysis method for the secondary endpoint was to describe the tumor ORR based on different follow-up time points. The Kaplan-Meier method and survival curves were used to describe OS, PFS, and EFS. Curves were plotted to illustrate the temporal changes in the expansion levels of anti-CD19 CAR-T and anti-CD22 CAR-T cells, the characteristics of B/T lymphocyte depletion in vivo, and the replication patterns of retroviruses in vivo, with quantitative descriptions of their persistence duration. |

# Treatment Flowchart

| | Filter period | Non-myeloablative conditioning | | Short-term follow-up period (monthly) | | | | | | | Medium to long-term follow-up period (every two months) | | | Exit follow-up |
|---|---|---|---|---|---|---|---|---|---|---|---|---|---|---|
| **Visit Point** | Visit 1 | Visit 2 | Visit 3 | Visit 4 | Visit 5 | Visit 6 | Visit 7 | Visit 8 | Visit 9 | Visit 10 | Visit 11 | Visit 12 | Visit 13 | Visit 14 |
| **Visit time window** | -21 | -4 ~ -2 | -1 | 0&2 | 30 ± 5 | 60 ± 5 | 90 ± 5 | 120 ± 5 | 150 ± 5 | 180 ± 5 | 240 ± 5 | 300 ± 5 | 360 ± 5 | at any time |
| **informed consent** | X | | | | | | | | | | | | | |
| **Evaluation of eligibility criteria** | X | | X | | | | | | | | | | | |
| **Demographic information** | X | | | | | | | | | | | | | |
| **anamnesis** | X | | | | | | | | | | | | | |
| **Previous treatment history** | X | | | | | | | | | | | | | |
| **Vital signs, physical** | X | X | X | X | X | X | X | X | X | X | X | X | X | X |

| | Filter period | Non-myeloablative conditioning | | Short-term follow-up period (monthly) | | | | | | | Medium to long-term follow-up period (every two months) | | | Exit follow-up |
|---|---|---|---|---|---|---|---|---|---|---|---|---|---|---|
| **Visit Point** | **Visit 1** | **Visit 2** | **Visit 3** | **Visit 4** | **Visit 5** | **Visit 6** | **Visit 7** | **Visit 8** | **Visit 9** | **Visit 10** | **Visit 11** | **Visit 12** | **Visit 13** | **Visit 14** |
| **Visit time window** | **-21** | **-4 ~ -2** | **-1** | **0&2** | **30 ± 5** | **60 ± 5** | **90 ± 5** | **120 ± 5** | **150 ± 5** | **180 ± 5** | **240 ± 5** | **300 ± 5** | **360 ± 5** | **at any time** |
| **examination [1]** | | | | | | | | | | | | | | |
| **Physical Examination [2]** | X | X | X | X | X | X | X | X | X | X | X | X | X | X |
| **Neurological evaluation [3]** | X | X | X | X | X | X | X | X | X | X | X | X | X | X |
| **Blood Pregnancy Test** | X | | | | | | | | | | | | | X |
| **Complete Blood Count [4]** | X | | | X | X | X | X | X | X | X | X | X | X | X |
| **Blood Biochemistry [5]** | X | | | X | X | X | X | X | X | X | X | X | X | X |
| **coagulation function** | X | | | X | X | X | X | X | X | X | X | X | X | X |
| **electrocardiogram** | X | | | X | X | | X | | X | | X | X | X | X |
| **CD19 and CD22 testing** | X | | | | X | X | X | X | X | X | X | X | X | X |

| | Filter period | Non-myeloablative conditioning | | Short-term follow-up period (monthly) | | | | | | | Medium to long-term follow-up period (every two months) | | | Exit follow-up |
|---|---|---|---|---|---|---|---|---|---|---|---|---|---|---|
| Visit Point | Visit 1 | Visit 2 | Visit 3 | Visit 4 | Visit 5 | Visit 6 | Visit 7 | Visit 8 | Visit 9 | Visit 10 | Visit 11 | Visit 12 | Visit 13 | Visit 14 |
| Visit time window | -21 | -4 ~ -2 | -1 | 0&2 | 30 ± 5 | 60 ± 5 | 90 ± 5 | 120 ± 5 | 150 ± 5 | 180 ± 5 | 240 ± 5 | 300 ± 5 | 360 ± 5 | at any time |
| [6] | | | | | | | | | | | | | | |
| HBV, HCV, and HIV [7] | X | | | | | | | | | X | | | | |
| Peripheral blood EBV-DNA titer [8] | X | | | | X | | X | | X | | X | X | X | X |
| Detection of lentiviral copy number in vivo (q-PCR) [9] | X | | | | X | X | X | X | X | X | X | X | X | X |
| Plain head CT scan | X* | | | | | | | | | | | | | |
| Pulmonary function test [10] | X* | | | | X* | | X* | | X* | | X* | X* | X* | X* |

| | Filter period | Non-myeloablative conditioning | | Short-term follow-up period (monthly) | | | | | | | Medium to long-term follow-up period (every two months) | | | Exit follow-up |
|---|---|---|---|---|---|---|---|---|---|---|---|---|---|---|
| **Visit Point** | Visit 1 | Visit 2 | Visit 3 | Visit 4 | Visit 5 | Visit 6 | Visit 7 | Visit 8 | Visit 9 | Visit 10 | Visit 11 | Visit 12 | Visit 13 | Visit 14 |
| **Visit time window** | -21 | -4 ~ -2 | -1 | 0&2 | 30 ± 5 | 60 ± 5 | 90 ± 5 | 120 ± 5 | 150 ± 5 | 180 ± 5 | 240 ± 5 | 300 ± 5 | 360 ± 5 | at any time |
| **Cardiac assessment [11]** | X | | | | X* | | X* | | X* | | X* | X* | X* | X* |
| **Chest CT or full chest anteroposterior radiograph** | X | | | | X* | | X* | | X* | | X* | X* | X* | X* |
| **Imaging evaluation [12]** | X | | | | X | X | X | X | X | X | X | X | X | X |
| **Bone marrow aspiration/biopsy [13]** | X | | | | X | X | X | X | X | X | X | X | X | X |
| **Lumbar puncture [14]** | X* | | | | X* | X* | X* | X* | X* | X* | | | | X* |
| **Flow cytometry detection of peripheral** | | | | | X | X | X | X | X | X | X | X | X | X |

| | Filter period | Non-myeloablative conditioning | | Short-term follow-up period (monthly) | | | | | | | Medium to long-term follow-up period (every two months) | | | Exit follow-up |
|---|---|---|---|---|---|---|---|---|---|---|---|---|---|---|
| **Visit Point** | Visit 1 | Visit 2 | Visit 3 | Visit 4 | Visit 5 | Visit 6 | Visit 7 | Visit 8 | Visit 9 | Visit 10 | Visit 11 | Visit 12 | Visit 13 | Visit 14 |
| **Visit time window** | -21 | -4 ~ -2 | -1 | 0&2 | 30 ± 5 | 60 ± 5 | 90 ± 5 | 120 ± 5 | 150 ± 5 | 180 ± 5 | 240 ± 5 | 300 ± 5 | 360 ± 5 | at any time |
| **blood CAR-T cells [15]** | | | | | | | | | | | | | | |
| **Flow cytometry detection of peripheral blood lymphocyte subsets [16]** | X | | | X | X | X | X | X | X | X | X | X | X | X |
| **Cytokine Detection [17]** | X | | | X | X | X | X | X | X | X | | | | X |
| **Ferritin [18]** | X | | | X | X | X | X | X | X | X | | | | X |
| **C-reactive protein test [19]** | X | | | X | X | X | X | X | X | X | | | | X |
| **Administer** | | X* | | | | | | | | | | | | |

| | Filter period | Non-myeloablative conditioning | | Short-term follow-up period (monthly) | | | | | | | Medium to long-term follow-up period (every two months) | | | Exit follow-up |
|---|---|---|---|---|---|---|---|---|---|---|---|---|---|---|
| Visit Point | Visit 1 | Visit 2 | Visit 3 | Visit 4 | Visit 5 | Visit 6 | Visit 7 | Visit 8 | Visit 9 | Visit 10 | Visit 11 | Visit 12 | Visit 13 | Visit 14 |
| Visit time window | -21 | -4 ~ -2 | -1 | 0&2 | 30 ± 5 | 60 ± 5 | 90 ± 5 | 120 ± 5 | 150 ± 5 | 180 ± 5 | 240 ± 5 | 300 ± 5 | 360 ± 5 | at any time |
| cyclophosphamide [20] | | | | | | | | | | | | | | |
| Administer fludarabine [21] | | X* | | | | | | | | | | | | |
| Collection of peripheral blood mononuclear cells (PBMCs) [22] | X | | | | | | | | | | | | | |
| reinfusion of CAR-T cells | | | | X | | | | | | | | | | |
| Combined treatment record [23] | X | X | X | X | X | X | X | X | X | X | X | X | X | X |

| | Filter period | Non-myeloablative conditioning | | Short-term follow-up period (monthly) | | | | | | | Medium to long-term follow-up period (every two months) | | | Exit follow-up |
|---|---|---|---|---|---|---|---|---|---|---|---|---|---|---|
| Visit Point | Visit 1 | Visit 2 | Visit 3 | Visit 4 | Visit 5 | Visit 6 | Visit 7 | Visit 8 | Visit 9 | Visit 10 | Visit 11 | Visit 12 | Visit 13 | Visit 14 |
| Visit time window | -21 | -4 ~ -2 | -1 | 0&2 | 30 ± 5 | 60 ± 5 | 90 ± 5 | 120 ± 5 | 150 ± 5 | 180 ± 5 | 240 ± 5 | 300 ± 5 | 360 ± 5 | at any time |
| Drug record [24] | X | X | X | X | X | X | X | X | X | X | X | X | X | X |
| Adverse Event Record | X | X | X | X | X | X | X | X | X | X | X | X | X | X |

*Indicated as an optional examination item, the decision to proceed may be made by the investigator based on the subject's clinical condition.

**Note:**

1. Vital signs assessments should include, at a minimum, measurements of blood pressure, heart rate, respiratory rate, and body temperature. During Visits Day 2 to Day 5, subjects should measure and record axillary temperature and blood pressure (both systolic and diastolic) twice daily, in the morning and evening.
2. Physical examination should include assessment of general condition, skin examination, palpation of superficial lymph nodes (particularly Waldeyer's ring), as well as examination of the liver, spleen, and any abdominal masses.
3. Neurological evaluation: A cranial CT scan may be performed at Visit 1 if clinically indicated. During follow-up, particular attention should be paid to the occurrence of neurotoxic manifestations, including but not limited to aphasia, confusion, delirium, lethargy, and hallucinations. Relevant neurological examinations should be conducted as clinically indicated.
4. Complete blood count (CBC): In addition to the time points specified in the study flowchart, CBC should be reassessed every 1-3 days after CAR-T cell infusion based on the subject's clinical condition. Prior to lumbar puncture, CBC should be performed according to the investigator's clinical judgment.
5. Blood biochemistry tests for evaluation of hepatic and renal function should include, at a minimum: serum lactate dehydrogenase (LDH), β2-microglobulin, total bilirubin, indirect bilirubin, direct bilirubin, creatinine, alanine aminotransferase (ALT), aspartate aminotransferase (AST), cholesterol, blood glucose, and albumin. In addition to the scheduled time points, blood biochemistry should be assessed every 3-5 days within one month after CAR-T cell infusion.
6. CD19 and CD22 testing: For patients with lymphoma or myeloma, CD19/CD22 test results obtained within 3 months prior to enrollment are considered valid and may exempt the subject from testing at Visit 1. Visit 1 is used to confirm CD19/CD22 positivity by immunohistochemistry or flow cytometry. Subsequent visits are used to monitor CD19 and CD22 expression to assess disease recurrence and potential antigen downregulation or loss. In addition to the scheduled time points, CD19 and CD22 expression should be assessed every 3-5 days within one month after CAR-T cell infusion. For CAR-T products involving

immune checkpoint-related evaluation (e.g., PD-1), PD-L1 immunohistochemistry should be performed prior to enrollment. Peripheral blood flow cytometry should be used during follow-up to monitor CD19, CD22, and CAR-T cell PD-1 expression.

7. HBV, HCV, and HIV testing: Test results obtained within 3 months prior to enrollment are considered valid and may exempt the subject from repeat testing.

8. Peripheral blood EBV DNA titer testing: EBV DNA results obtained within 3 months prior to enrollment are considered valid and may exempt the subject from testing at Visit 1. If EBV DNA levels are within the normal range at Visit 1, further testing is not required. If levels are elevated above the normal range, EBV DNA testing should be continued during subsequent visits according to the study flowchart.

9. In vivo lentiviral copy number detection:Quantitative PCR (qPCR) will be used to measure lentiviral copy numbers in vivo. In addition to scheduled assessments, testing should be performed prior to infusion, 20 minutes post-infusion, and every 3-5 days within one month after infusion.

10. Pulmonary function testing is optional and should include, at a minimum, forced expiratory volume in 1 second (FEV1), forced vital capacity (FVC), and diffusing capacity for carbon monoxide (DLCO). Pulmonary function should be carefully evaluated, particularly in subjects with a history of bleomycin exposure.

11. Cardiac assessment: Based on prior treatment history, cardiac function should be comprehensively evaluated, especially in subjects previously treated with anthracyclines (e.g., adriamycin). Assessments include echocardiography, electrocardiogram (ECG), troponin, and NT-pro-BNP, with repeat evaluations performed as clinically indicated.

12. Imaging evaluation: For lymphoma patients, baseline imaging should include the neck, chest, abdomen, and pelvis. Follow-up imaging should be conducted to monitor lesion changes for disease assessment and evaluation of CAR-T therapy efficacy.

13. Bone marrow aspiration/biopsy: Bone marrow aspiration and/or biopsy may be performed for disease diagnosis, assessment of marrow involvement, evaluation of treatment response (e.g., blast percentage), and monitoring of CAR-T cell distribution, density, and persistence. In addition to scheduled assessments, bone marrow aspiration should be performed on Day 15

post-infusion to evaluate minimal residual disease (MRD), CAR-T cell phenotype, and $CD3^+$ T-cell proportion by flow cytometry.

14. Lumbar puncture: Lumbar puncture is optional. At Visit 1, it may be performed to assess central nervous system involvement. Subsequent lumbar punctures may be used to evaluate CNS response and CAR-T cell distribution, density, and persistence. In addition to scheduled assessments, lumbar puncture should be performed on Day 15 post-infusion to assess CAR-T cell density in cerebrospinal fluid by flow cytometry.
15. Peripheral blood CAR-T cell flow cytometry: Flow cytometry of peripheral blood will be used to monitor CAR-T cell phenotype, expansion, persistence, and molecular characteristics. In addition to scheduled assessments, testing should be performed every 3-7 days within one month after infusion.
16. Peripheral blood lymphocyte subset analysis: Flow cytometry will be used to assess lymphocyte subsets and their developmental status in peripheral blood, serving as an indirect indicator of CAR-T cell kinetics and therapeutic efficacy. These results may also guide immunoglobulin replacement therapy.
17. Cytokine testing: Cytokine levels, including but not limited to IL-6, IL-10, IFN-$\gamma$, and TNF-$\alpha$, should be monitored to assess the severity of cytokine release syndrome. In addition to scheduled assessments, cytokines should be measured every 3-5 days within one month after infusion.
18. Ferritin: Serum ferritin levels should be measured to monitor macrophage activation. In addition to scheduled assessments, ferritin should be measured every 3-5 days within one month after infusion.
19. C-reactive protein (CRP): CRP testing should be performed to assess inflammatory status and its correlation with cytokine levels. In addition to scheduled assessments, CRP should be measured every 3-5 days within one month after infusion.
20. Cyclophosphamide administration: Cyclophosphamide should be administered at a dose of 300 mg/m² or 20 mg/kg by intravenous infusion over 60 minutes on Days −4 to −2.
21. Fludarabine administration: Fludarabine should be administered at a dose of 25 mg/m² per day by intravenous infusion over 30 minutes on Days −4 to −2.

**22.** PBMC collection: Peripheral blood mononuclear cells (PBMCs) should be collected for in vitro CAR-T cell manufacturing.

**23.** Concomitant therapy documentation: All concomitant treatments administered during the study, including medications and supportive therapies used for adverse event management, should be documented.

**24.** Prohibited medication records: Use of prohibited medications, including anticancer agents and immunomodulatory drugs, should be recorded throughout the study.

**25.** Waiver of assessments: If cytokine levels, CAR-T cell copy numbers, lentiviral copy numbers, and peripheral blood EBV DNA titers are all below the lower limit of detection at two consecutive visits without confounding treatments (e.g., corticosteroids, tocilizumab), the corresponding assessments may be waived for subsequent visits.

**26.** During the treatment process, investigators may perform any other examinations deemed necessary based on the subject's disease status or adverse events, including but not limited to long-term ECG monitoring, troponin testing, magnetic resonance imaging (MRI), gastroscopy, and colonoscopy.

# I. Research Background

The incidence of malignant hematologic diseases, including leukemia, lymphoma, and multiple myeloma, has been increasing annually and currently ranks among the top ten causes of cancer-related incidence and mortality worldwide, posing a serious threat to public health and socioeconomic development [1-2]. Despite advances in conventional treatments such as chemotherapy, radiotherapy, and hematopoietic stem cell transplantation (HSCT), disease relapse and refractory progression remain the major clinical challenges.

With the development of high-throughput sequencing and other advanced technologies, researchers have gained deeper insights into disease pathogenesis, molecular classification, and individualized therapeutic strategies, thereby improving risk stratification and prognosis prediction. Accumulating evidence from clinical and translational studies indicates that tumor cells evade immune surveillance by downregulating major histocompatibility complex (MHC) molecules, reducing co-stimulatory signals, and hijacking immune checkpoint pathways, ultimately inducing immune tolerance and immune escape. These mechanisms contribute to disease recurrence and treatment resistance. Consequently, therapeutic strategies aimed at restoring immune surveillance and overcoming tumor immune evasion have become essential components of modern anticancer therapy.

**Development of CAR-T Cell Therapy**

In 2013, Science ranked cancer immunotherapy among the top ten scientific breakthroughs of the year [3-4]. Among these, chimeric antigen receptor T-cell (CAR-T) therapy has attracted considerable attention since its first clinical applications. First-generation CAR-T cells were genetically engineered to express tumor-specific single-chain variable fragments (scFvs), enabling MHC-independent recognition and killing of tumor cells. However, the absence of co-stimulatory signaling domains limited their in vivo persistence and antitumor efficacy.

Subsequent second- and third-generation CAR-T cells incorporated co-stimulatory domains such as CD28 and CD137 (4-1BB), which significantly enhanced T-cell activation, proliferation, persistence, and cytotoxicity, thereby overcoming immune evasion caused by reduced co-stimulatory molecule expression

on tumor cells [5]. In 2013, The New England Journal of Medicine reported that two patients with relapsed/refractory acute lymphoblastic leukemia (ALL) achieved complete remission following infusion of anti-CD19 CAR-T cells, highlighting the remarkable clinical potential of this approach [6].

Since then, CAR-T therapies targeting CD19 have demonstrated outstanding efficacy in ALL. In parallel, CAR-T products targeting CD33, CD20, CD30, CD123, and ERBB2 have been developed for use in acute myeloid leukemia, lymphoma, and selected solid tumors [7-11].

**Structure of Chimeric Antigen Receptors**

A chimeric antigen receptor (CAR) is a recombinant antigen receptor that integrates antigen recognition and T-cell activation functions. Structurally, CARs consist of an extracellular domain, a transmembrane domain, and an intracellular signaling domain. Functionally, classical CARs comprise five key components: an scFv derived from monoclonal antibodies that recognizes tumor surface antigens; a hinge region that provides flexibility and facilitates antigen binding; a transmembrane domain that anchors the receptor to the T-cell membrane; a co-stimulatory domain that enhances T-cell activation and survival; and the CD3 ζ signaling domain, which initiates T-cell activation through immunoreceptor tyrosine-based activation motifs (ITAMs).

**Mechanism of Action of CAR-T Therapy**

CAR-T therapy involves the genetic engineering of autologous T cells to express CARs targeting tumor-associated antigens (TAAs). Briefly, CAR constructs encoding scFvs and intracellular signaling domains are generated and introduced into T cells via gene transfer techniques. The modified CAR-T cells are then expanded ex vivo and infused back into the patient at a predefined dose.

Upon antigen recognition, CAR-T cells are activated independently of MHC, leading to downstream signaling cascades that result in T-cell proliferation, cytokine release, and cytotoxic granule secretion, including perforin and granzymes. These processes induce tumor cell apoptosis and mediate potent antitumor effects [12].

**Advantages of CAR-T Therapy**

Compared with conventional T-cell-based immunotherapies, CAR-T therapy offers several advantages [13-17]: CAR-T cells bypass MHC restriction, effectively addressing tumor immune escape caused by MHC downregulation; both protein and

glycolipid antigens can serve as CAR targets, broadening the spectrum of tumor antigens; incorporation of co-stimulatory domains enhances T-cell persistence and resistance to the immunosuppressive tumor microenvironment; CAR constructs can be engineered into other immune effector cells, such as natural killer (NK) cells and cytokine-induced killer (CIK) cells, further expanding therapeutic applications. With continuous technological advancements, CAR-T therapy is expected to play an increasingly important role in cellular immunotherapy for cancer.

**Clinical Procedure of CAR-T Therapy**

The clinical application of CAR-T therapy typically involves five major steps: isolation of peripheral blood T cells or mononuclear cells from the patient; genetic modification of T cells to express tumor-specific CARs; ex vivo expansion of CAR-T cells to the target dose (commonly 1-9 $\times$ $10^5$ -$10^6$ cells/kg); administration of non-myeloablative lymphodepleting chemotherapy prior to CAR-T infusion to reduce tumor burden and immunosuppressive cells; infusion of CAR-T cells followed by efficacy evaluation and intensive safety monitoring.

**Safety Profile of CAR-T Therapy**

Clinical trials have shown that CAR-T therapy is associated with a relatively high incidence of adverse events, although most are manageable with appropriate interventions [18-20]. The most prominent safety concerns include "off-target effects" and cytokine release syndrome (CRS). In sequential anti-CD19 and anti-CD22 CAR-T therapy, off-target effects mainly manifest as B-cell aplasia, which may also serve as a surrogate marker of CAR-T cell persistence and efficacy. CRS is characterized by fever, hypotension, fatigue, confusion, and multi-organ dysfunction and is associated with elevated serum levels of inflammatory cytokines such as IL-6, IFN-$\gamma$, and TNF-$\alpha$. Current CRS management strategies include glucocorticoids and cytokine-targeted therapies. Tocilizumab, an IL-6 receptor antagonist, effectively mitigates CRS without compromising CAR-T antitumor activity. In addition, serum C-reactive protein (CRP), an acute-phase reactant synthesized by the liver, has been widely adopted as a practical biomarker for CRS severity due to its accessibility and cost-effectiveness.

**Risk-Benefit Assessment**

Recombinant CAR-T therapies targeting various tumor antigens have demonstrated significant antitumor efficacy in both preclinical and clinical studies,

particularly anti-CD19 CAR-T therapy in B-cell malignancies. Based on existing evidence, patients with relapsed or refractory disease, those who relapse after standard therapy or HSCT, or those temporarily ineligible for HSCT may derive substantial clinical benefit from sequential anti-CD19/anti-CD22 CAR-T therapy. Although adverse drug reactions are relatively common, most are controllable with timely intervention. Overall, a comprehensive benefit-risk assessment supports that the potential clinical benefits of participation in this study outweigh the associated risks, and enrollment is expected to be beneficial for eligible subjects.

## II. Research Objectives

1. Primary Research Objective:

To evaluate the safety of sequential infusion of anti-CD19 CAR-T and anti-CD22 CAR-T cells in patients with relapsed, refractory, or high-risk B-cell lymphoma.

2. Secondary Research Objectives:

1) To evaluate the clinical efficacy of sequential infusion of anti-CD19 CAR-T and anti-CD22 CAR-T cells in patients with relapsed, refractory, or high-risk B-cell lymphoma.
2) To assess the in vivo expansion and persistence of anti-CD19 CAR-T and anti-CD22 CAR-T cells in treated subjects.

## III. Research Plan

### 3.1 Overview of the Research Plan

This study was designed as a Phase I/II, single-center, open-label, single-arm clinical trial. A total of 500 subjects are planned to be enrolled to evaluate the safety and efficacy of anti-CD19 CAR-T and anti-CD22 CAR-T therapies in patients with relapsed, refractory, or high-risk B-cell lymphoma. The trial consists of seven predefined visit periods (see the visit flowchart for details), as described below:

1) Screening Period (Visit 1: Day −21 to Day −7; duration: approximately 2 weeks). At Visit 1, participants are required to sign the informed consent

form and undergo a series of screening assessments (see Flowchart Visit 1). Based on the screening results, the investigator will determine whether the participant meets all inclusion criteria and does not meet any exclusion criteria. In addition, depending on the participant's clinical condition, the investigator may determine whether certain examination results obtained within one month prior to the informed consent date can be accepted.

2) Non-myeloablative Conditioning Period (Visits 2 – 3: Day −4 to Day −1; duration: 4 days). The necessity and specific regimen of lymphodepleting conditioning will be determined according to the participant's clinical condition, with the fludarabine plus cyclophosphamide (FC) regimen being the most commonly used. After admission to the study center as scheduled by the investigator, participants will receive fludarabine and cyclophosphamide on Days −4 to −2. On Day −1, conditioning treatment will be suspended, and baseline assessments will be performed (see Flowchart Visit 3) to establish reference values prior to CAR-T cell infusion.

3) Short-term Follow-up Period (Visits 4 – 10: Day 0 to Day 180; duration: 6 months). Participants will receive the planned doses of anti-CD19 CAR-T and/or anti-CD22 CAR-T cells on Days 0 and 2 of the trial. During the subsequent 6 months, participants will return to the study center approximately once per month for follow-up assessments (see Flowchart Visits 5 – 10). Investigators will evaluate short-term safety and efficacy based on clinical findings, laboratory results, and other relevant assessments.

4) 5) Medium- to Long-Term Follow-up Period (Visits 11–13: Days 180–360; duration: 6 months): After completion of the short-term follow-up period, subjects return every 2 months for additional assessments over a 6-month period (see Flowchart Visits 11 – 13). During this phase, investigators will continue to evaluate the medium- to long-term safety and efficacy of anti-CD19 CAR-T and anti-CD22 CAR-T therapies.

5) Withdrawal Visit (At Any Time). Participants may withdraw from the study at any time, with or without providing a reason. If a participant withdraws after receiving anti-CD19 CAR-T and/or anti-CD22 CAR-T cell infusion,

the withdrawal procedures specified in the visit flowchart must be completed. If a participant withdraws prior to CAR-T cell infusion, only the required safety assessments need to be performed, and the investigator must document the reason for withdrawal in detail. In cases where treatment is discontinued due to an adverse event (AE), the investigator must conduct appropriate safety follow-up until the AE resolves to baseline levels or stabilizes.

### 3.2 Rationale for the Research Plan

Anti-CD19 CAR-T and anti-CD22 CAR-T therapies are individualized treatments that require the collection of peripheral blood mononuclear cells (PBMCs) from each subject for ex vivo genetic modification and CAR-T cell manufacturing, followed by reinfusion into the same subject. Given the inherent variability in CAR-T cell expansion and in vivo persistence, treatment outcomes cannot be predicted in advance. Therefore, the implementation of a blinded study design is not feasible, and this trial is appropriately designed as an open-label study. Furthermore, as the investigational products in this trial consist of anti-CD19 CAR-T and anti-CD22 CAR-T cells, the primary objectives are to evaluate their safety and efficacy. Considering that CAR-T cell manufacturing processes and technical platforms vary across institutions, and that anti-CD19/anti-CD22 CAR-T therapies are still under active clinical investigation and have not yet reached full technical standardization worldwide, the use of a controlled trial design is not appropriate. Accordingly, this study is designed as a single-center, single-arm clinical trial.

## IV. Study Population

### 4.1 Eligibility Criteria

1. The subject or the subject's legally authorized representative voluntarily agrees to participate in the study and provides written informed consent.
2. Male or female subjects aged 18 to 70 years (inclusive) at the time of enrollment.
3. Subjects with a pathologically and histologically confirmed $CD19^{+}/CD22^{+}$ B-cell

malignancy, who have no effective standard treatment options and have experienced disease relapse following chemotherapy and/or hematopoietic stem cell transplantation (HSCT), or who voluntarily choose sequential infusion of anti-CD19 CAR-T and anti-CD22 CAR-T cells as salvage therapy.

4. Subjects meeting at least one of the following disease conditions:

(1) Residual disease after first-line therapy and unsuitable for autologous or allogeneic HSCT;

(2) Relapse after first complete remission (CR1) and unsuitable for autologous or allogeneic HSCT;

(3) Presence of high-risk disease features;

(4) Disease relapse or lack of remission following HSCT or prior cellular immunotherapy.

5. Presence of measurable or assessable disease lesions according to standard clinical criteria.

6. Adequate function of major organs, defined as:

(1) Hepatic function: ALT and AST < 3 × upper limit of normal (ULN), and total bilirubin ⩽ 34.2 μmol/L;

(2) Renal function: Serum creatinine < 220 μmol/L;

(3) Pulmonary function: Resting oxygen saturation ⩾ 95% on room air;

(4) Cardiac function: Left ventricular ejection fraction (LVEF) ⩾ 40%.

7. Adequate peripheral venous access suitable for intravenous infusion.

8. Eastern Cooperative Oncology Group (ECOG) performance status ⩽ 2, with an estimated life expectancy of at least 3 months.

### 4.2 Exclusion Criteria

1. Female subjects who are pregnant (positive urine or serum pregnancy test) or breastfeeding.
2. Male or female subjects planning to conceive within 1 year after enrollment.
3. Inability or unwillingness to use effective contraception (e.g., condoms, oral contraceptives) for 1 year following enrollment.
4. Presence of uncontrolled infection within 4 weeks prior to enrollment.
5. Active hepatitis B or hepatitis C infection.
6. Known infection with human immunodeficiency virus (HIV).
7. History of severe autoimmune disease or primary immunodeficiency.
8. Known hypersensitivity or allergic predisposition to macromolecular biological agents, such as antibodies or cytokines.
9. Participation in another clinical trial within 6 weeks prior to enrollment.
10. Systemic use of corticosteroids within 4 weeks prior to enrollment (excluding inhaled corticosteroids).
11. History of severe psychiatric illness that may interfere with study participation or compliance.
12. History of drug abuse or substance dependence.
13. Any other condition that, in the opinion of the investigator, would render the subject unsuitable for participation in this study.

### 4.3 Withdrawal Criteria

All participants may withdraw from the study at any time without providing a reason. Withdrawal from the study will not result in any discrimination or retaliation, and participants' entitled medical care and legal rights will not be affected. If a participant who does not meet the inclusion criteria is inadvertently enrolled, the participant shall be withdrawn immediately upon discovery, and the research institution or its designated representative shall be notified promptly. A replacement

participant may be enrolled as appropriate. Unless under exceptional circumstances, if the investigator determines that continued participation in the study may pose a serious or life-threatening risk to the participant's safety, the participant shall be informed and withdrawn from the study. Participants may withdraw from anti-CD19 CAR-T and anti-CD22 CAR-T therapy and/or from the study under any of the following circumstances:

1. During the trial, if a participant experiences intolerable adverse events (AEs) or clinically significant laboratory abnormalities, withdrawal from the study may be initiated based on the investigator's assessment, and appropriate medical measures shall be taken. In such cases, the investigator shall promptly report the event to the research institution or its designated personnel.
2. The participant voluntarily declines to continue participation in the study for any reason.
3. Significant protocol non-compliance that, in the opinion of the investigator, may compromise the integrity of safety or efficacy analyses.
4. Termination of the study by the investigator or the research institution for any reason.

If a participant withdraws from the study after receiving anti-CD19 CAR-T and/or anti-CD22 CAR-T cell infusion, all required exit visit assessments specified in the visit flowchart shall be completed. If a participant withdraws prior to CAR-T cell infusion, only the required safety-related assessments need to be completed, and the investigator must document the reason(s) for withdrawal in detail. Participants who withdraw from the study early do not require replacement enrollment unless otherwise specified.

**4.4 Suspension Criteria**

1. Occurrence of serious safety issues during the trial that may pose unacceptable risks to participants.
2. Request for study suspension by the study site or research institution.

3. Identification of significant errors or deficiencies in the study protocol that compromise the ability to adequately evaluate the safety and efficacy of the investigational therapy.
4. Suspension requested by regulatory authorities or administrative bodies.

## V. Treatment Protocol

### 5.1 Patient Screening; Cell Collection, Modification, and Expansion

(1) Subjects shall be admitted to the hospital approximately 3 weeks prior to CAR-T cell infusion and provide written informed consent. A series of screening assessments will then be completed (see Flowchart Visit 1). Based on the screening results and the predefined inclusion and exclusion criteria, the investigator will determine whether the subject is eligible for enrollment.

(2) For eligible subjects, peripheral blood will be collected by venipuncture or peripheral blood mononuclear cells (PBMCs) will be obtained by leukapheresis on Day −10. The collected peripheral blood or PBMCs will be transported to the central laboratory under controlled conditions at 4° C.

(3) Genetic modification, activation, and ex vivo expansion of CAR-T cells typically require approximately 7 – 10 days. The central laboratory will estimate the time required for CAR-T cell expansion to reach the planned infusion dose based on culture conditions. The investigator will determine whether non-myeloablative lymphodepleting conditioning is required based on the subject's clinical status.

### 5.2 Non-myeloablative Conditioning

(1) Lymphodepleting preconditioning will be initiated on Day −4, followed by three consecutive days of fludarabine and cyclophosphamide administration (Days −4 to −2).

(2) Study assessments specified in Flowchart Visits 2 and 3 will be completed before and after completion of non-myeloablative conditioning to document

the subject's baseline status and response to the conditioning regimen.

### 5.3 CAR-T Cell Infusion

CAR-T cells will be administered intravenously on Day 0 using a standard infusion set, with supplemental oxygen provided as needed. Continuous monitoring, including electrocardiography, blood pressure, and oxygen saturation, will be performed throughout the infusion. The infusion will be completed over approximately 30 minutes, followed by flushing of the infusion line with normal saline.

Note: The infusion of immune checkpoint-modified CAR-T cells and the administered dose may be adjusted based on the subject's clinical condition and investigator judgment.

### 5.4 Adverse Reactions and Management

During CAR-T cell infusion and the post-infusion follow-up period, subjects will be closely monitored for adverse reactions. Emergency equipment and medications must be readily available during hospitalization. In the event of adverse reactions, prompt symptomatic and supportive treatment shall be initiated. Recommended management measures are described below:

(1) Cytokine Release Syndrome (CRS):

**Table 1. Classification criteria for CRS severity:**

| | |
|---|---|
| **Grade 1** | Mild reactions may occur, including fever, nausea, vomiting, fatigue, headache, myalgia, and irritability. Symptoms can be managed with supportive care alone, such as antihistamines, nonsteroidal anti-inflammatory drugs (NSAIDs), and sedatives. |
| **Grade 2** | Hypotension and/or exertional dyspnea that resolves rapidly (within 24 hours) after moderate-intensity intervention. Management includes low-flow oxygen supplementation ($FiO_2$ <40%), adequate intravenous fluid resuscitation, or the use of a single vasoactive |

| | |
|---|---|
| | agent. May be accompanied by Grade 2 organ toxicity. |
| **Grade 3** | Severe CRS presenting with one or more of the following manifestations and requiring urgent medical intervention due to critical condition:<br>• A decrease in systolic, diastolic, or mean arterial pressure >20% from baseline that is unresponsive to sustained rapid intravenous fluid resuscitation (>20 mL/kg), or requiring multiple or high-dose vasoactive agents;<br>• Grade 3 respiratory failure, including dyspnea at rest, inability to perform self-care, $SpO_2$ <88% or $PaO_2$ <55 mmHg while receiving oxygen with $FiO_2$ ≥40%;<br>• Grade 3 organ toxicity (e.g., heart failure) or Grade 4 hypertransaminemia (>20 × ULN). |
| **Grade 4** | Life-threatening CRS requiring mechanical ventilation, or associated with Grade 4 organ toxicity (excluding isolated hypertransaminemia). |
| **Grade 5** | Death. |

Note: The organ toxicity grading is based on the CTCAE v4.0 grading system.

For patients with suspected severe CRS (grade 3-4), close monitoring of the condition is required, and other possible causes (such as septic shock) should be actively ruled out. If necessary, transfer to the intensive care unit (ICU) is recommended. Treatment should include immunosuppressants, with tocilizumab, an IL-6 receptor antagonist, as the first-line therapy.

(2) B lymphocyte depletion: Intravenous immunoglobulin (IVIG) may be administered as supportive therapy to maintain serum IgG levels and reduce the risk of infection.

(3) Neurotoxicity: Clinical manifestations may include drowsiness, lethargy, stupor, and, in severe cases, seizures. These symptoms are often self-limiting and can be managed with appropriate symptomatic and supportive treatment.

(4) Fever: Management includes physical cooling measures or administration of nonsteroidal anti-inflammatory drugs (NSAIDs). Corticosteroids should be avoided whenever possible unless clinically indicated.

(5) Myalgia: Symptomatic treatment with conventional nonsteroidal anti-inflammatory drugs (NSAIDs) is recommended.

(6) Hypotension: Intravenous fluid resuscitation should be initiated promptly, and vasoactive agents may be administered as needed to maintain adequate blood pressure.

(7) Respiratory distress or respiratory failure: Management includes infection prevention, maintenance of airway patency, and oxygen supplementation. If necessary, non-invasive ventilation (BiPAP) or invasive mechanical ventilation with endotracheal intubation should be performed.

(8) Leukopenia: To prevent and treat infections, empirical broad-spectrum antibiotics may be administered, accompanied by active etiological investigations as clinically indicated.

(9) Coagulation disorders: Coagulation abnormalities may present as prolonged prothrombin time (PT), activated partial thromboplastin time (APTT), or decreased fibrinogen levels, and should be managed with appropriate supportive and symptomatic treatment.

### 5.5 Monitoring and Follow-up

Patients are required to complete Visits 2 through 5 during hospitalization, except for those who withdraw prematurely from the study. The investigator will determine patient eligibility for discharge based on the findings at Visit 5 and the resolution or stabilization of adverse events. Post-discharge follow-up is conducted as follows:

(1) short-term follow-up period

Within 6 months after CAR-T cell infusion, patients are scheduled for monthly hospital visits (Visits 5 – 10, see Flowchart) to monitor and evaluate the short-term

safety and efficacy of CAR-T therapy.

(2) Medium to long-term follow-up period

Within 6 months following Visit 9, patients will return for follow-up every 2 months (Visits 11 – 13, see Flowchart) to assess the medium- and long-term safety and efficacy of CAR-T therapy.

(3) Long-term follow-up period

After completion of Visit 13, patients will be followed up by telephone every 4 months until disease progression, death, or loss to follow-up. Detailed records shall be maintained regarding previously reported adverse events, disease status, and any subsequent treatments.

## VI. Concomitant Therapy and Prohibited Medications

During the 1-year treatment and follow-up period, concomitant therapies may be adjusted if the patient's condition deteriorates or disease progression occurs, or if the investigator determines that such adjustments may improve clinical outcomes.

## VII. Trial Design and Blinding

This study was designed as an open-label trial. As the trial involved individualized treatment, peripheral mononuclear cells (PMCs) from participants were collected, genetically modified to prepare anti-CD19 CAR-T and anti-CD22 CAR-T cells, and reinfused into the participants at the intended dose after passing multiple quality control tests. Therefore, a blinded approach was not feasible, and the trial was designed as an open-label trial.

## VIII. Treatment Compliance

Participant compliance is promoted through the following measures:

1. Investigators shall rigorously implement the informed consent process to ensure

that participants fully understand the study objectives, procedures, and requirements prior to enrollment.

2. Costs associated with CAR-T cell preparation — including cell collection, purification, genetic modification, activation, expansion, and quality control— are waived for study participants.
3. During the study, no changes to the infusion timing, dose, volume, or route (intravenous administration) of anti-CD19 or anti-CD22 CAR-T cells may be made without prior written approval from the investigator. All protocol deviations must be fully documented.
4. Investigators shall conduct scheduled study visits in a timely manner, arrange all required assessments, promptly address participant inquiries, and ensure immediate management and documentation of any adverse events.

## IX. Evaluation Endpoints and Criteria

### 9.1 Primary End Points

Safety outcomes associated with anti-CD19 CAR-T and/or anti-CD22 CAR-T therapy, including the incidence and severity of adverse events (AEs), serious adverse events (SAEs), and clinically significant laboratory abnormalities.

### 9.2 Secondary End Points

1. Objective response rates (ORR) of tumors at 3 months, 6 months, and 1 year post-treatment follow-up: complete response (CR) and partial response (PR);
2. overall survival (OS);
3. progression-free survival (PFS);
4. event-free survival (EFS);
5. Kinetics of in vivo expansion of anti-CD19 CAR-T and anti-CD22 CAR-T cells in subjects, assessed in peripheral blood, bone marrow, cerebrospinal fluid, lymph nodes, and other relevant compartments;

6. Duration of persistence of anti-CD19 CAR-T and anti-CD22 CAR-T cells in vivo;
7. Characteristics and extent of lymphocyte depletion in treated subjects.

**9.3 Safety Assessments**

9.3.1 Definition of Adverse Events

The investigator is responsible for documenting all adverse events occurring during the study and for evaluating the onset, duration, severity, causality, management, and outcomes of each AE in relation to anti-CD19 and/or anti-CD22 CAR-T cell therapy.

Adverse Event (AE):

An AE is defined as any unfavorable and unintended medical occurrence in a subject receiving anti-CD19 and/or anti-CD22 CAR-T cell therapy, regardless of whether it is considered related to the investigational product. An AE may include any abnormal sign (including abnormal laboratory findings), symptom, or disease temporally associated with the use of the investigational therapy.

Serious Adverse Event (SAE):

An SAE refers to any adverse medical occurrence that occurs after administration of any dose of anti-CD19 and/or anti-CD22 CAR-T cells and meets one or more of the following criteria:

- Results in death;
- Is life-threatening (i.e., the subject was at immediate risk of death at the time of the event);
- Requires inpatient hospitalization or prolongation of existing hospitalization (excluding planned hospitalizations not related to disease worsening);
- Results in persistent or significant disability or incapacity;
- Results in a congenital anomaly or birth defect;

- Other medically significant events that may not immediately result in death or hospitalization but require medical or surgical intervention to prevent serious outcomes.

Adverse Drug Reaction (ADR): Any adverse and unintended reaction related to anti-CD19 and/or anti-CD22 CAR-T cell therapy occurring after administration of the investigational product.

Unexpected Adverse Reaction: An adverse reaction whose nature, severity, or frequency is not consistent with the known safety information of anti-CD19 and/or anti-CD22 CAR-T cell therapy.

9.3.2 Severity of adverse events

All adverse events and adverse drug reactions shall be graded according to the National Cancer Institute Common Terminology Criteria for Adverse Events (NCI-CTCAE), Version 4.0.

9.3.3 Relationship between Adverse Events and Study Drug Anti-CD19 CAR-T/anti-CD22 CAR-T Cells

The relationship between anti-CD19 CAR-T/anti-CD22 CAR-T cell therapy and AE was categorized into five types: unrelated, possibly unrelated, possibly related, related, and uncertain (unable to determine).

- If there is no reasonable possibility of association between the adverse event (AE) and the investigational drug anti-CD19 CAR-T/anti-CD22 CAR-T cell therapy, the AE is considered to be unrelated to the investigational drug.
- Non-relevance may refer to clinical events that are not fundamentally or completely related to the investigational drug anti-CD19 CAR-T/anti-CD22 CAR-T cell therapy, but can be explained by other conditions, including chronic diseases, other concomitant drug reactions, or disease progression.
- Potentially relevant refers to clinical events with an unknown association between the adverse event (AE) and the investigational drug anti-CD19 CAR-T/anti-CD22 CAR-T cell therapy, but which cannot be explained by other causes.

- Potential relevance refers to adverse events (AEs) that exhibit a temporal relationship with the investigational drug anti-CD19 CAR-T/anti-CD22 CAR-T cell therapy, and which investigators determine cannot be explained by other causes (complications, disease progression, or concomitant medications).
- Adverse events (AEs) should be classified according to the above categories. An AE may be classified as 'uncertain' (cannot be determined) only when the causal relationship cannot be assessed, for example, due to insufficient evidence, conflicting evidence, conflicting data, or poor documentation quality.

9.3.4 Collection, Documentation, and Reporting of AE

All AEs occurring during the study, regardless of severity or causality, must be collected and recorded in the adverse event section of the Case Report Form (CRF). Required documentation includes the AE description, onset and resolution dates, severity, actions taken, outcome, and relationship to the investigational product. Each AE shall be recorded as a separate entry.

9.3.5 Reporting of Serious Adverse Events

All SAEs occurring during the study must be reported in accordance with applicable regulatory and ethics committee requirements, regardless of causality.

Investigators shall:

- Initiate appropriate medical management immediately;
- Report the SAE to the study sponsor and the designated safety officer within 24 hours of awareness;
- Record the SAE in the CRF AE and SAE report forms and source documents;
- Submit a signed and dated SAE report to the independent ethics committee within 24 hours;
- Follow the SAE until resolution, stabilization, or return to baseline.

9.3.6 Management and Follow-up of Adverse Events

Investigators are responsible for providing appropriate medical care for all AEs. All AEs observed from screening through study completion shall be followed until resolution, stabilization, or return to baseline.

For trial-related adverse events, all treatment and compensation costs shall be borne by the CAR-T cell preparation unit, Wuhan Boruida Biotechnology Co., Ltd.

9.3.6.1 Clinical Laboratory Evaluations

Investigators shall review laboratory results promptly to assess normality, trends, and clinical significance. Clinically significant abnormal laboratory findings shall be recorded as laboratory AEs. Abnormal laboratory values attributable to the subject's underlying disease shall be documented but not reported as AEs unless clinically significant.

9.3.6.2 Vital Signs, Physical Examination, and Other Safety Assessments

Based on previous clinical research experience, a series of toxic reactions of varying degrees may occur in subjects after CAR-T cell infusion, with fever, hypotension, and mental status changes being common, while respiratory and cardiac toxicity are more severe. Therefore, researchers must pay sufficient attention and evaluate the following test results:

Vital signs assessments: includes blood pressure, heart rate, respiratory rate, and body temperature. During Visits 2 to 5, subjects should measure and record axillary temperature and blood pressure (both systolic and diastolic) twice daily, in the morning and evening.

Physical examination: includes general condition, skin examination, palpation of superficial lymph nodes (especially Weil's ring), liver, spleen, and abdominal masses.

Neurological assessment: Close monitoring for neurotoxicity, including aphasia, confusion, mania, seizures, lethargy, and hallucinations.

# X. Data Management

## 10.1 Case Report Filling and Handover

The case report form is completed by the investigator, and each enrolled case must submit a case report form. The completed case report form is reviewed by the clinical monitor, and the first copy is transferred to the data administrator for data entry and management.

## 10.2 Data Entry and Modification

Data entry and management shall be conducted by a professional data management unit. Data administrators shall use data management software to develop data entry procedures for data entry and management. To ensure data accuracy, two data administrators shall independently perform duplicate entry and cross-check the data.

For questions raised in the case report form, the data administrator will generate a Data Request Questionnaire (DRQ) and submit it to the investigator via the clinical monitor. The investigator is required to respond promptly. Based on the investigator's responses, the data administrator will modify, confirm, and enter the data. If necessary, the DRQ may be resubmitted.

## 10.3 Data Review and Locking

Upon completion of data management, the primary investigator, data administrator, and statistical analyst jointly confirm the statistical analysis population. After resolving all data-related questions, the database is locked and the review report is signed simultaneously.

# XI. Statistical Analysis

Upon completion of all study assessments and after the database has been cleared and closed, a final analysis will be conducted for the entire study population at the end of the study. The statistical analysis plan, which will include more detailed information about the analysis, will be drafted and finalized prior to the database closure.

## 11.1 Determination of Sample Size

This study enrolled 500 subjects based on practical considerations and the minimum regulatory requirements, with the number of potential participants meeting informed consent.

## 11.2 Statistical Analysis Population

Intent-to-Treat (ITT) population: All subjects who received anti-CD19 CAR-T/anti-CD22 CAR-T cell therapy.

## 11.3 Statistical Analysis Plan

11.3.1 General Principles of Statistical Analysis

Statistical analyses in this study will be performed using SAS 9.2 or later versions. Unless otherwise specified, all hypothesis tests will be conducted as two-tailed tests, with a significance level of $p < 0.05$. P-values will be rounded to four decimal places, and 95% confidence intervals will be calculated when necessary for $p < 0.0001$. Descriptive analysis for single groups will be conducted as follows: continuous variables will be summarized as frequency, mean, standard deviation, median, first quartile, third quartile, minimum, and maximum; categorical variables will be summarized as frequency and percentage.

11.3.2 Statistical Analysis Methods

1) Methods for Deletion and Missing Data Processing

The analysis of secondary endpoints will include data from participants who were dropped out. When using the ITT set for secondary endpoint analysis, secondary endpoints for which the complete treatment course was not observed will be carried forward to the final trial results using the Last Observation Carried Forward (LOCF) method. LOCF will be the primary method for filling in missing data.

2) interim analysis

This study will conduct two interim analyses. The first analysis will be performed when 100% of the participants complete the short-term follow-up period (0-180 days, duration of 6 months), and the second analysis will be performed when 100% of the participants complete the medium-to-long-term follow-up period (180-360 days, duration of 6 months).

3) Multicenter study

not applicable.

4) Multiple Comparisons and Adjustments for Multiplicity

not applicable.

5) Subgroup analysis

not applicable.

11.3.3 Statistical Analysis

1) fall off analysis

The total number of subjects who were enrolled and excluded were classified according to the description.

Summarize the subject information by treatment group and primary reason (e.g., loss to follow-up, adverse events [AEs], poor compliance), including the number of subjects enrolled and those who entered or completed each study phase (or week/month), as well as all reasons for discontinuation after enrollment. Elucidate whether subjects who discontinued anti-CD19 CAR-T/anti-CD22 CAR-T cell therapy continued to be followed during the study period.

Provide a list of all subjects who discontinued the study after enrollment, including subject number, specific reason for discontinuation, treatment (anti-CD19

CAR-T/anti-CD22 CAR-T cells and dose), cumulative dose (if applicable), and duration of treatment prior to discontinuation.

2) Demographics and other baseline characteristics

Based on the numerical characteristics of variables, continuous variables (e.g., age, height, weight, etc.) will be summarized using case counts, mean, standard deviation, median, first quartile, third quartile, minimum, and maximum values; categorical variables (e.g., gender, medical history, prior treatment, etc.) will be summarized using case counts and percentages.

3) Analysis of Compliance and Drug Exposure

not applicable.

4) efficiency analysis

For ORR analysis, the results were described according to different follow-up time points after treatment (3 months, 6 months, and 1 year).

The Kaplan-Meier method and survival curves will be used to describe the analysis of OS, PFS, and EFS.

The analysis of the expansion level, distribution characteristics, and persistence duration of anti-CD19 CAR-T/anti-CD22 CAR-T cells in subjects should be described based on different follow-up time points post-treatment and various tissues in vivo (peripheral blood, bone marrow, cerebrospinal fluid, lymph nodes, etc.), with corresponding characteristics and follow-up time points plotted as line graphs.

Based on the characteristics of lymphocyte depletion and in vivo replication of retroviruses, descriptions should be provided according to different follow-up time points, and line graphs should be plotted using the aforementioned characteristics and follow-up time points.

5) safety analysis

Safety was evaluated by summarizing changes in AE, laboratory test results, and vital signs. Any subject who had received at least one treatment with anti-CD19 CAR-T or anti-CD22 CAR-T cells was included in the safety analysis.

a) adverse event

Safety was evaluated by summarizing changes in AE, laboratory test results, and vital signs. Any subject who had received at least one treatment with anti-CD19 CAR-T or anti-CD22 CAR-T cells was included in the safety analysis.

b) laboratory examination

Descriptive statistics (counts and percentages) of all laboratory test results and changes compared to baseline.

c) ECG check up

not applicable.

d) Vital signs, physical examination, and other safety-related assessments

Descriptive statistics (counts and percentages) of vital signs and changes from baseline.

The analysis and presentation of vital signs, other physical examination findings, and other safety-related observations should follow methods similar to those used for laboratory variables.

e) Other safety analyses

not applicable.

## XII. Quality Assurance and Quality Control

According to the guidance principles of China Good Clinical Practice (GCP), research institutions are responsible for implementing and maintaining quality assurance and quality control systems in accordance with the corresponding standard operating procedures (SOPs) to ensure that the conduct of clinical trials and the collection, recording, and reporting of data comply with the protocol, GCP, and relevant regulatory requirements. To ensure the reliability and proper handling of data, quality control should be performed at each stage of data processing. In addition, the CRO Clinical Quality Assurance (CQA) department may conduct regular audits of the research process, covering but not limited to research centers, center visits, center laboratories, suppliers, clinical databases, and final clinical research reports. Regulatory authorities may also conduct inspections during the research process or at

any time after the study concludes.

During the course of this study, assigned clinical monitors conducted regular on-site monitoring visits to the research center to ensure strict adherence to all aspects of the study protocol and accurate completion of study data.

1) Participants must possess the professional expertise, qualifications, and capabilities required to undertake this clinical study, and undergo standardized training with uniform documentation methods and evaluation criteria.
2) The entire clinical research process shall be strictly conducted in accordance with this study protocol.
3) Researchers should accurately, thoroughly, and meticulously record all content in the Case Report Form (CRF) in accordance with the CRF completion requirements to ensure the authenticity and reliability of the CRF content.
4) All examinations were conducted using standardized methods and evaluation criteria. The abnormality assessment for laboratory tests was based on the reference ranges established by the respective testing institutions.
5) All observations and findings in clinical studies should be validated to ensure data reliability and confirm that conclusions are derived from the original data. Appropriate data management measures are implemented during both the clinical study and data processing phases.
6) Designate monitors to conduct regular on-site inspections of clinical trial sites to ensure strict adherence to the study protocol and accurate, reliable completion of study data.
7) The drug regulatory authority entrusts inspectors not directly involved in the clinical trial to conduct systematic inspections of clinical trial-related activities and documentation, to evaluate whether the trial was conducted in accordance with the protocol, standard operating procedures, and relevant regulatory requirements, and whether trial data were recorded in a timely, truthful, accurate, and complete manner.
8) Researchers should make every effort to minimize subject withdrawals during

treatment, keeping the case dropout rate below 20%. For the benefit of the subjects, those who must withdraw from the study should still undergo follow-up to facilitate analysis. For withdrawn subjects, all examinations and evaluations for early study termination should be completed as much as possible.

## XIII. Ethical Requirements and Informed Consent of Subjects

1. Ethics Committee

The research institution or its designated personnel shall prepare the relevant documents to be submitted to the Ethics Committee (EC) of the research center. The trial protocol, informed consent forms, subject recruitment materials or advertisements (if applicable), and other documents required by regulations must be submitted to the corresponding EC for approval. Prior to initiating this study, written approval from the EC must be obtained. The EC's approval document must clearly specify the name, number, version number of the study protocol, and the version number of other documents (e.g., informed consent forms) as well as the approval date.

The research center must comply with the requirements of the Ethics Committee (EC). When revising the protocol, informed consent form, or subject recruitment materials, the EC must be submitted for approval. In accordance with the safety reporting requirements of the center's location and the EC's regulations, regular reports and updates must be submitted, along with the final version of the report. All the aforementioned documents and EC approvals must be submitted to the research unit or designated personnel.

2. Ethical Implementation of This Study

The research process and the acquisition of informed consent shall comply with the Declaration of Helsinki, China GCP requirements, and relevant national laws and regulations to protect the rights, safety, and health of participants. Prior to the initiation of the trial, the protocol must be reviewed by the hospital ethics committee

of the responsible research unit, and approval documents must be issued before the trial protocol can be implemented.

3. Subject Information and Informed Consent

Prior to the commencement of the study, the investigator shall use an Informed Consent Form (ICF) to explain the potential risks and benefits of the study to eligible participants, with the language of the informed consent being simple and understandable. The ICF statement shall clearly state that the informed consent is voluntary, that participation in the study may entail certain risks and benefits, and that participants may withdraw from the study at any time. The investigator may only enroll a participant after providing a detailed explanation of the study protocol and obtaining written consent from the participant or their legal representative. The ICF shall be signed with a date and retained by the participant as a copy of the informed consent form. Regulations regarding the storage and ownership of clinical research data.

## XIV. Provisions on the Preservation and Ownership of Clinical Research Data

To ensure the evaluation and supervision by the National Medical Products Administration (NMPA), the investigators shall agree to retain all study materials, including original data of all enrolled subjects, all signed informed consent forms, and detailed records of all Case Report Forms (CRFs). The retention period shall be at least 5 years.

All rights to the clinical study data belong to Tongji Hospital affiliated with Tongji Medical College of Huazhong University of Science and Technology. Except for the National Medical Products Administration (NMPA), the investigators shall not provide the data to any third party in any form without the written consent of the research institution.

## XV. References


1. World Health Organization et al. *World Cancer Report 2014*. Chapter 5.13 (WHO, 2014).
2. Swerdlow, S. H. et al. *WHO Classification of Tumours of Haematopoietic and Lymphoid Tissues*, 4th edn (IARC, 2008).
3. Kenderian, S. S. et al. Chimeric antigen receptor T-cell therapy to target hematologic malignancies. *Cancer Res.* **74**, 6383–6389 (2014).
4. Pico de Coaña, Y. et al. Checkpoint blockade for cancer therapy: revitalizing a suppressed immune system. *Trends Mol. Med.* **21**, 482–491 (2015).
5. Long, A. H. et al. 4-1BB costimulation ameliorates T cell exhaustion induced by tonic signaling of chimeric antigen receptors. *Nat. Med.* **21**, 581–590 (2015).
6. Grupp, S. A. et al. Chimeric antigen receptor–modified T cells for acute lymphoid leukemia. *N. Engl. J. Med.* **368**, 1509–1518 (2013).
7. Kakarla, S. et al. CAR T cells for solid tumors: armed and ready to go? *Cancer J.* **20**, 151–155 (2014).
8. Gilham, D. E. et al. CAR-T cells and solid tumors: tuning T cells to challenge an inveterate foe. *Trends Mol. Med.* **18**, 377–384 (2012).
9. Curran, K. J. et al. Enhancing anti-tumor efficacy of chimeric antigen receptor T cells through constitutive CD40L expression. *Mol. Ther.* **23**, 769–778 (2015).
10. Batlevi, C. L. et al. Novel immunotherapies in lymphoid malignancies. *Nat. Rev. Clin. Oncol.* **13**, 25–40 (2016).
11. Klaver, Y. et al. Adoptive T-cell therapy: a need for standard immune monitoring. *Immunotherapy* **7**, 513–533 (2015).
12. Brentjens, R. J. et al. Safety and persistence of adoptively transferred autologous CD19-targeted T cells in patients with relapsed or chemotherapy-refractory B-cell leukemias. *Blood* **118**, 4817–4828 (2011).
13. Vesely, M. D. et al. Natural innate and adaptive immunity to cancer. *Annu. Rev. Immunol.* **29**, 235–271 (2011).

14. Pule, M. A. et al. Virus-specific T cells engineered to coexpress tumor-specific receptors: persistence and antitumor activity in individuals with neuroblastoma. *Nat. Med.* **14**, 1264–1270 (2008).

15. Dotti, G. et al. Fifteen years of gene therapy based on chimeric antigen receptors: are we nearly there yet? *Hum. Gene Ther.* **20**, 1229–1239 (2009).

16. Park, T. S. et al. Treating cancer with genetically engineered T cells. *Trends Biotechnol.* **29**, 550–557 (2011).

17. Kalos, M. et al. T cells with chimeric antigen receptors have potent antitumor effects and can establish memory in patients with advanced leukemia. *Sci. Transl. Med.* **3**, 95ra73 (2011).

18. Davila, M. L. et al. Efficacy and toxicity management of 19–28z CAR T cell therapy in B cell acute lymphoblastic leukemia. *Sci. Transl. Med.* **6**, 224ra25 (2014).

19. Lee, D. W. et al. How I treat: current concepts in the diagnosis and management of cytokine release syndrome. *Blood* **124**, 188–195 (2014).

20. Maus, M. V. et al. Antibody-modified T cells: CARs take the front seat for hematologic malignancies. *Blood* **123**, 2625–2635 (2014).

# Protocol

## An open-label, single center, single arm clinical study for the sequential infusion of anti-CD19 CAR-T and anti-CD22 CAR-T therapy following autologous hematopoietic stem cell transplantation (ASCT) for relapsed, refractory, and high-risk B cell lymphoma

**Registration number:** ChiCTR-OPN-16009847

**PI/Department:** Professor Jianfeng Zhou /Department of Hematology.

**Study sponsor and monitor:** Tongji Hospital, Tongji Medical College, Huazhong University of Science and Technology

**Technical Partners:** Wuhan Bioraid Co., Ltd.

### declaration of secrecy

This protocol is classified as a confidential document. All information contained herein is the property of the research institution (Tongji Hospital Affiliated to Tongji Medical College, Huazhong University of Science and Technology) and is provided exclusively for review by the investigators, collaborating researchers, the ethics committee, and relevant regulatory authorities. No dissemination or disclosure is permitted without prior written approval from the research institution.

# catalogue

Abstract ........................................ 3
Treatment Flowchart ........................................ 8
I. Research Background ........................................ 23
II. Research Objectives ........................................ 26
III. Research Plan ........................................ 27
3.1 Overview of the Research Plan ........................................ 27
3.2 Rationale for the Research Plan ........................................ 28
IV. Study Population ........................................ 28
4.1 Eligibility Criteria ........................................ 28
4.2 Exclusion Criteria ........................................ 30
4.3 Withdrawal Criteria ........................................ 30
4.4 Suspension Criteria ........................................ 31
V. Treatment Protocol ........................................ 31
5.1 Patient Screening; Cell Collection, Modification, and Expansion ........................................ 31
5.2 Autologous Hematopoietic Stem Cell Transplantation ........................................ 32
5.3 CAR-T Cell Infusion ........................................ 32
5.4 Adverse Reactions and Management ........................................ 33
5.5 Monitoring and Follow-up ........................................ 35
VI. Concomitant Therapy and Prohibited Medications ........................................ 36
VII. Trial Design and Blinding ........................................ 36
VIII. Treatment Compliance ........................................ 36
IX. Evaluation Endpoints and Criteria ........................................ 37
9.1 Primary End Points ........................................ 37
9.2 Secondary End Points ........................................ 37
9.3 Safety Assessments ........................................ 37
X. Data Management ........................................ 41
10.1 Case Report Filling and Handover ........................................ 41
10.2 Data Entry and Modification ........................................ 41
10.3 Data Review and Locking ........................................ 42
XI. Statistical Analysis ........................................ 42
11.1 Determination of Sample Size ........................................ 42
11.2 Statistical Analysis Population ........................................ 42
11.3 Statistical Analysis Plan ........................................ 43
XII. Quality Assurance and Quality Control ........................................ 46
XIII. Ethical Requirements and Informed Consent of Subjects ........................................ 47
XIV. Provisions on the Preservation and Ownership of Clinical Research Data ........................................ 49
XV. References ........................................ 50

# Abstract

| | |
|---|---|
| **research topic** | An open-label, single center, single arm clinical study for the sequential infusion of anti-CD19 CAR-T and anti-CD22 CAR-T therapy following autologous hematopoietic stem cell transplantation (ASCT) for relapsed, refractory, and high-risk B cell lymphoma |
| **purpose of research** | Primary research objectives:<br>To evaluate the safety of sequential infusion of anti-CD19 CAR-T and anti-CD22 CAR-T therapy after autologous hematopoietic stem cell transplantation for relapsed/refractory/high-risk B-cell lymphoma.<br>Secondary research objectives:<br>1. To evaluate the clinical efficacy of sequential infusion of anti-CD19 CAR-T and anti-CD22 CAR-T therapies following autologous hematopoietic stem cell transplantation in the treatment of relapsed/refractory/high-risk B-cell lymphoma;<br>2. To evaluate the expansion effect and persistence of anti-CD19 CAR-T/anti-CD22 CAR-T cells in recipients after autologous hematopoietic stem cell transplantation. |
| **research design** | An open-label, single-center, single-arm clinical study |
| **Subject** | 150 cases of B-cell malignancies patients. |
| **trial period** | including screening, treatment and follow-up.<br>The screening and enrollment phase lasted 1 week.<br>The observation period lasted until 6 months after reinfusion (short-term follow-up).<br>The medium-and long-term follow-up is expected to be 1 year. |
| **Inclusion criteria** | Participants must meet all of the following:<br>(1) Signed informed consent obtained before any study procedures.<br>(2) Age ≥ 18 years.<br>(3) Pathologically confirmed $CD19^{+}/CD22^{+}$ B-ALL or B-NHL. |

| | |
|---|---|
| | (4) Relapsed or refractory B-cell malignancy, including: B-ALL; Aggressive B-cell lymphomas: DLBCL, BL, MCL, or transformed B-cell lymphoma.<br>(5) At least one of the following:<br>a) Failed ≥2 lines of salvage therapy<br>b) Relapsed/refractory post-HSCT.<br>c) High-risk features (e.g., double -hit lymphoma)<br>d) Ineligible for allo-HSCT (B-ALL)<br>(6) At least one measurable lesion.<br>(7) Adequate organ function, defined as creatinine<2.5mg/dl; aspartate transaminase/ alanine transaminase <3 × upper limit of normal; SiO2 ≥ 95%; bilirubin < 2.0 mg/dl; LVEF > 40%.<br>(8) Sufficient venous access for leukapheresis.<br>(9) ECOG performance status ≤ 2.<br>(10) Estimated survival of ≥ 3 months. |
| **exclusion criteria** | Any of the following will lead to exclusion:<br>(1) Pregnant or lactating women, or planning pregnancy within 1 year<br>(2) Active HBV, HCV, or HIV infection.<br>(3) Uncontrolled systemic infections.<br>(4) Systemic steroid use within 4 weeks.<br>(5) Known allergy to cytokines or antibodies.<br>(6) Participation in other clinical trials within 6 weeks.<br>(7) Active graft-versus-host disease. |

| | |
|---|---|
| | (8) History of psychiatric disorders or another primary malignancy<br>(9) Substance abuse or addiction.<br>(10) Other conditions deemed unsuitable by the investigator. |
| **Test product** | Test products: anti-CD19 CAR-T cells; anti-CD22 CAR-T cells;<br>Reinfusion dose: The investigator determines the reinfusion dose based on the subject's individual/disease condition and in vitro preparation status.<br>Administration route: Intravenous infusion/push for 30 minutes. |
| **stages of research** | 1. Screening period (Visit 1: -21 to-14 days, duration 1 week): Participants sign the informed consent form at Visit 1 and complete a series of examinations (see Flowchart Visit 1 for details). The investigator evaluates whether the participant meets the inclusion criteria based on the examination results and exclusion criteria, ensuring no participant fails to meet the exclusion criteria.<br>2. Autologous hematopoietic stem cell transplantation pretreatment (Visit 2-3: -10 to-2 days, duration 9 days): After returning to the research center for hospitalization according to the notified time by the investigator, the subject received autologous hematopoietic stem cell transplantation pretreatment on day-10 to-2 of the trial.<br>3. Stem cell infusion (Visit 3: 0 days, duration 1 day): Autologous hematopoietic stem cells were infused on day 0 of the trial;<br>4. Short-term follow-up period (Visits 4-10:0-180 days, duration 6 months): Subjects returned to the research center monthly for a series of examinations (see Flowchart Visits 5-10) within 6 months after receiving the expected dose of anti-CD19 CAR-T/anti-CD22 CAR-T cells on Days 2 and 4 of the trial. Investigators evaluated the short-term safety and efficacy of the anti-CD19 CAR-T/anti-CD22 CAR-T therapy based on |

| | |
|---|---|
| | the subjects' examination results.<br>5. Medium-to long-term follow-up period (Visits 11-13:180-360 days, duration 6 months): Within 6 months after completing the short-term follow-up, participants return to the research center every 2 months for a series of examinations (see the flowchart for Visits 11-13). Investigators will evaluate the medium-to long-term safety and efficacy of the anti-CD19 CAR-T/anti-CD22 CAR-T therapy based on the participants' examination results.<br>6. Withdrawal from Visit (at Any Time): All subjects may withdraw from the study at any stage of the trial, regardless of providing a reason. If a subject withdraws from the study after receiving anti-CD19 CAR-T/anti-CD22 CAR-T cell infusion, they must complete the withdrawal requirements specified in the visit flowchart. If a subject withdraws from the visit before receiving anti-CD19 CAR-T/anti-CD22 CAR-T cell infusion, only the safety assessment items need to be completed, and the investigator must document the reason for withdrawal in detail. If a subject discontinues treatment due to an adverse event (AE), the investigator must conduct necessary safety follow-up until the AE returns to baseline levels or reaches a stable state. |
| **evaluating indicator** | Main evaluation indicators:<br>The incidence of treatment-related adverse events (grade 3 or 4 adverse reactions, CTCAE V4.0 criteria). This includes adverse events associated with anti-CD19 CAR-T/anti-CD22 CAR-T therapy, serious adverse events, and clinically significant laboratory abnormalities.<br>Secondary evaluation indicators:<br>1. Objective response rates (ORR) of tumors at 3 months, 6 months, and 1 year post-treatment follow-up: complete response (CR) and partial response (PR); |

| | |
|---|---|
| | 2. overall survival (OS);<br>3. progression-free survival (PFS);<br>4. event-free survival (EFS);<br>5. The temporal variation characteristics of anti-CD19 CAR-T/anti-CD22 CAR-T cell expansion levels in subjects (peripheral blood, bone marrow, cerebrospinal fluid, and lymph nodes, etc.).<br>6. the duration of anti-CD19 CAR-T/anti-CD22 CAR-T cells 'persistence in the subject's body (peripheral blood, bone marrow, cerebrospinal fluid, and lymph nodes, etc.)<br>7. The characteristics of lymphocyte depletion in subjects. |
| **statistical analysis** | Statistical analysis was performed with SAS 9.2 software.<br>The primary and secondary study endpoints were analyzed using the ITT (Intention-to-Treat) population. The statistical analysis for the primary endpoint involved describing the number of treatment-related adverse events and their incidence rates, as well as comparing the differences in vital signs and laboratory parameters before and after treatment to determine whether these differences were statistically significant.<br>The statistical analysis method for the secondary endpoint was to describe the tumor ORR based on different follow-up durations. The Kaplan-Meier method and survival curves were used to describe OS, PFS, and EFS. Curves were plotted to illustrate the temporal changes in the expansion levels of anti-CD19 CAR-T and anti-CD22 CAR-T cells, the characteristics of B/T lymphocyte depletion in vivo, and the replication patterns of retroviruses in vivo, with quantitative descriptions of their persistence duration. |

# Treatment Flowchart

| | Filter period | transplantation pretreatment | reinfusion of stem cells | Short-term follow-up period (monthly) | | | | | | | Medium to long-term follow-up period (every two months) | | | Exit follow-up |
|---|---|---|---|---|---|---|---|---|---|---|---|---|---|---|
| Visit Point | Visit 1 | Visit 2 | Visit 3 | Visit 4 | Visit 5 | Visit 6 | Visit 7 | Visit 8 | Visit 9 | Visit 10 | Visit 11 | Visit 12 | Visit 13 | Visit 14 |
| Visit time window | 14 days before transplantation | -10～-2 | 0 | +2，+4 | 30±1 | 60±1 | 90±1 | 120±1 | 150±1 | 180±1 | 240±5 | 300±5 | 360±5 | at any time |
| informed consent | X | | | | | | | | | | | | | |
| Evaluation of admission criteria | X | | | | | | | | | | | | | |
| Demographic information | X | | | | | | | | | | | | | |

| | Filter period | transplantation pretreatment | reinfusion of stem cells | Short-term follow-up period (monthly) | | | | | | | Medium to long-term follow-up period (every two months) | | | Exit follow-up |
|---|---|---|---|---|---|---|---|---|---|---|---|---|---|---|
| Visit Point | Visit 1 | Visit 2 | Visit 3 | Visit 4 | Visit 5 | Visit 6 | Visit 7 | Visit 8 | Visit 9 | Visit 10 | Visit 11 | Visit 12 | Visit 13 | Visit 14 |
| Visit time window | 14 days before transplantation | -10～-2 | 0 | +2, +4 | 30±1 | 60±1 | 90±1 | 120±1 | 150±1 | 180±1 | 240±5 | 300±5 | 360±5 | at any time |
| anamnesis | X | | | | | | | | | | | | | |
| Previous treatment history | X | | | | | | | | | | | | | |
| Vital signs, physical examination [1] | X | X | | X | X | X | X | X | X | X | X | X | X | X |
| Physical | X | X | | X | X | X | X | X | X | X | X | X | X | X |

| | Filter period | transplantation pretreatment | reinfusion of stem cells | Short-term follow-up period (monthly) | | | | | | | Medium to long-term follow-up period (every two months) | | | Exit follow-up |
|---|---|---|---|---|---|---|---|---|---|---|---|---|---|---|
| Visit Point | Visit 1 | Visit 2 | Visit 3 | Visit 4 | Visit 5 | Visit 6 | Visit 7 | Visit 8 | Visit 9 | Visit 10 | Visit 11 | Visit 12 | Visit 13 | Visit 14 |
| Visit time window | 14 days before transplantation | -10～-2 | 0 | +2，+4 | 30±1 | 60±1 | 90±1 | 120±1 | 150±1 | 180±1 | 240±5 | 300±5 | 360±5 | at any time |
| Examination [2] | | | | | | | | | | | | | | |
| Neurological evaluation [3] | X | X | | X | X | X | X | X | X | X | X | X | X | X |
| Blood Pregnancy Test | X | | | | | | | | | | | | | X |
| Complete Blood Count [4] | X | | X | X | X | X | X | X | X | X | X | X | X | X |
| Blood | X | | X | X | X | X | X | X | X | X | X | X | X | X |

| | Filter period | transplantation pretreatment | reinfusion of stem cells | Short-term follow-up period (monthly) | | | | | | | Medium to long-term follow-up period (every two months) | | | Exit follow-up |
|---|---|---|---|---|---|---|---|---|---|---|---|---|---|---|
| Visit Point | Visit 1 | Visit 2 | Visit 3 | Visit 4 | Visit 5 | Visit 6 | Visit 7 | Visit 8 | Visit 9 | Visit 10 | Visit 11 | Visit 12 | Visit 13 | Visit 14 |
| Visit time window | 14 days before transplantation | -10～-2 | 0 | +2，+4 | 30±1 | 60±1 | 90±1 | 120±1 | 150±1 | 180±1 | 240±5 | 300±5 | 360±5 | at any time |
| Biochemistry [5] | | | | | | | | | | | | | | |
| coagulation function | X | | | X | X | X | X | X | X | X | X | X | X | X |
| electrocardiogram | X | | | X | X | | X | | X | | X | X | X | X |
| CD19 and CD22 testing [6] | X | | | | X | X | X | X | X | X | X | X | X | X |
| HBV, HCV, and | X | | | | | | | | | X | | | | |

| | Filter period | transplantation pretreatment | reinfusion of stem cells | Short-term follow-up period (monthly) | | | | | | | Medium to long-term follow-up period (every two months) | | | Exit follow-up |
|---|---|---|---|---|---|---|---|---|---|---|---|---|---|---|
| **Visit Point** | Visit 1 | Visit 2 | Visit 3 | Visit 4 | Visit 5 | Visit 6 | Visit 7 | Visit 8 | Visit 9 | Visit 10 | Visit 11 | Visit 12 | Visit 13 | Visit 14 |
| **Visit time window** | 14 days before transplantation | -10～-2 | 0 | +2，+4 | 30±1 | 60±1 | 90±1 | 120±1 | 150±1 | 180±1 | 240±5 | 300±5 | 360±5 | at any time |
| **HIV** [7] | | | | | | | | | | | | | | |
| **Peripheral blood EBV-DNA titer** [8] | X | | | | X | | X | | X | | X | X | X | X |
| **Detection of lentiviral copy number in vivo** | X | | | | X | X | X | X | X | X | X | X | X | X |

| | Filter period | transplantation pretreatment | reinfusion of stem cells | Short-term follow-up period (monthly) | | | | | | | Medium to long-term follow-up period (every two months) | | | Exit follow-up |
|---|---|---|---|---|---|---|---|---|---|---|---|---|---|---|
| Visit Point | Visit 1 | Visit 2 | Visit 3 | Visit 4 | Visit 5 | Visit 6 | Visit 7 | Visit 8 | Visit 9 | Visit 10 | Visit 11 | Visit 12 | Visit 13 | Visit 14 |
| Visit time window | 14 days before transplantation | -10～-2 | 0 | +2, +4 | 30±1 | 60±1 | 90±1 | 120±1 | 150±1 | 180±1 | 240±5 | 300±5 | 360±5 | at any time |
| (q-PCR) [9] | | | | | | | | | | | | | | |
| Plain head CT scan | X | | | | | | | | | | | | | |
| Pulmonary function test [10] | X | | | | X* | | X* | | X* | | X* | X* | X* | X* |
| Cardiac assessment [11] | X | | | | X* | | X* | | X* | | X* | X* | X* | X* |
| Chest CT or full | X | | | | X* | | X* | | X* | | X* | X* | X* | X* |

| | Filter period | transplantation pretreatment | reinfusion of stem cells | Short-term follow-up period (monthly) | | | | | | | Medium to long-term follow-up period (every two months) | | | Exit follow-up |
|---|---|---|---|---|---|---|---|---|---|---|---|---|---|---|
| Visit Point | Visit 1 | Visit 2 | Visit 3 | Visit 4 | Visit 5 | Visit 6 | Visit 7 | Visit 8 | Visit 9 | Visit 10 | Visit 11 | Visit 12 | Visit 13 | Visit 14 |
| Visit time window | 14 days before transplantation | -10～-2 | 0 | +2, +4 | 30±1 | 60±1 | 90±1 | 120±1 | 150±1 | 180±1 | 240±5 | 300±5 | 360±5 | at any time |
| chest anteroposterior radiograph | | | | | | | | | | | | | | |
| Imaging evaluation [12] | X | | | | X | X | X | X | X | X | X | X | X | X |
| Bone marrow aspiration/biopsy [13] | X | | | | X | X | X | X | X | X | | | | X |

| | Filter period | transplantation pretreatment | reinfusion of stem cells | Short-term follow-up period (monthly) | | | | | | | Medium to long-term follow-up period (every two months) | | | Exit follow-up |
|---|---|---|---|---|---|---|---|---|---|---|---|---|---|---|
| Visit Point | Visit 1 | Visit 2 | Visit 3 | Visit 4 | Visit 5 | Visit 6 | Visit 7 | Visit 8 | Visit 9 | Visit 10 | Visit 11 | Visit 12 | Visit 13 | Visit 14 |
| Visit time window | 14 days before transplantation | -10～-2 | 0 | +2，+4 | 30±1 | 60±1 | 90±1 | 120±1 | 150±1 | 180±1 | 240±5 | 300±5 | 360±5 | at any time |
| Lumbar puncture [14] | X | | | | X | X | X | X | X | X | | | | X |
| Flow cytometry detection of peripheral blood CAR-T cells [15] | | | | | X | X | X | X | X | X | X | X | X | X |
| Flow cytometry detection of | | | | | X | X | X | X | X | X | X | X | X | X |

| | Filter period | transplantation pretreatment | reinfusion of stem cells | Short-term follow-up period (monthly) | | | | | | | Medium to long-term follow-up period (every two months) | | | Exit follow-up |
|---|---|---|---|---|---|---|---|---|---|---|---|---|---|---|
| Visit Point | Visit 1 | Visit 2 | Visit 3 | Visit 4 | Visit 5 | Visit 6 | Visit 7 | Visit 8 | Visit 9 | Visit 10 | Visit 11 | Visit 12 | Visit 13 | Visit 14 |
| Visit time window | 14 days before transplantation | -10～-2 | 0 | +2, +4 | 30±1 | 60±1 | 90±1 | 120±1 | 150±1 | 180±1 | 240±5 | 300±5 | 360±5 | at any time |
| peripheral blood lymphocyte subsets [16] | | | | | | | | | | | | | | |
| Cytokine Detection [17] | X | | | X | X | X | X | X | X | X | | | | X |
| Ferritin [18] | X | | | X | X | X | X | X | X | X | | | | X |
| C-reactive protein test [19] | X | | | X | X | X | X | X | X | X | | | | X |

| | Filter period | transplantation pretreatment | reinfusion of stem cells | Short-term follow-up period (monthly) | | | | | | | Medium to long-term follow-up period (every two months) | | | Exit follow-up |
|---|---|---|---|---|---|---|---|---|---|---|---|---|---|---|
| Visit Point | Visit 1 | Visit 2 | Visit 3 | Visit 4 | Visit 5 | Visit 6 | Visit 7 | Visit 8 | Visit 9 | Visit 10 | Visit 11 | Visit 12 | Visit 13 | Visit 14 |
| Visit time window | 14 days before transplantation | -10～-2 | 0 | +2，+4 | 30±1 | 60±1 | 90±1 | 120±1 | 150±1 | 180±1 | 240±5 | 300±5 | 360±5 | at any time |
| Collection of peripheral blood mononuclear cells (PBMCs) [20] | X | | | | | | | | | | | | | |
| reinfusion of hematopoietic stem cells | | | X | | | | | | | | | | | |
| reinfusion of | | | | X | | | | | | | | | | |

| | Filter period | transplantation pretreatment | reinfusion of stem cells | Short-term follow-up period (monthly) | | | | | | | Medium to long-term follow-up period (every two months) | | | Exit follow-up |
|---|---|---|---|---|---|---|---|---|---|---|---|---|---|---|
| Visit Point | Visit 1 | Visit 2 | Visit 3 | Visit 4 | Visit 5 | Visit 6 | Visit 7 | Visit 8 | Visit 9 | Visit 10 | Visit 11 | Visit 12 | Visit 13 | Visit 14 |
| Visit time window | 14 days before transplantation | -10～-2 | 0 | +2, +4 | 30±1 | 60±1 | 90±1 | 120±1 | 150±1 | 180±1 | 240±5 | 300±5 | 360±5 | at any time |
| CAR-T cells | | | | | | | | | | | | | | |
| Combined treatment record [21] | X | X | X | X | X | X | X | X | X | X | X | X | X | X |
| Drug record [22] | X | X | X | X | X | X | X | X | X | X | X | X | X | X |
| Adverse Event Record | X | X | X | X | X | X | X | X | X | X | X | X | X | X |

*Optional for examination.

**Notes:**

1. Vital signs assessment: Vital signs assessments shall include, at a minimum, measurements of blood pressure, heart rate, respiratory rate, and body temperature. During Visits 2 to 5, patients should measure and record axillary temperature and blood pressure (both systolic and diastolic) twice daily, in the morning (08:00) and evening (20:00).
2. Physical examination: Including general condition, skin examination, and palpation of superficial lymph nodes (particularly Waldeyer's ring), liver, spleen, and abdominal masses.
3. Neurological evaluation: A cranial CT scan should be performed at Visit 1. During follow-up, particular attention should be paid to the occurrence of neurotoxic manifestations, including aphasia, confusion, delirium, lethargy, and hallucinations. Additional neurological examinations should be performed when clinically indicated.
4. Complete blood count (CBC): In addition to the time points specified in the visit flowchart, CBC should be re-evaluated every 1-3 days after CAR-T cell infusion based on the patient's clinical condition. CBC should also be performed on Days 9 and 19 prior to lumbar puncture.
5. Blood biochemistry: Laboratory assessments for hepatic and renal function shall include, at a minimum, serum lactate dehydrogenase (LDH), β2-microglobulin, total bilirubin, direct and indirect bilirubin, creatinine, alanine aminotransferase (ALT), aspartate aminotransferase (AST), cholesterol, blood glucose, and albumin. In addition to scheduled assessments, blood biochemistry tests should be performed every 3-5 days within one month after CAR-T infusion.
6. CD19 and CD22 expression assessment: Results obtained within 3 months prior to enrollment are considered valid and may exempt the patient from repeat testing at Visit 1. Visit 1 is conducted to confirm $CD19^+$/$CD22^+$ status by immunohistochemistry or flow cytometry. Subsequent visits are used to monitor CD19 and CD22 expression dynamics, which may assist in predicting disease recurrence and identifying downregulation or antigen loss. In addition to scheduled time points, CD19 and CD22 expression should be assessed every 3-5 days within one month after reinfusion. For CAR-T products involving immune checkpoint modulation (e.g., PD-1), immunohistochemical assessment of CD30 and PD-L1 should be performed prior to enrollment. Flow cytometry shall be used during follow-up to

monitor CD19, CD22, and CAR-T cell PD-1 expression in peripheral blood at the specified time points and every 3-5 days within one month post-reinfusion.

7. HBV, HCV, and HIV testing: Results obtained within 3 months prior to enrollment are valid and may exempt patients from repeat testing.
8. Peripheral blood EBV DNA testing: Results obtained within 3 months prior to enrollment are valid and may exempt patients from Visit 1 testing. Visit 1 is used to assess EBV DNA titers. If results are within the normal range, further testing is not required. If EBV DNA titers are elevated, testing should be continued at subsequent visits according to the flowchart.
9. In vivo lentiviral copy number detection: Quantitative PCR (qPCR) shall be used to measure lentiviral copy numbers. In addition to scheduled assessments, testing should be performed every 3-5 days within one month after CAR-T cell reinfusion.
10. Pulmonary function tests: Assessments shall include forced expiratory volume in 1 second (FEV1), forced vital capacity (FVC), and diffusing capacity for carbon monoxide (DLCO). Pulmonary function should be carefully evaluated, particularly in patients with prior exposure to bleomycin.
11. Cardiac assessment: Based on prior treatment history, cardiac function should be comprehensively evaluated, particularly in patients previously treated with anthracyclines (e.g., adriamycin). Assessments include echocardiography, electrocardiogram (ECG), troponin, and NT-pro-BNP, with repeat testing as clinically indicated.
12. Imaging evaluation: At baseline, lymphoma patients should undergo imaging of the neck, chest, abdomen, and pelvis. Subsequent imaging should be performed during follow-up to monitor lesion changes and assess therapeutic response.
13. Bone marrow aspiration/biopsy: Bone marrow examination is used for disease diagnosis, assessment of marrow involvement, evaluation of treatment response (e.g., blast count), and monitoring CAR-T cell distribution, density, and persistence. In addition to scheduled time points, bone marrow aspiration should be performed on Days 10 and 20 post-CAR-T infusion to assess CAR-T phenotype and the proportion of CAR-T cells relative to $CD3^+$ T cells by flow cytometry.
14. Lumbar puncture: Lumbar puncture at Visit 1 is performed to assess central nervous system

involvement. Subsequent evaluations are used to monitor neurological improvement and CAR-T cell distribution, density, and persistence in cerebrospinal fluid. Additional lumbar punctures should be performed on Days 10 and 20 after CAR-T infusion for flow cytometric analysis of CAR-T cells in cerebrospinal fluid.

15. Peripheral blood CAR-T cell flow cytometry: Flow cytometry shall be used to monitor CAR-T cell phenotype, density, molecular characteristics, and in vivo persistence. In addition to scheduled assessments, testing should be performed every 3-5 days within one month after reinfusion.
16. Peripheral blood lymphocyte subset analysis: Flow cytometry is used to quantify lymphocyte subsets and their differentiation status, serving as an indirect biomarker of CAR-T efficacy and aiding in prediction of disease recurrence and determination of intravenous immunoglobulin (IVIG) supplementation cycles.
17. Cytokine analysis: Cytokines including, but not limited to, IL-6, IL-10, IFN-γ, and TNF-α shall be monitored to assess cytokine release syndrome severity. In addition to scheduled time points, cytokine testing should be performed every 3-5 days within one month after reinfusion.
18. Ferritin: Serum ferritin shall be monitored as an indicator of macrophage activation. Additional testing should be performed every 3-5 days within one month after CAR-T infusion.
19. C-reactive protein (CRP): CRP testing is used to evaluate inflammatory status and correlate with cytokine levels. Additional testing should be performed every 3-5 days within one month after CAR-T infusion.
20. Peripheral blood mononuclear cell (PBMC) collection: PBMCs shall be collected for ex vivo CAR-T cell manufacturing.
21. Concomitant therapy documentation: All concomitant medications and supportive treatments administered during the study shall be recorded.
22. Prohibited medication records: Use of prohibited medications, including anticancer agents and immunomodulatory drugs, must be documented throughout the study.
23. Test waiver criteria: If cytokines, CAR-T cells, lentiviral copy numbers, and EBV DNA titers are all below the lower limit of detection in two consecutive visits without confounding

interventions (e.g., corticosteroids, tocilizumab), corresponding tests may be waived in subsequent visits.

**24.** Additional examinations: Investigators may perform additional examinations beyond scheduled visits based on disease status or adverse events, including long-term ECG monitoring, troponin testing, MRI, gastroscopy, or colonoscopy, as clinically indicated.

# I. Research Background

The global incidence of malignant hematologic diseases has been increasing steadily in recent years, ranking among the top ten malignancies worldwide in terms of both incidence and mortality, and posing a substantial threat to public health and socioeconomic development [1-2]. Despite advances in conventional treatment modalities such as chemotherapy, radiotherapy, and hematopoietic stem cell transplantation (HSCT), disease relapse and refractory status remain the most critical challenges in clinical management.

With the rapid development of high-throughput sequencing and molecular profiling technologies, researchers have gained deeper insights into the pathogenesis of hematologic malignancies, enabling refined disease classification and the development of personalized therapeutic strategies. A growing body of evidence indicates that tumor cells evade immune surveillance through multiple mechanisms, including downregulation of major histocompatibility complex (MHC) molecules, attenuation of co-stimulatory signals, and exploitation of immune checkpoint pathways. These immune escape mechanisms ultimately lead to immune tolerance, disease recurrence, and treatment resistance. Consequently, therapeutic strategies aimed at restoring immune surveillance and counteracting tumor immune evasion have emerged as central components of modern cancer therapy.

Immunotherapy was recognized as one of the top ten breakthroughs in cancer treatment by Science in 2013 [3-4]. Among immunotherapeutic approaches, chimeric antigen receptor (CAR) T-cell therapy has garnered particular attention due to its remarkable clinical efficacy. First-generation CAR-T cells were generated by genetically modifying autologous T cells to express tumor antigen-recognizing single-chain variable fragments (scFvs), enabling MHC-independent tumor cell recognition. However, the absence of co-stimulatory signaling domains limited their in vivo persistence and antitumor activity. Subsequent second- and third-generation CAR-T constructs incorporated co-stimulatory domains such as CD28 and CD137 (4-1BB), thereby enhancing T-cell activation, proliferation, persistence, and resistance to tumor-mediated immunosuppression, ultimately improving antitumor efficacy [5].

In 2013, The New England Journal of Medicine reported that two patients with

relapsed/refractory acute lymphoblastic leukemia (ALL) achieved complete remission following adoptive infusion of anti-CD19 CAR-T cells, highlighting the transformative potential of this therapeutic strategy [6]. Since then, CAR-T therapies targeting a variety of tumor-associated antigens-including CD33, CD20, CD30, CD123, and ERBB2-have been developed and evaluated across multiple hematologic malignancies and solid tumors, including ALL, AML, lymphoma, and others [7-11].

**Structure of Chimeric Antigen Receptors**

A chimeric antigen receptor (CAR) is a recombinant antigen receptor that integrates antigen recognition and T-cell activation functions. Structurally, CARs consist of three major regions: an extracellular domain, a transmembrane domain, and an intracellular signaling domain. Functionally, classical CARs comprise five key components: (1) an scFv derived from monoclonal antibodies that recognizes tumor surface antigens; (2) a hinge region that provides structural flexibility and facilitates antigen binding; (3) a transmembrane domain that anchors the receptor to the T-cell membrane; (4) one or more co-stimulatory domains that enhance T-cell activation and persistence; and (5) the CD3ζ signaling domain responsible for initiating T-cell activation.

**Mechanism of Action of CAR-T Therapy [12]**

CAR-T therapy involves genetic engineering of autologous T cells to express CAR constructs targeting tumor-associated antigens (TAAs). Specifically, scFv sequences recognizing TAAs are recombinantly fused with intracellular signaling domains containing immunoreceptor tyrosine-based activation motifs (ITAMs), typically derived from CD3ζ or FcεRIγ, to generate recombinant plasmids. These plasmids are transfected into T cells ex vivo, enabling stable CAR expression. The modified T cells are subsequently expanded, purified, and subjected to stringent quality control prior to reinfusion into the patient at a predetermined dose.

Upon infusion, CAR-T cells recognize tumor antigens in an MHC-independent manner, triggering intracellular signaling cascades that lead to T-cell activation, proliferation, and cytotoxic effector function. Activated CAR-T cells release perforin, granzymes, and pro-inflammatory cytokines, inducing tumor cell apoptosis and mediating potent antitumor effects.

**Advantages of CAR-T Therapy [13-17]**

CAR-T therapy offers several advantages over conventional T-cell-based

immunotherapies: CAR-T cells bypass MHC restriction, overcoming tumor immune escape mediated by MHC downregulation; Both protein and glycolipid antigens can serve as therapeutic targets, broadening the spectrum of actionable tumor antigens; The inclusion of co-stimulatory signaling motifs promotes T-cell proliferation, survival, and resistance to immunosuppressive tumor microenvironments; CAR technology can be applied not only to T cells but also to natural killer (NK) cells and cytokine-induced killer (CIK) cells, expanding therapeutic applications. With ongoing optimization of CAR design and manufacturing technologies, CAR-T therapy is expected to play an increasingly prominent role in cellular immunotherapy for cancer.

**Clinical Workflow of CAR-T Therapy**

The clinical application of CAR-T therapy typically involves five key steps: Isolation of T cells or mononuclear cells from the patient's peripheral blood; Genetic modification of T cells to express CAR constructs targeting tumor antigens; Ex vivo expansion of CAR-T cells to the target infusion dose (typically 1-9 × 10^5-10^6 cells/kg); Administration of non-myeloablative lymphodepleting chemotherapy prior to infusion to reduce tumor burden and suppress immunosuppressive cells; Infusion of CAR-T cells followed by close monitoring of therapeutic response and adverse events.

**Safety Profile of CAR-T Therapy in Clinical Trials**

Clinical trials have demonstrated that CAR-T therapy is associated with a relatively high incidence of adverse events; however, most are manageable with appropriate supportive care [18-20]. The most significant safety concerns include off-target effects and cytokine release syndrome (CRS). In sequential anti-CD19 and anti-CD22 CAR-T therapies, off-target effects primarily manifest as B-cell aplasia, which may also serve as a surrogate marker of CAR-T cell persistence and activity. Although intravenous immunoglobulin (IVIG) can mitigate B-cell depletion-related complications, severe cases may be life-threatening.

CRS is a potentially severe inflammatory syndrome characterized by high fever, hypotension, fatigue, confusion, and organ dysfunction. Clinical data indicate that CRS severity correlates with elevated serum levels of inflammatory cytokines, including IFN-γ, TNF-α, and IL-6. Management strategies for CRS include corticosteroids, which suppress T-cell activity but may compromise CAR-T efficacy, and cytokine-targeted therapies. Tocilizumab, an IL-6 receptor antagonist, effectively

attenuates CRS without impairing CAR-T-mediated antitumor activity. Serum C-reactive protein (CRP), an acute-phase reactant synthesized by the liver, has emerged as a practical biomarker for monitoring CRS severity due to its accessibility and rapid turnaround time. Accordingly, CRP is incorporated in this protocol for CRS monitoring.

**Risk-Benefit Assessment**

Accumulating evidence indicates that recombinant CAR-T cells targeting tumor surface antigens, particularly anti-CD19 CAR-T cells, exhibit substantial antitumor activity in patients with B-cell malignancies. Based on existing clinical data, patients who may benefit from sequential anti-CD19/anti-CD22 CAR-T therapy include those with relapsed disease following standard therapy or HSCT, patients temporarily ineligible for HSCT, and refractory patients. The National Comprehensive Cancer Network (NCCN) guidelines consistently recommend clinical trial participation for relapsed/refractory patients.

Although CAR-T therapy is associated with a high incidence of adverse drug reactions, the majority are manageable with timely intervention. Overall, the anticipated clinical benefits outweigh the potential risks, supporting the ethical and scientific justification for subject participation in this study.

## II. Research Objectives

1. Primary Research Objective:

   To evaluate the safety of sequential infusion of anti-CD19 CAR-T and anti-CD22 CAR-T cells following autologous hematopoietic stem cell transplantation (auto-HSCT) in patients with relapsed/refractory or high-risk B-cell lymphoma.

2. Secondary Research Objectives:

1) To evaluate the clinical efficacy of sequential infusion of anti-CD19 CAR-T and anti-CD22 CAR-T cells following autologous hematopoietic stem cell transplantation in patients with relapsed/refractory or high-risk B-cell lymphoma;
2) To assess the in vivo expansion kinetics and persistence of anti-CD19 CAR-T and anti-CD22 CAR-T cells in recipients after autologous hematopoietic stem cell transplantation.

# III. Research Plan

## 3.1 Overview of the Research Plan

This study is designed as a Phase I/II, single-center, open-label, single-arm clinical trial. A total of 150 subjects are planned to be enrolled to evaluate the safety and efficacy of sequential anti-CD19 CAR-T and anti-CD22 CAR-T cell therapy following autologous hematopoietic stem cell transplantation in patients with relapsed/refractory or high-risk B-cell lymphoma. The trial consists of the following six visit periods (see the visit flowchart for details):

1) Screening Period (Visit 1: Days −14 to −7; duration: 1 week): Participants provide written informed consent at Visit 1 and complete a series of screening assessments (see Flowchart Visit 1). Based on the examination results and predefined inclusion and exclusion criteria, investigators determine participant eligibility for study enrollment.
2) Autologous Hematopoietic Stem Cell Transplantation Pretreatment Period (Visits 2–3: Days −10 to −2; duration: 9 days): Subjects return to the research center for hospitalization according to the investigator's notification and receive pretreatment for autologous hematopoietic stem cell transplantation from Day −10 to Day −2 of the trial.
3) Stem Cell Infusion Period (Visit 3: Day 0; duration: 1 day): Autologous hematopoietic stem cells are infused on Day 0 of the trial.
4) Short-Term Follow-up Period (Visits 4–10: Days 0–180; duration: 6 months): Within 6 months after receiving the planned doses of anti-CD19 CAR-T and anti-CD22 CAR-T cells on Days 0 and 2, subjects return to the research center for monthly follow-up visits and examinations (see Flowchart Visits 5–10). Investigators assess the short-term safety and efficacy of the CAR-T therapies based on clinical and laboratory findings.
5) Medium- to Long-Term Follow-up Period (Visits 11–13: Days 180–360; duration: 6 months): After completion of the short-term follow-up period, subjects return every 2 months for follow-up assessments (see Flowchart Visits 11–13). Investigators evaluate the medium- to long-term safety and efficacy of the CAR-T therapies.

6) Withdrawal from the Study (At Any Time): Subjects may withdraw from the study at any stage without providing a reason. If withdrawal occurs after CAR-T cell infusion, subjects must complete the withdrawal-related assessments specified in the visit flowchart. If withdrawal occurs before CAR-T cell infusion, only safety assessments are required, and investigators must document the reason for withdrawal in detail. If treatment is discontinued due to adverse events (AEs), investigators must conduct appropriate safety follow-up until the AE resolves, returns to baseline, or stabilizes.

### 3.2 Rationale for the Research Plan

Sequential infusion of anti-CD19 and anti-CD22 CAR-T cells following autologous hematopoietic stem cell transplantation is an individualized therapeutic approach that requires collection of a subject's peripheral blood mononuclear cells, genetic modification and ex vivo expansion of CAR-T cells, and subsequent reinfusion into the subject. Because the in vivo expansion and persistence of CAR-T cells cannot be predicted, implementation of a blinded study design is not feasible. Therefore, this study is designed as a single-center, open-label trial. Furthermore, the investigational intervention focuses on evaluating the safety and efficacy of anti-CD19 and anti-CD22 CAR-T cell therapy. Currently, CAR-T cell manufacturing methods and technologies vary among research institutions, and this therapeutic approach remains in the clinical research stage worldwide, without a standardized comparator regimen. Consequently, a controlled trial design is not appropriate, and the study is therefore designed as a single-arm clinical trial.

## IV. Study Population

### 4.1 Eligibility Criteria

1. The patient or their legally authorized representative voluntarily agrees to participate in the study and provides written informed consent.
2. Male or female patients aged 18 to 70 years (inclusive).

3. The patient has been pathologically and histologically diagnosed with CD19-positive and/or CD22-positive B-cell lymphoma, meets the criteria for autologous hematopoietic stem cell transplantation (auto-HSCT), and voluntarily undergoes sequential infusion of anti-CD19 CAR-T and anti-CD22 CAR-T cells following auto-HSCT.
4. The subject is eligible for auto-HSCT and meets at least one of the following conditions:

(1) Presence of residual disease or disease progression after first-line therapy;

(2) Relapse after first complete remission (CR1);

(3) Presence of recognized high-risk factors;

(4) Disease relapse or failure to achieve remission after prior cellular immunotherapy;

(5) Patients for whom allogeneic hematopoietic stem cell transplantation is indicated but not feasible or contraindicated.

5. Adequate function of major organs, defined as:

(1) Hepatic function: ALT and AST <3 × upper limit of normal (ULN), and total bilirubin ⩽34.2 μmol/L;

(2) Renal function: Serum creatinine <220 μmol/L;

(3) Pulmonary function: Resting oxygen saturation ⩾95% on room air;

(4) Cardiac function: Left ventricular ejection fraction (LVEF) ⩾40%.

6. The patient has not received any anticancer therapy (including chemotherapy, radiotherapy, or immunotherapy such as immunosuppressive agents) within 4 weeks prior to enrollment, and any treatment-related toxicities have resolved to Grade ⩽ 1 (CTCAE), except for alopecia or other clinically insignificant toxicities.
7. Adequate peripheral venous access suitable for intravenous infusion.
8. Eastern Cooperative Oncology Group (ECOG) performance status ⩽2 and an estimated life expectancy of ⩾3 months.

### 4.2 Exclusion Criteria

1. Pregnant or breastfeeding women (confirmed by urine or blood pregnancy testing);
2. Male or female patients planning to conceive within 1 year after enrollment;
3. Inability or unwillingness to use effective contraception (e.g., condoms, oral contraceptives) for 1 year after enrollment;
4. Presence of uncontrolled infection within 4 weeks prior to enrollment;
5. Active hepatitis B or hepatitis C infection;
6. Known human immunodeficiency virus (HIV) infection;
7. Severe autoimmune diseases or primary immunodeficiency disorders;
8. History of severe allergic reactions or hypersensitivity to macromolecular biologics such as antibodies or cytokines;
9. Participation in another interventional clinical trial within 6 weeks prior to enrollment;
10. Systemic corticosteroid therapy within 4 weeks prior to enrollment (excluding inhaled corticosteroids);
11. Severe psychiatric illness that may interfere with study compliance;
12. History of drug abuse or substance dependence;
13. Any other condition deemed by the investigator to make the patient unsuitable for study participation.

### 4.3 Withdrawal Criteria

Participants may withdraw from the study at any time without providing a reason. Withdrawal will not result in discrimination, retaliation, or compromise of the participant’s entitled medical care. If a participant who does not meet the inclusion criteria is inadvertently enrolled, the participant shall be withdrawn immediately upon discovery. The research institution or its authorized representative must be notified promptly, and a replacement participant may be enrolled if appropriate. Unless

exceptional circumstances apply, participants shall be withdrawn from the study if the investigator determines that continued participation poses a serious or life-threatening safety risk. Participants may discontinue anti-CD19 CAR-T and anti-CD22 CAR-T therapy or withdraw from the study under the following conditions:

1. Occurrence of intolerable adverse events (AEs) or clinically significant laboratory abnormalities, as assessed by the investigator; appropriate medical measures shall be taken and reported to the research institution or its designated personnel.
2. Voluntary refusal by the participant to continue study participation.
3. Poor protocol adherence that significantly compromises the integrity of safety or efficacy evaluations.
4. Termination of the study by the investigator or research institution for any reason.

If withdrawal occurs after CAR-T cell infusion, all exit assessments specified in the visit flowchart must be completed. If withdrawal occurs before CAR-T cell infusion, only safety-related assessments are required, and the investigator must document the reason for withdrawal in detail. Participants who withdraw early do not require replacement.

### 4.4 Suspension Criteria

1. Occurrence of serious safety concerns during the trial;
2. Request for termination by the study site or research unit;
3. Identification of major protocol deficiencies that prevent adequate evaluation of safety or efficacy;
4. Suspension requested by regulatory or administrative authorities.

## V. Treatment Protocol

### 5.1 Patient Screening; Cell Collection, Modification, and Expansion

(1) Patients are admitted to the hospital approximately 2 weeks prior to CAR-T cell reinfusion and are required to sign the informed consent form. Subsequently, a

series of screening examinations are completed (see Flowchart Visit 1). Based on the examination results and the predefined inclusion and exclusion criteria, the investigator determines whether the patient is eligible for enrollment.

(2) For eligible patients, peripheral venous blood is collected or peripheral blood mononuclear cells (PBMCs) are obtained using a leukapheresis device on Day −10. The collected peripheral blood or PBMCs are transported to the central laboratory under refrigerated conditions (4℃) for further processing.

(3) The genetic modification, activation, and ex vivo expansion of CAR-T cells typically require approximately 7–10 days. Based on the estimated time required for CAR-T cells to reach the target infusion dose and the status of cell culture expansion, the central laboratory coordinates with the clinical team to determine the timing of non-myeloablative conditioning for each patient.

## 5.2 Autologous Hematopoietic Stem Cell Transplantation

(1) Pre-treatment begins on day-10.

(2) Autologous hematopoietic stem cells were transfused on day 0.

## 5.3 CAR-T Cell Infusion

CAR-T cells will be administered intravenously on Day 0 using a standard infusion set, with supplemental oxygen provided as needed. Continuous monitoring, including electrocardiography, blood pressure, and oxygen saturation, will be performed throughout the infusion. The infusion will be completed over approximately 30 minutes, followed by flushing of the infusion line with normal saline.

Note: The infusion of immune checkpoint-modified CAR-T cells and the administered dose may be adjusted based on the subject’s clinical condition and investigator judgment.

### 5.4 Adverse Reactions and Management

During CAR-T cell infusion and the post-infusion follow-up period, subjects will be closely monitored for adverse reactions. Emergency equipment and medications must be readily available during hospitalization. In the event of adverse reactions, prompt symptomatic and supportive treatment shall be initiated. Recommended management measures are described below:

(1) Cytokine Release Syndrome (CRS):

**Table 1. Classification criteria for CRS severity:**

| | |
|---|---|
| **Grade 1** | Mild reactions may occur, including fever, nausea, vomiting, fatigue, headache, myalgia, and irritability. Symptoms can be managed with supportive care alone, such as antihistamines, nonsteroidal anti-inflammatory drugs (NSAIDs), and sedatives. |
| **Grade 2** | Hypotension and/or exertional dyspnea that resolves rapidly (within 24 hours) after moderate-intensity intervention. Management includes low-flow oxygen supplementation ($FiO_2$ <40%), adequate intravenous fluid resuscitation, or the use of a single vasoactive agent. May be accompanied by Grade 2 organ toxicity. |
| **Grade 3** | Severe CRS presenting with one or more of the following manifestations and requiring urgent medical intervention due to critical condition:<br>• A decrease in systolic, diastolic, or mean arterial pressure >20% from baseline that is unresponsive to sustained rapid intravenous fluid resuscitation (>20 mL/kg), or requiring multiple or high-dose vasoactive agents;<br>• Grade 3 respiratory failure, including dyspnea at rest, inability to perform self-care, $SpO_2$ <88% or $PaO_2$ <55 mmHg while receiving oxygen with $FiO_2$ ≥40%; |

| | |
|---|---|
| | • Grade 3 organ toxicity (e.g., heart failure) or Grade 4 hypertransaminemia (>20 × ULN). |
| **Grade 4** | Life-threatening CRS requiring mechanical ventilation, or associated with Grade 4 organ toxicity (excluding isolated hypertransaminemia). |
| **Grade 5** | Death. |

Note: The organ toxicity grading is based on the CTCAE v4.0 grading system.

For patients with suspected severe CRS (grade 3-4), close monitoring of the condition is required, and other possible causes (such as septic shock) should be actively ruled out. If necessary, transfer to the intensive care unit (ICU) is recommended. Treatment should include immunosuppressants, with tocilizumab, an IL-6 receptor antagonist, as the first-line therapy.

(1) B lymphocyte depletion: Intravenous immunoglobulin (IVIG) may be administered as supportive therapy to maintain serum IgG levels and reduce the risk of infection.

(2) Neurotoxicity: Clinical manifestations may include drowsiness, lethargy, stupor, and, in severe cases, seizures. These symptoms are often self-limiting and can be managed with appropriate symptomatic and supportive treatment.

(3) Fever: Management includes physical cooling measures or administration of nonsteroidal anti-inflammatory drugs (NSAIDs). Corticosteroids should be avoided whenever possible unless clinically indicated.

(4) Myalgia: Symptomatic treatment with conventional nonsteroidal anti-inflammatory drugs (NSAIDs) is recommended.

(5) Hypotension: Intravenous fluid resuscitation should be initiated promptly, and vasoactive agents may be administered as needed to maintain adequate blood pressure.

(6) Respiratory distress or respiratory failure: Management includes infection prevention, maintenance of airway patency, and oxygen supplementation. If necessary, non-invasive ventilation (BiPAP) or invasive mechanical ventilation with endotracheal intubation should be performed.

(7) Leukopenia: To prevent and treat infections, empirical broad-spectrum antibiotics may be administered, accompanied by active etiological investigations as clinically indicated.

(8) Coagulation disorders: Coagulation abnormalities may present as prolonged prothrombin time (PT), activated partial thromboplastin time (APTT), or decreased fibrinogen levels, and should be managed with appropriate supportive and symptomatic treatment.

### 5.5 Monitoring and Follow-up

Patients are required to complete Visits 2 through 5 during hospitalization, except for those who withdraw prematurely from the study. The investigator will determine patient eligibility for discharge based on the findings at Visit 5 and the resolution or stabilization of adverse events. Post-discharge follow-up is conducted as follows:

(1) short-term follow-up period

Within 6 months after CAR-T cell infusion, patients are scheduled for monthly hospital visits (Visits 5-10, see Flowchart) to monitor and evaluate the short-term safety and efficacy of CAR-T therapy.

(2) Medium to long-term follow-up period

Within 6 months following Visit 9, patients will return for follow-up every 2 months (Visits 11-13, see Flowchart) to assess the medium- and long-term safety and efficacy of CAR-T therapy.

(3) Long-term follow-up period

After completion of Visit 13, patients will be followed up by telephone every 4 months until disease progression, death, or loss to follow-up. Detailed records shall be maintained regarding previously reported adverse events, disease status, and any subsequent treatments.

## VI. Concomitant Therapy and Prohibited Medications

During the 1-year treatment and follow-up period, concomitant therapies may be adjusted if the patient's condition deteriorates or disease progression occurs, or if the investigator determines that such adjustments may improve clinical outcomes.

## VII. Trial Design and Blinding

This study was designed as an open-label trial. As the trial involved individualized treatment, peripheral mononuclear cells (PMCs) from participants were collected, genetically modified to prepare anti-CD19 CAR-T and anti-CD22 CAR-T cells, and reinfused into the participants at the intended dose after passing multiple quality control tests. Therefore, a blinded approach was not feasible, and the trial was designed as an open-label trial.

## VIII. Treatment Compliance

Participant compliance is promoted through the following measures:

1. Investigators shall rigorously implement the informed consent process to ensure that participants fully understand the study objectives, procedures, and requirements prior to enrollment.
2. Costs associated with CAR-T cell preparation — including cell collection, purification, genetic modification, activation, expansion, and quality control— are waived for study participants.
3. During the study, no changes to the infusion timing, dose, volume, or route (intravenous administration) of anti-CD19 or anti-CD22 CAR-T cells may be made without prior written approval from the investigator. All protocol deviations must be fully documented.
4. Investigators shall conduct scheduled study visits in a timely manner, arrange all required assessments, promptly address participant inquiries, and ensure

immediate management and documentation of any adverse events.

# IX. Evaluation Endpoints and Criteria

## 9.1 Primary End Points

Safety outcomes associated with anti-CD19 CAR-T and/or anti-CD22 CAR-T therapy, including the incidence and severity of adverse events (AEs), serious adverse events (SAEs), and clinically significant laboratory abnormalities.

## 9.2 Secondary End Points

1. Objective response rates (ORR) of tumors at 3 months, 6 months, and 1 year post-treatment follow-up: complete response (CR) and partial response (PR);
2. overall survival (OS);
3. progression-free survival (PFS);
4. event-free survival (EFS);
5. Kinetics of in vivo expansion of anti-CD19 CAR-T and anti-CD22 CAR-T cells in subjects, assessed in peripheral blood, bone marrow, cerebrospinal fluid, lymph nodes, and other relevant compartments;
6. Duration of persistence of anti-CD19 CAR-T and anti-CD22 CAR-T cells in vivo;
7. Characteristics and extent of lymphocyte depletion in treated subjects.

## 9.3 Safety Assessments

### 9.3.1 Definition of Adverse Events

The investigator is responsible for documenting all adverse events occurring during the study and for evaluating the onset, duration, severity, causality, management, and outcomes of each AE in relation to anti-CD19 and/or anti-CD22 CAR-T cell therapy.

Adverse Event (AE): An AE is defined as any unfavorable and unintended medical occurrence in a subject receiving anti-CD19 and/or anti-CD22 CAR-T cell therapy, regardless of whether it is considered related to the investigational product. An AE may include any abnormal sign (including abnormal laboratory findings), symptom, or disease temporally associated with the use of the investigational therapy.

Serious Adverse Event (SAE): An SAE refers to any adverse medical occurrence that occurs after administration of any dose of anti-CD19 and/or anti-CD22 CAR-T cells and meets one or more of the following criteria:

- Results in death;
- Is life-threatening (i.e., the subject was at immediate risk of death at the time of the event);
- Requires inpatient hospitalization or prolongation of existing hospitalization (excluding planned hospitalizations not related to disease worsening);
- Results in persistent or significant disability or incapacity;
- Results in a congenital anomaly or birth defect;
- Other medically significant events that may not immediately result in death or hospitalization but require medical or surgical intervention to prevent serious outcomes.

Adverse Drug Reaction (ADR): Any adverse and unintended reaction related to anti-CD19 and/or anti-CD22 CAR-T cell therapy occurring after administration of the investigational product.

Unexpected Adverse Reaction: An adverse reaction whose nature, severity, or frequency is not consistent with the known safety information of anti-CD19 and/or anti-CD22 CAR-T cell therapy.

9.3.2 Severity of adverse events

All adverse events and adverse drug reactions shall be graded according to the National Cancer Institute Common Terminology Criteria for Adverse Events (NCI-CTCAE), Version 4.0.

9.3.3 Relationship between Adverse Events and Study Drug Anti-CD19

CAR-T/anti-CD22 CAR-T Cells

The relationship between anti-CD19 CAR-T/anti-CD22 CAR-T cell therapy and AE was categorized into five types: unrelated, possibly unrelated, possibly related, related, and uncertain (unable to determine).

- If there is no reasonable possibility of association between the adverse event (AE) and the investigational drug anti-CD19 CAR-T/anti-CD22 CAR-T cell therapy, the AE is considered to be unrelated to the investigational drug.
- Non-relevance may refer to clinical events that are not fundamentally or completely related to the investigational drug anti-CD19 CAR-T/anti-CD22 CAR-T cell therapy, but can be explained by other conditions, including chronic diseases, other concomitant drug reactions, or disease progression.
- Potentially relevant refers to clinical events with an unknown association between the adverse event (AE) and the investigational drug anti-CD19 CAR-T/anti-CD22 CAR-T cell therapy, but which cannot be explained by other causes.
- Potential relevance refers to adverse events (AEs) that exhibit a temporal relationship with the investigational drug anti-CD19 CAR-T/anti-CD22 CAR-T cell therapy, and which investigators determine cannot be explained by other causes (complications, disease progression, or concomitant medications).
- Adverse events (AEs) should be classified according to the above categories. An AE may be classified as 'uncertain' (cannot be determined) only when the causal relationship cannot be assessed, for example, due to insufficient evidence, conflicting evidence, conflicting data, or poor documentation quality.

9.3.4 Collection, Documentation, and Reporting of AE

All AEs occurring during the study, regardless of severity or causality, must be collected and recorded in the adverse event section of the Case Report Form (CRF). Required documentation includes the AE description, onset and resolution dates,

severity, actions taken, outcome, and relationship to the investigational product. Each AE shall be recorded as a separate entry.

9.3.5 Reporting of Serious Adverse Events

All SAEs occurring during the study must be reported in accordance with applicable regulatory and ethics committee requirements, regardless of causality.

Investigators shall:

- Initiate appropriate medical management immediately;
- Report the SAE to the study sponsor and the designated safety officer within 24 hours of awareness;
- Record the SAE in the CRF AE and SAE report forms and source documents;
- Submit a signed and dated SAE report to the independent ethics committee within 24 hours;
- Follow the SAE until resolution, stabilization, or return to baseline.

9.3.6 Management and Follow-up of Adverse Events

Investigators are responsible for providing appropriate medical care for all AEs. All AEs observed from screening through study completion shall be followed until resolution, stabilization, or return to baseline.

For trial-related adverse events, all treatment and compensation costs shall be borne by the CAR-T cell preparation unit, Wuhan Boruida Biotechnology Co., Ltd.

9.3.6.1 Clinical Laboratory Evaluations

Investigators shall review laboratory results promptly to assess normality, trends, and clinical significance. Clinically significant abnormal laboratory findings shall be recorded as laboratory AEs. Abnormal laboratory values attributable to the subject's underlying disease shall be documented but not reported as AEs unless clinically significant.

9.3.6.2 Vital Signs, Physical Examination, and Other Safety Assessments

Based on prior clinical research experience, subjects may experience toxicities of varying severity following CAR-T cell infusion. Common toxic reactions include

fever, hypotension, and alterations in mental status, whereas respiratory and cardiac toxicities may be more severe and potentially life-threatening. Therefore, investigators must exercise close clinical vigilance and carefully evaluate the following safety parameters:

Vital signs assessment: Vital signs assessments shall include, at a minimum, measurements of blood pressure, heart rate, respiratory rate, and body temperature. During Visits 2 to 5, subjects shall measure and record axillary temperature and blood pressure (both systolic and diastolic) twice daily, in the morning (08:00) and evening (20:00).

Physical examination: includes general condition, skin examination, palpation of superficial lymph nodes (especially Weil's ring), liver, spleen, and abdominal masses.

Neurological assessment: Close monitoring for neurotoxicity, including aphasia, confusion, mania, seizures, lethargy, and hallucinations.

## X. Data Management

### 10.1 Case Report Filling and Handover

The case report form is completed by the investigator, and each enrolled case must submit a case report form. The completed case report form is reviewed by the clinical monitor, and the first copy is transferred to the data administrator for data entry and management.

### 10.2 Data Entry and Modification

Data entry and management shall be conducted by a professional data management unit. Data administrators shall utilize data management software to develop data entry procedures for data input and administration. To ensure data accuracy, two independent data administrators shall perform duplicate entry and cross-checking.

For questions raised in the case report form, the data administrator will generate

a Data Request Questionnaire (DRQ) and submit it to the investigator via the clinical monitor. The investigator is required to respond promptly. Based on the investigator's responses, the data administrator will modify, confirm, and enter the data. If necessary, the DRQ may be resubmitted.

### 10.3 Data Review and Locking

Upon completion of data management, the primary investigator, data administrator, and statistical analyst jointly confirm the statistical analysis population. After resolving all data-related questions, the database is locked and the review report is signed simultaneously.

## XI. Statistical Analysis

Upon completion of all study assessments and after the database has been cleared and closed, a final analysis will be conducted for the entire study population at the end of the study. The statistical analysis plan, which will include more detailed information about the analysis, will be drafted and finalized prior to the database closure.

### 11.1 Determination of Sample Size

This study enrolled 150 subjects based on practical considerations and the minimum regulatory requirements, with the number of potential participants meeting informed consent.

### 11.2 Statistical Analysis Population

Intent-To-Treat (ITT) population: All subjects who received anti-CD19 CAR-T/anti-CD22 CAR-T cell therapy.

## 11.3 Statistical Analysis Plan

11.3.1 General Principles of Statistical Analysis

Statistical analyses in this study will be performed using SAS 9.2 or later versions. Unless otherwise specified, all hypothesis tests will be conducted as two-tailed tests, with a significance level of $p < 0.05$. P-values will be rounded to four decimal places, and 95% confidence intervals will be calculated when necessary for $p < 0.0001$. Descriptive analysis for single groups will be conducted as follows: continuous variables will be summarized as frequency, mean, standard deviation, median, first quartile, third quartile, minimum, and maximum; categorical variables will be summarized as frequency and percentage.

11.3.2 Statistical Analysis Methods

1) Methods for Deletion and Missing Data Processing

The analysis of secondary endpoints will include data from participants who were dropped out. When using the ITT set for secondary endpoint analysis, secondary endpoints for which the complete treatment course was not observed will be carried forward to the final trial results using the Last Observation Carried Forward (LOCF) method. LOCF will be the primary method for filling in missing data.

2) interim analysis

This study will conduct two interim analyses. The first analysis will be performed when 100% of the participants complete the short-term follow-up period (0-180 days, duration of 6 months), and the second analysis will be performed when 100% of the participants complete the medium-to-long-term follow-up period (180-360 days, duration of 6 months).

3) multicenter study

not applicable.

4) Multiple Comparisons and Adjustments for Multiplicity

not applicable.

5) Subgroup analysis

not applicable.

11.3.3 Statistical Analysis

1) fall off analysis

The total number of subjects who were enrolled and excluded were classified according to the description.

Summarize the subject information by treatment group and primary reason (e.g., loss to follow-up, adverse events [AEs], poor compliance), including the number of subjects enrolled and those who entered or completed each study phase (or week/month), as well as all reasons for discontinuation after enrollment. Elucidate whether subjects who discontinued anti-CD19 CAR-T/anti-CD22 CAR-T cell therapy continued to be followed during the study period.

Provide a list of all subjects who discontinued the study after enrollment, including subject number, specific reason for discontinuation, treatment (anti-CD19 CAR-T/anti-CD22 CAR-T cells and dose), cumulative dose (if applicable), and duration of treatment prior to discontinuation.

2) Demographics and other baseline characteristics

Based on the numerical characteristics of variables, continuous variables (e.g., age, height, weight, etc.) will be summarized using case counts, mean, standard deviation, median, first quartile, third quartile, minimum, and maximum values; categorical variables (e.g., gender, medical history, prior treatment, etc.) will be summarized using case counts and percentages.

3) Analysis of Compliance and Drug Exposure

not applicable.

4) efficiency analysis

For ORR analysis, the results were described according to different follow-up time points after treatment (3 months, 6 months, and 1 year).

The Kaplan-Meier method and survival curves will be used to describe the analysis of OS, PFS, and EFS.

The analysis of the expansion level, distribution characteristics, and persistence duration of anti-CD19 CAR-T/anti-CD22 CAR-T cells in subjects should be

described based on different follow-up time points post-treatment and various tissues in vivo (peripheral blood, bone marrow, cerebrospinal fluid, lymph nodes, etc.), with corresponding characteristics and follow-up time points plotted as line graphs.

Based on the characteristics of lymphocyte depletion and in vivo replication of retroviruses, descriptions should be provided according to different follow-up time points, and line graphs should be plotted using the aforementioned characteristics and follow-up time points.

5) safety analysis

Safety was evaluated by summarizing changes in AE, laboratory test results, and vital signs. Any subject who had received at least one treatment with anti-CD19 CAR-T or anti-CD22 CAR-T cells was included in the safety analysis.

a) adverse event

Safety was evaluated by summarizing changes in AE, laboratory test results, and vital signs. Any subject who had received at least one treatment with anti-CD19 CAR-T or anti-CD22 CAR-T cells was included in the safety analysis.

b) laboratory examination

Descriptive statistics (counts and percentages) of all laboratory test results and changes compared to baseline.

c) ECG check up

not applicable.

d) Vital signs, physical examination, and other safety-related assessments

Descriptive statistics (counts and percentages) of vital signs and changes from baseline.

The analysis and presentation of vital signs, other physical examination findings, and other safety-related observations should follow methods similar to those used for laboratory variables.

e) Other safety analyses

not applicable.

## XII. Quality Assurance and Quality Control

According to the guidance principles of China Good Clinical Practice (GCP), research institutions are responsible for implementing and maintaining quality assurance and quality control systems in accordance with the corresponding standard operating procedures (SOPs) to ensure that the conduct of clinical trials and the collection, recording, and reporting of data comply with the protocol, GCP, and relevant regulatory requirements. To ensure the reliability and proper handling of data, quality control should be performed at each stage of data processing. In addition, the clinical quality assurance (CQA) department may conduct regular audits of the research process, covering but not limited to research centers, center visits, center laboratories, suppliers, clinical databases, and final clinical research reports. Regulatory authorities may also conduct inspections during the research process or at any time after the study concludes.

During the course of this study, assigned clinical monitors conducted regular on-site monitoring visits to the research center to ensure strict adherence to all study protocol requirements and accurate completion of study data.

1) Participants must possess the professional expertise, qualifications, and capabilities required to undertake this clinical study, and undergo standardized training with uniform documentation methods and evaluation criteria.
2) The entire clinical research process shall be strictly conducted in accordance with this study protocol.
3) Researchers should accurately, thoroughly, and meticulously record all content in the Case Report Form (CRF) in accordance with the CRF completion requirements to ensure the authenticity and reliability of the CRF content.
4) All examinations were conducted using standardized methods and assessment criteria. The abnormality criteria for laboratory tests were determined based on the normal reference ranges established by the respective testing institutions.
5) All observations and findings in clinical studies should be validated to ensure

data reliability and confirm that conclusions are derived from the original data. Appropriate data management measures should be implemented during both the clinical study and data processing phases.

6) Designate monitors to conduct regular on-site inspections of clinical trial sites to ensure strict adherence to the study protocol and accurate, reliable completion of study data.
7) The drug regulatory authority entrusts inspectors not directly involved in the clinical trial to conduct systematic inspections of clinical trial-related activities and documentation, to evaluate whether the trial was conducted in accordance with the protocol, standard operating procedures, and relevant regulatory requirements, and whether trial data were recorded in a timely, truthful, accurate, and complete manner.
8) Researchers should make every effort to minimize subject withdrawals during treatment, keeping the case dropout rate below 20%. For subjects who must withdraw for the benefit of the study, follow-up should still be conducted to facilitate analysis. For withdrawn subjects, all examinations and evaluations required for early study termination should be completed as much as possible.

## XIII. Ethical Requirements and Informed Consent of Subjects

1. Ethics Committee

The research institution or its designated personnel shall prepare the relevant documents to be submitted to the Ethics Committee (EC) of the research center. The trial protocol, informed consent forms, investigator's brochure, subject recruitment materials or advertisements (if applicable), and other documents required by regulations must be submitted to the corresponding EC for approval. Prior to initiating this study, written approval from the EC must be obtained. The EC's approval document must clearly specify the name, number, version number of the trial protocol, and the version number of other documents (e.g., informed consent forms) as well as the approval date.

The research center must comply with the requirements of the Ethics Committee (EC). When revising the protocol, informed consent forms, or subject recruitment materials, the EC must be submitted for approval. In accordance with the safety reporting requirements of the center's location and the EC's regulations, regular reports and updates must be submitted, along with the final version of the report. All the aforementioned documents and EC approvals must be submitted to the research unit or designated personnel.

2. Ethical Implementation of This Study

The research process and the acquisition of informed consent should comply with the Declaration of Helsinki, China GCP requirements, and relevant national laws and regulations to protect the rights, safety, and health of participants. Prior to the initiation of the trial, the protocol must be reviewed by the hospital ethics committee of the responsible research unit, and approval documents must be issued before the trial protocol can be implemented.

3. Subject Information and Informed Consent

Prior to the commencement of the study, the investigator shall use an Informed Consent Form (ICF) to explain the potential risks and benefits of the study to eligible participants, with the language of the informed consent being simple and understandable. The ICF statement shall clearly state that the informed consent is voluntary, that participation in the study entails potential risks and benefits, and that participants may withdraw from the study at any time. The investigator may only enroll a participant after providing a detailed explanation of the study protocol and obtaining written consent from the participant or their legal representative. The ICF shall be signed with a date and retained by the participant as a copy of the informed consent form. Regulations regarding the storage and ownership of clinical research data.

## XIV. Provisions on the Preservation and Ownership of Clinical Research Data

To ensure the evaluation and supervision by the National Medical Products Administration (NMPA), the investigators shall agree to retain all study materials, including original data of all enrolled subjects, all signed informed consent forms, all Case Report Forms (CRFs), and detailed records of drug distribution and recovery. The retention period shall be at least 5 years.

All rights to the clinical study data belong to Tongji Hospital affiliated with Tongji Medical College of Huazhong University of Science and Technology. Except for the National Medical Products Administration (NMPA), the investigators shall not provide the data to any third party in any form without the written consent of the research institution.

## XV. References


1. World Health Organization et al. *World Cancer Report 2014*. Chapter 5.13 (WHO, 2014).
2. Swerdlow, S. H. et al. *WHO Classification of Tumours of Haematopoietic and Lymphoid Tissues*, 4th edn (IARC, 2008).
3. Kenderian, S. S. et al. Chimeric antigen receptor T-cell therapy to target hematologic malignancies. *Cancer Res.* **74**, 6383–6389 (2014).
4. Pico de Coaña, Y. et al. Checkpoint blockade for cancer therapy: revitalizing a suppressed immune system. *Trends Mol. Med.* **21**, 482–491 (2015).
5. Long, A. H. et al. 4-1BB costimulation ameliorates T cell exhaustion induced by tonic signaling of chimeric antigen receptors. *Nat. Med.* **21**, 581–590 (2015).
6. Grupp, S. A. et al. Chimeric antigen receptor–modified T cells for acute lymphoid leukemia. *N. Engl. J. Med.* **368**, 1509–1518 (2013).
7. Kakarla, S. et al. CAR T cells for solid tumors: armed and ready to go? *Cancer J.* **20**, 151–155 (2014).
8. Gilham, D. E. et al. CAR-T cells and solid tumors: tuning T cells to challenge an inveterate foe. *Trends Mol. Med.* **18**, 377–384 (2012).
9. Curran, K. J. et al. Enhancing anti-tumor efficacy of chimeric antigen receptor T cells through constitutive CD40L expression. *Mol. Ther.* **23**, 769–778 (2015).
10. Batlevi, C. L. et al. Novel immunotherapies in lymphoid malignancies. *Nat. Rev. Clin. Oncol.* **13**, 25–40 (2016).
11. Klaver, Y. et al. Adoptive T-cell therapy: a need for standard immune monitoring. *Immunotherapy* **7**, 513–533 (2015).
12. Brentjens, R. J. et al. Safety and persistence of adoptively transferred autologous CD19-targeted T cells in patients with relapsed or chemotherapy-refractory B-cell leukemias. *Blood* **118**, 4817–4828 (2011).
13. Vesely, M. D. et al. Natural innate and adaptive immunity to cancer. *Annu. Rev. Immunol.* **29**, 235–271 (2011).

14. Pule, M. A. et al. Virus-specific T cells engineered to coexpress tumor-specific receptors: persistence and antitumor activity in individuals with neuroblastoma. *Nat. Med.* **14**, 1264–1270 (2008).
15. Dotti, G. et al. Fifteen years of gene therapy based on chimeric antigen receptors: are we nearly there yet? *Hum. Gene Ther.* **20**, 1229–1239 (2009).
16. Park, T. S. et al. Treating cancer with genetically engineered T cells. *Trends Biotechnol.* **29**, 550–557 (2011).
17. Kalos, M. et al. T cells with chimeric antigen receptors have potent antitumor effects and can establish memory in patients with advanced leukemia. *Sci. Transl. Med.* **3**, 95ra73 (2011).
18. Davila, M. L. et al. Efficacy and toxicity management of 19–28z CAR T cell therapy in B cell acute lymphoblastic leukemia. *Sci. Transl. Med.* **6**, 224ra25 (2014).
19. Lee, D. W. et al. How I treat: current concepts in the diagnosis and management of cytokine release syndrome. *Blood* **124**, 188–195 (2014).
20. Maus, M. V. et al. Antibody-modified T cells: CARs take the front seat for hematologic malignancies. *Blood* **123**, 2625–2635 (2014).
