# Supplementary material for: Long-term follow-up demonstrates the curative potential of dual CD19/CD22 CAR-T-cell therapy alone or combined with autologous stem cell transplantation in TP53-altered relapsed/refractory B-cell non-Hodgkin lymphoma
Source: Signal Transduct Target Ther. 2026 Feb 13;11:53. doi: 10.1038/s41392-025-02571-7 (PMC12902020; doi:10.1038/s41392-025-02571-7)
Supplement: Supplementary file 2 — Supplementary Materials [file 41392_2025_2571_MOESM2_ESM.docx]

Supplementary Materials for

Long-term follow-up demonstrates curative potential of dual CD19/CD22 CAR T-cell therapy alone or combined with autologous stem cell transplantation in TP53-altered relapsed/refractory B-Cell non-hodgkin lymphoma

Zekai Mao^1,2#^, Juan Peng^1,2#^, Yang Cao^1,2#^, Na Wang^1,2^, Jue Wang^1,2^，Yang Yang^1,2^, Jinghuan Xu^1,2^, Fankai Meng^1,2^, Liting Chen^1,2,3^, Xia Mao^1,2^, Jiaqi Guo^4^, Xiaoxi Zhou^1,2*^, Yicheng Zhang^1,2*^, Jia Wei^1,2*^

Correspondence to: jiawei@tjh.tjmu.edu.cn; yczhang@tjh.tjmu.edu.cn; cello316@163.com.

**This PDF file includes:**

Materials and Methods

Figures. S1 to S6

Tables S1 to S8

**Materials and Methods**

Study protocols

1. **Cohort A:** An open-label, single-center, single-arm clinical study evaluating sequential infusion of anti-CD22 and anti-CD19 CAR T-cell (CAR19/22 T-cell) in patients with relapsed or refractory (r/r) B-cell malignancies (ChiCTR-OPN-16008526).

**Study Design:**

This is an open-label, prospective, single-arm clinical study conducted at a single center. The study evaluates sequential infusion of anti-CD22 and anti-CD19 autologous CAR T cells in patients with relapsed or refractory B-cell malignancies. All eligible participants receive sequential CAR22 and CAR19 infusions following lymphodepletion chemotherapy. Safety and efficacy will be assessed up to 24 months.

**Objectives:**

Primary Objective:

1. To evaluate the safety and tolerability of sequential infusion of CAR22 and CAR19 T cells in r/r B-cell malignancies.

Secondary Objectives

1. To evaluate preliminary antitumor efficacy.
2. To assess CAR-T cell kinetics and persistence.

**Inclusion criteria:**

Participants must meet all of the following:

1. Signed informed consent obtained before any study procedures.
2. Age ≥ 18 years.
3. Pathologically confirmed CD19⁺/CD22⁺ B-ALL or B-NHL.
4. Relapsed or refractory B-cell malignancy, including: B-ALL; Aggressive B-cell lymphomas: DLBCL, BL, MCL, or transformed B-cell lymphoma.
5. At least one of the following:
   1. Failed ≥2 lines of salvage therapy
   2. Relapsed/refractory post-HSCT.
   3. High-risk features (e.g., double -hit lymphoma)
   4. Ineligible for allo-HSCT (B-ALL) or auto-HSCT (B-NHL)
6. At least one measurable lesion.
7. Adequate organ function, defined as creatinine<2.5mg/dl; aspartate transaminase/ alanine transaminase <3 × upper limit of normal; SiO2 ≥ 95%; bilirubin < 2.0 mg/dl; LVEF > 40%.
8. Sufficient venous access for leukapheresis.
9. ECOG performance status ≤ 2.
10. Estimated survival of ≥ 3 months.

**Exclusion criteria：**

Any of the following will lead to exclusion:

1. Pregnant or lactating women, or planning pregnancy within 1 year
2. Active HBV, HCV, or HIV infection.
3. Uncontrolled systemic infections.
4. Systemic steroid use within 4 weeks.
5. Known allergy to cytokines or antibodies.
6. Participation in other clinical trials within 6 weeks.
7. Active graft-versus-host disease.
8. History of psychiatric disorders or another primary malignancy
9. Substance abuse or addiction.
10. Other conditions deemed unsuitable by the investigator.

**Treatment Regimen：**

For patients enrolled in Cohort A (CAR19/22 T-cell cocktail infusion), lymphodepletion chemotherapy consisting of fludarabine (25 mg/m²/day) and cyclophosphamide (300 mg/m²/day) was administered for 3 consecutive days (days −4 to −2), followed by infusion of CAR19/22 T cells. CAR19 and CAR22 T cells were administered on successive days starting from day 0.

**Endpoints：**

Primary Endpoints

1. Incidence, type, and grade of adverse events (AEs), including CRS and ICANS.
2. 28-day DLT incidence.

Secondary Endpoints

1. ORR, CRR, DOR, PFS, OS (per Lugano criteria or appropriate response standards)
2. CAR transgene copies and expansion kinetics in peripheral blood (peak expansion, AUC, persistence).

**Study Assessments：**

1. Efficacy evaluations: Imaging (CT/PET-CT) and/or bone marrow at baseline, Month 1, Month 3, and every 3 months thereafter.
2. Response assessment criteria: Lugano 2014 / IWCLL / RECIL (as appropriate by disease type).
3. Safety assessments: Continuous AE monitoring, CRS and ICANS grading (ASTCT), laboratory tests, vital signs.
4. CAR-T kinetics monitoring: Peripheral blood qPCR at predefined timepoints during the first 28 days and at follow-up visits.
5. **Cohort B:** An open-label, single center, single arm clinical study for the sequential infusion of anti-CD19 CAR-T and anti-CD22 CAR-T therapy following autologous hematopoietic stem cell transplantation (ASCT) for relapsed, refractory, and high-risk B cell lymphoma (ChiCTR, number ChiCTR-OPN-16009847).

**Study Design：**

This is a prospective, open-label, single-arm clinical study evaluating sequential CAR19 and CAR22 infusion after ASCT consolidation therapy in r/r or high-risk B-cell lymphoma. CAR-T infusions are administered after hematologic recovery from ASCT to improve disease control and prevent relapse.

**Objectives：**

Primary Objective

1. To evaluate the safety and feasibility of sequential CAR19 and CAR22 T-cell infusion following ASCT.

Secondary Objectives

1. To assess preliminary efficacy and survival outcomes.
2. To explore CAR-T cell expansion and persistence after ASCT.

**Inclusion criteria:**

Participants must meet all of the following:

(1) Signed informed consent obtained before any study procedures.

(2) Age ≥ 18 years.

(3) Pathologically confirmed CD19⁺/CD22⁺ B-ALL or B-NHL.

(4) Relapsed or refractory B-cell malignancy, including: B-ALL; Aggressive B-cell lymphomas: DLBCL, BL, MCL, or transformed B-cell lymphoma.

(5) At least one of the following:

a) Failed ≥2 lines of salvage therapy

b) Relapsed/refractory post-HSCT.

c) High-risk features (e.g., double -hit lymphoma)

d) Ineligible for allo-HSCT (B-ALL)

(6) At least one measurable lesion.

(7) Adequate organ function, defined as creatinine<2.5mg/dl; aspartate transaminase/ alanine transaminase <3 × upper limit of normal; SiO2 ≥ 95%; bilirubin < 2.0 mg/dl; LVEF > 40%.

(8) Sufficient venous access for leukapheresis.

(9) ECOG performance status ≤ 2.

(10) Estimated survival of ≥ 3 months.

**Exclusion criteria：**

Any of the following will lead to exclusion:

(1) Pregnant or lactating women, or planning pregnancy within 1 year

(2) Active HBV, HCV, or HIV infection.

(3) Uncontrolled systemic infections.

(4) Systemic steroid use within 4 weeks.

(5) Known allergy to cytokines or antibodies.

(6) Participation in other clinical trials within 6 weeks.

(7) Active graft-versus-host disease.

(8) History of psychiatric disorders or another primary malignancy

(9) Substance abuse or addiction.

(10) Other conditions deemed unsuitable by the investigator.

**Treatment Regimen：**

Chemotherapy conditioning was administered using the BEAM regimen at standard dosage (carmustine 300 mg/m² on day −6; etoposide 200 mg/m² on days −5 to −2; cytarabine 400 mg/m² on days −5 to −2; and melphalan 140 mg/m² on day −1), followed by autologous stem cell infusion on day 0. Subsequently, CAR19/22 T-cell cocktail infusion was performed within 2–6 days after ASCT (days +2 to +6), according to clinical judgment and product availability.

**Endpoints：**

Primary Endpoints

1. Incidence, type, and grade of adverse events (AEs), including CRS and ICANS.
2. 28-day DLT incidence.

Secondary Endpoints

1. ORR, CRR, DOR, PFS, OS (per Lugano criteria or appropriate response standards)
2. CAR transgene copies and expansion kinetics in peripheral blood (peak expansion, AUC, persistence).

**Study Assessments：**

1. Efficacy evaluations: Imaging (CT/PET-CT) and/or bone marrow at baseline, Month 1, Month 3, and every 3 months thereafter.
2. Response assessment criteria: Lugano 2014 / IWCLL / RECIL (as appropriate by disease type).
3. Safety assessments: Continuous AE monitoring, CRS and ICANS grading (ASTCT), laboratory tests, vital signs.
4. CAR-T kinetics monitoring: Peripheral blood qPCR at predefined timepoints during the first 28 days and at follow-up visits.

Sequencing and mutational analysis

TP53 gene alterations, including mutations and/or deletion of the 17p chromosomal region [del(17p)], were identified in patients with relapsed or refractory B-cell non-Hodgkin lymphoma (r/r B-NHL) using next-generation sequencing (NGS) or fluorescence in situ hybridization (FISH) ^1^. Targeted sequencing of TP53 was conducted using custom-designed panels, with libraries sequenced on the Illumina NextSeq 550 platform. Variant calling and annotation were performed using the BaseSpace Sequence Hub and ANNOVAR software, with filtering restricted to exonic, nonsynonymous, or splice-site variants occurring at a population frequency of less than 0.001 in the gnomAD database. For cytogenetic assessment, interphase FISH was performed on formalin-fixed, paraffin-embedded (FFPE) tissues or fixed cell preparations using a commercially available 17p13.1 probe (Vysis, Downers Grove, IL, USA) to detect del(17p).

As described previously, four functional classification systems were applied. First, mutations were categorized as missense or non-missense, the latter including nonsense mutations, splice-site mutations, and small insertions or deletions^2^. Second, mutations were stratified as disruptive or non-disruptive, based on their predicted structural and functional impact on the p53 protein, as previously described in studies of acute myeloid leukemia (AML) and solid tumors^3^. Third, the evolutionary action score (EAp53) was utilized to further characterize missense mutations, with scores ranging from 0 (wild-type) to 100, reflecting the evolutionary sensitivity of affected residues; an EAp53 threshold of 75 was used to distinguish between high-risk (≥75) and low-risk (<75) variants^4^. The EAp53 scores were obtained from the German-Austrian AML Study Group database (http://mammoth.bcm.tmc.edu/EAp53). Lastly, patients were classified according to the presence or absence of del(17p). These classification approaches allowed a comprehensive evaluation of TP53 alterations and supported analysis of whether CAR T-cell therapy could offset their adverse prognostic impact.

**Supplemental figures**

Figure. S1


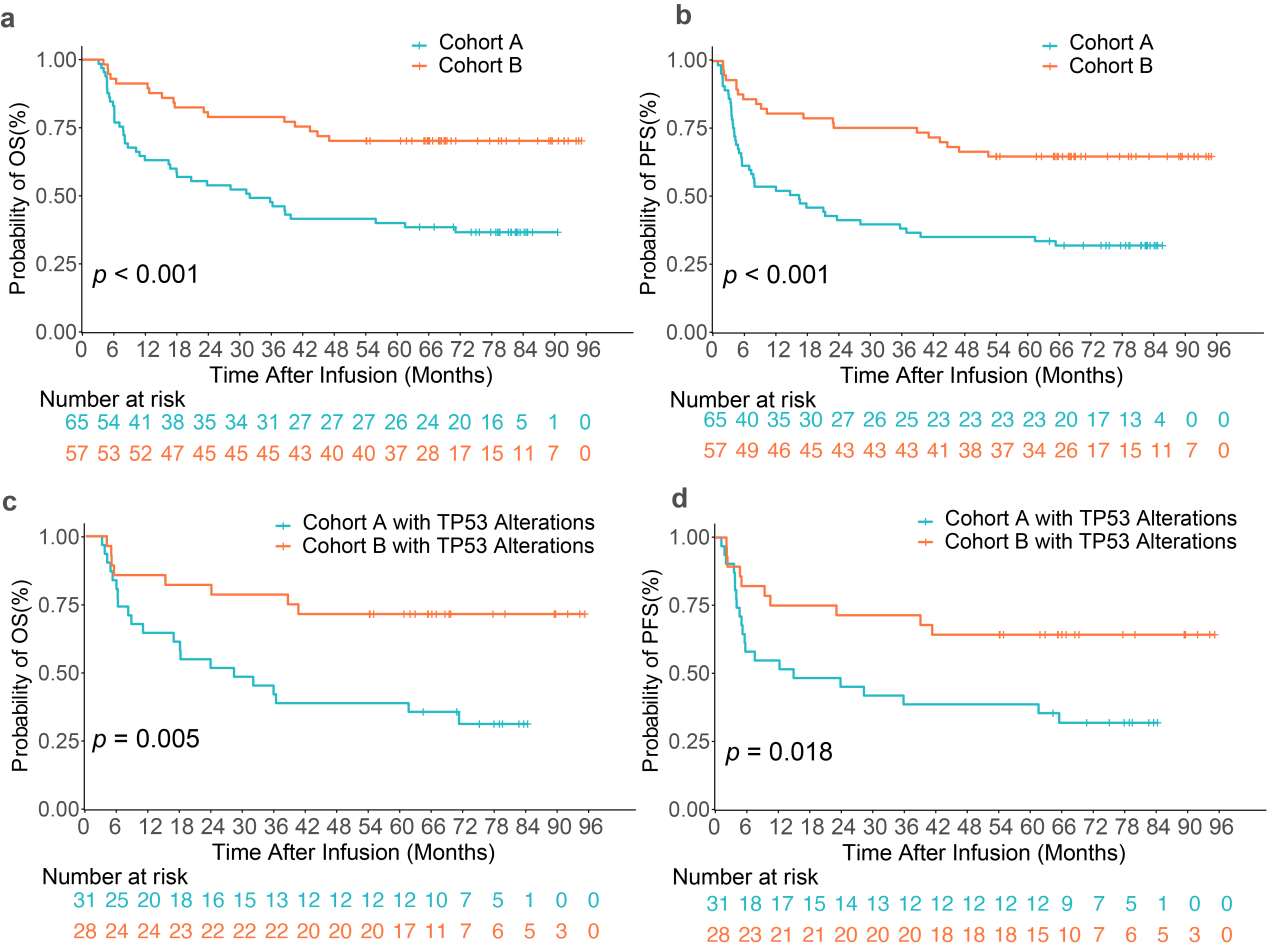


**Figure S1:** Kaplan–Meier survival curves comparing overall survival (OS) and progression-free survival (PFS) between two patient cohorts. (**a**) OS in Cohort A vs Cohort B. (**b**) PFS in Cohort A vs Cohort B. (**c**) OS in patients with TP53 alterations stratified by cohort. (**d**) PFS in patients with TP53 alterations stratified by cohort.

Figure. S2.


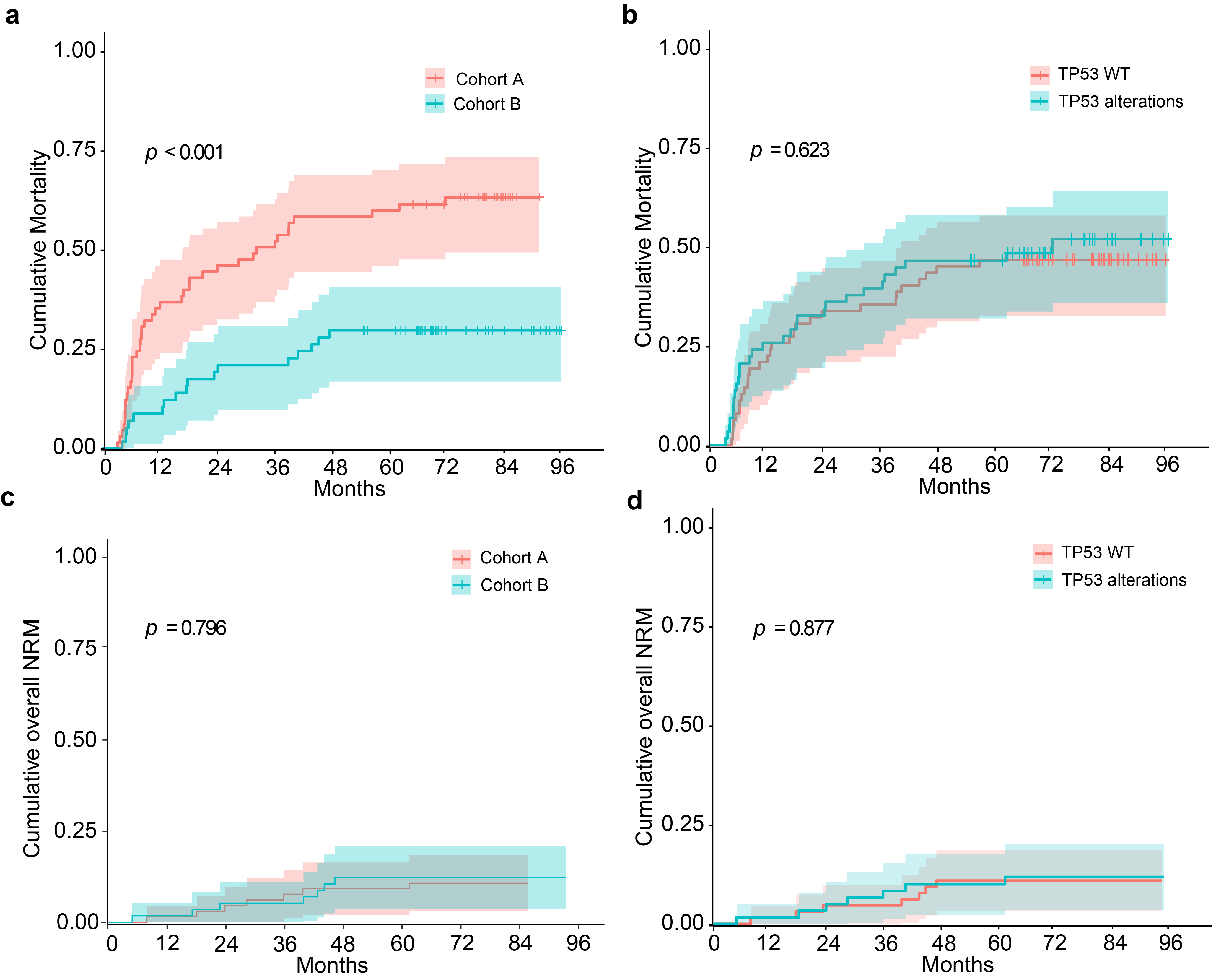


**Figure. S2.** (**a**) Cumulative overall mortality between cohort A and B. (**b**) Cumulative overall mortality stratified by TP53 mutation status. (**c**) Cumulative incidence of non-relapse mortality (NRM) between cohort A and B. (**d**) Cumulative incidence of NRM stratified by TP53 mutation status. Shaded areas represent 95% confidence intervals (CIs). P values were calculated using the log-rank test for overall mortality (**a**–**b**) and Gray’s test for NRM (**c**–**d**).

Figure. S3.


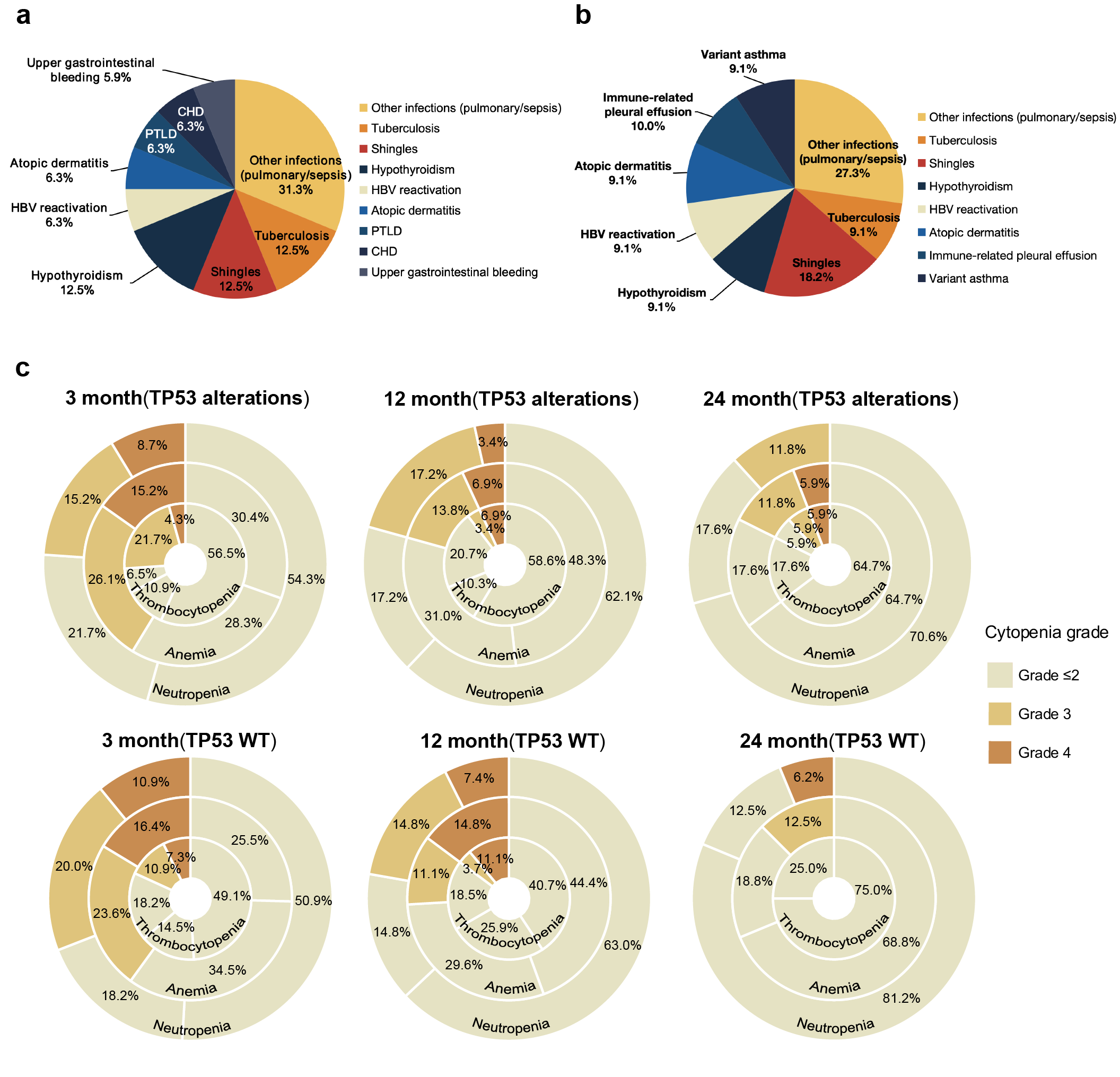


**Figure. S3.** Pie charts displaying the distribution of SAEs stratified by TP53 alteration status: TP53-altered (**a**) and TP53-wildtype (**b**). (**c**) Circular charts showing hematologic toxicity grades at 3, 12, and 24 months after infusion according to TP53 alteration status..

Figure. S4.

**
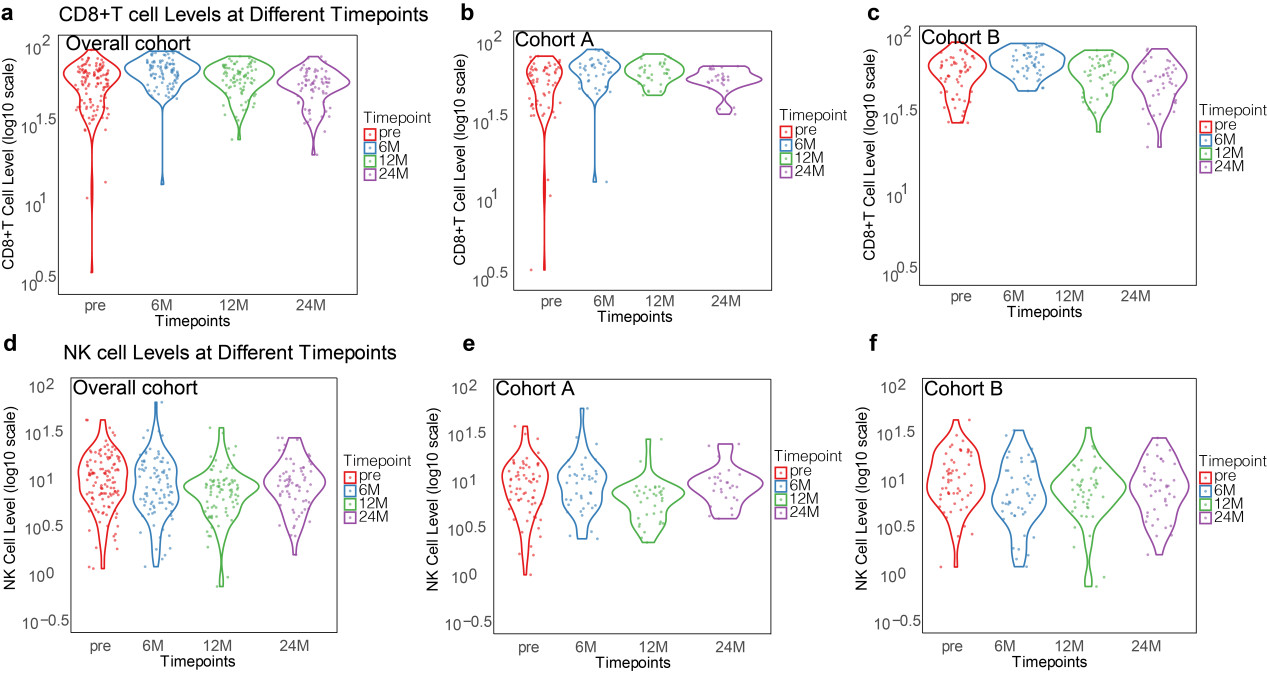
**

**Figure. S4.** Violin plots displaying the levels of various immune cell types (CD8+ T cells, NK cells) at different timepoints (pre-infusion, 6 months post-infusion, 12 months post-infusion, and 24 months post-infusion) across the overall population and in two cohorts (A and B).

Figure. S5.

**
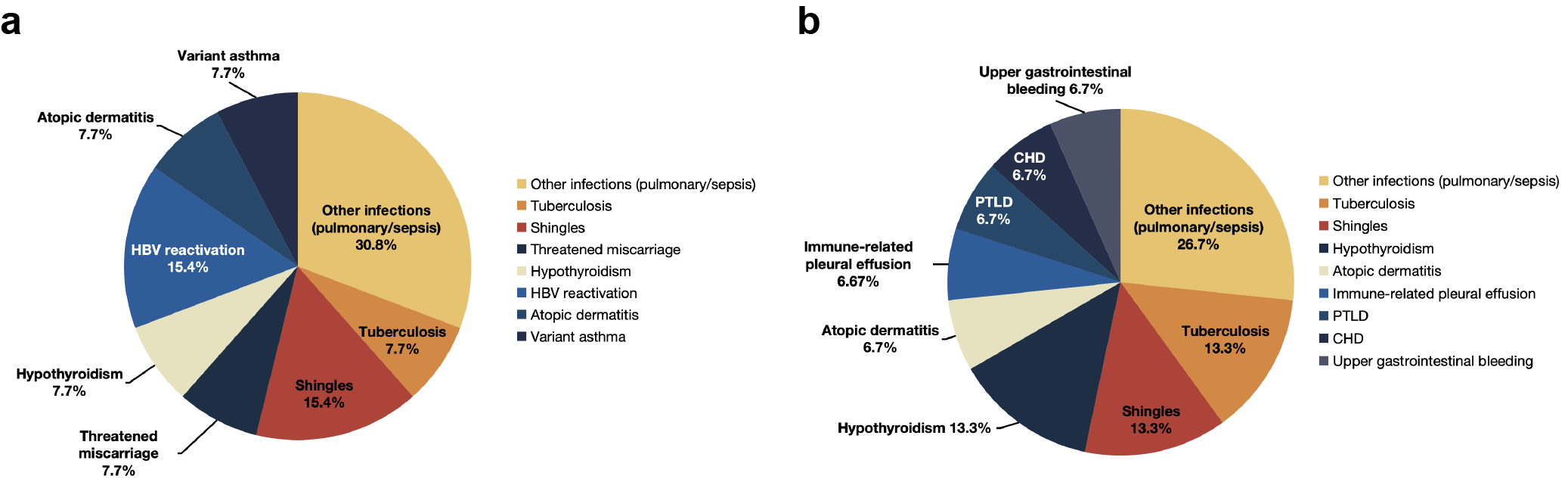
**

**Figure. S5.** Pie charts displaying the distribution of Serious Adverse Events (SAEs); (**a**–**b**) Distribution of SAEs in Cohort A (**a**) and Cohort B (**b**).

Figure. S6.

**
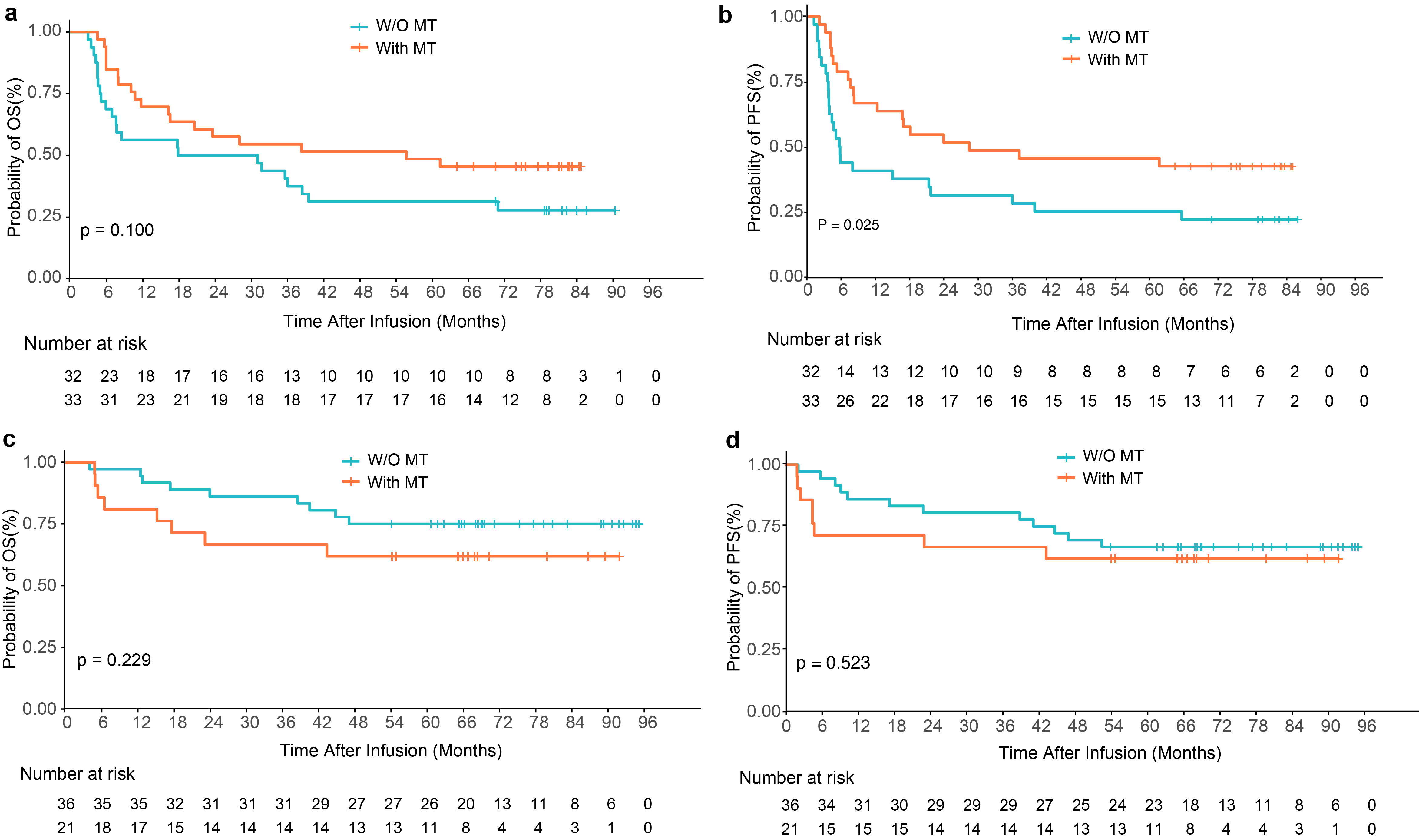
**

**Figure S6.** Kaplan–Meier curves comparing overall survival (OS) and progression-free survival (PFS) with or without (W/O) maintenance therapy (MT) in patients from Cohort A and Cohort B. (**a**) OS in Cohort A: patients with MT vs. without MT. (**b**) PFS in Cohort A: patients with MT vs. without MT. (c) OS in Cohort B: patients with MT vs. without MT. (**d**) PFS in Cohort B: patients with MT vs. without MT.

**Supplemental tables**

Table S1. Patients’ characteristics.

| Characteristics | Values | | | |
| --- | --- | --- | --- | --- |
|  | TP53 WT in Cohort A | TP53 altered in Cohort A | TP53 WT in Cohort B | TP53 altered in Cohort B |
| Patients number | 34 | 31 | 29 | 28 |
| Age: ≥60 | 10(29.4%) | 2(6.5%) | 1(3.4%) | 4(14.3%) |
| Gender(M/F) | 21/13 | 21/10 | 16/13 | 18/10 |
| ECOG PS |  |  |  |  |
| 0-1 | 20(58.8%) | 21(67.7%) | 22(75.9%) | 16(57.1%) |
| 2 | 14 (41.2%) | 10(31.3%) | 7(24.2%) | 12(42.8%) |
| Pathologic subtype |  |  | |  |
| DLBCL,NOS | 20(58.8%) | 25(80.6%) | 22(75.9%) | 18(64.3%) |
| tFL-DLBCL | 6(17.6%) | 3(9.4%) | 4(13.8%) | 4(14.3%) |
| HGBL DHL | 5(14.8%) | 0(0.0%) | 3(10.3%) | 2(7.1%) |
| HGBL,NOS | 2(5.8%) | 1(3.1%) | 0(0.0%) | 0(0.0%) |
| Burkitt lymphoma | 1(2.9%) | 1(3.1%) | 0(0.0%) | 2(7.1%) |
| MCL | 0(0.0%) | 1(3.1%) | 0(0.0%) | 0(0.0%) |
| others^a^ | 0(0.0%) | 0(0.0%) | 0(0.0%) | 2(7.1%) |
| Bulky disease |  |  |  |  |
| yes | 13(38.2%) | 16(51.6%) | 8(27.6%) | 7(25.0%) |
| Disease status |  |  |  |  |
| Primary refractory | 6(17.6%) | 11(35.5%) | 12(41.4%) | 8(28.6%) |
| First relapse | 16(47.0%) | 6(18.8%) | 10(14.5%) | 10(35.7%) |
| ≥2nd relapse | 12(35.3%) | 14(45.1%) | 7(24.1%) | 10(35.7%) |
| No.of treatment lines |  |  |  |  |
| 2 | 10(29.4%) | 3(9.4%) | 4(13.8%) | 8(28.6%) |
| ≥3 | 24(24.7%) | 28(28.9%) | 25(25.8%) | 20(20.6%) |
| IPI score |  |  |  |  |
| 0-2 | 15(44.1%) | 13(40.6%) | 14(48.3%) | 10(35.7%) |
| 3-5 | 19(55.9%) | 18(58.1%) | 15(51.7%) | 18(64.3%) |

^a^Others, including 1 with B-cell lymphoma, unclassified with properties between DLBCL and classical Hodgkin lymphoma, and 1 with B-cell lymphoblastic lymphoma; ^b^Disease stage based on the modified Ann Arbor staging system. *WT* Wild Type, *ECOG PS* Eastern Cooperative Oncology Group (ECOG) performance status, *DLBCL NOS* diffuse large B-cell lymphoma, not otherwise specified, *tFL-DLBCL* diffuse large B-cell lymphoma transformed from follicular lymphoma (FL), *HGBL DHL* high-grade B-cell lymphoma with MYC and BCL2 and/or BCL6 rearrangements, *HGBL NOS* HGBL, not otherwise specified, *MCL* mantle cell lymphoma, *ASCT* autologous stem cell transplantation; *LDH* lactate dehydrogenase, ULN upper limit of normal, *PR* partial response, *SD* stable disease, *PD* progressive disease, *IPI* international prognostic index, *GCB* germinal center B-cell like.

Table S2: Maintenance Treatment Strategies Among Patients in Cohort A and B.

| Maintenance therapy | Cohort A  (n=65) | Cohort B  (n=57) |
| --- | --- | --- |
| PD-1 | 28(43.1%) | 14(24.6%) |
| Radiotherapy | 1(1.5%) | 0(0.0%) |
| PD-1 inhibitor + Chidamide | 1(1.5%) | 4(7.0%) |
| PD-1 inhibitor + Radiotherapy | 0(0.0%) | 1(1.8%) |
| PD-1 inhibitor + Chidamide + Radiotherapy | 1(1.5%) | 0(0.0%) |

Table S3. Characteristics of Secondary Malignancies (SMNs) After CAR-T Cell Infusion.

| Patient | Diagnosis | Lymphoma Progression | Time to SMN Diagnosis From lymphoma Diagnosis, months | Time to SMN Diagnosis From CAR-T Infusion, months | cohort | TP53 mutated | TP53 deletion | Number of lines of therapy for lymphoma | Therapy for SMN | OS From SMN Diagnosis(months) | Vital Status |
| --- | --- | --- | --- | --- | --- | --- | --- | --- | --- | --- | --- |
| 1 | Gastrointestinal tumors | No progress | 108 | 26 | B | yes | yes | 4 | Targeted therapy | 11 | Died |
| 2 | AML-M5 | No progress | 54 | 46 | B | yes | yes | 2 | Chemotherapy | 48 | Alive |
| 3 | Lung cancer | No progress | 26 | 14 | A | no | yes | 4 | Chemotherapy and radiotherapy | 40 | Died |

Table S4. Incidence of serious long-term non-relapse complications by treatment cohort.

| Complication | Cohort A, n (%) | Cohort B, n (%) |
| --- | --- | --- |
| Pulmonary infection or sepsis | 4(6.2%) | 4(7.0%) |
| Tuberculosis^a^ | 1(1.5%) | 2(3.5%) |
| Herpes zoster | 2(3.1%) | 2(3.5%) |
| Hypothyroidism | 1(1.5%) | 2(3.5%) |
| Hepatitis B reactivation^b^ | 2(3.1%) | 0(0.0%) |
| Atopic dermatitis | 1(1.5%) | 1(1.8%) |
| Immunotherapy-induced pleural effusion | 0(0.0%) | 1(1.8%) |
| Variant asthma | 1(1.5%) | 0(0.0%) |
| Post-transplant lymphoproliferative disorder | 0(0.0%) | 1(1.8%) |
| Coronary artery disease | 0(0.0%) | 1(1.8%) |
| Upper gastrointestinal bleeding | 0(0.0%) | 1(1.8%) |

^a^: Prophylaxis for tuberculosis was not implemented considering both the relatively low incidence and potential adverse effects of preventive drugs, following consensus guidelines and weighing risks and benefits for patients. ^b^: For hepatitis B, viral reactivation occurred despite entecavir prophylaxis, which was subsequently controlled by combined entecavir and tenofovir therapy.

Table S5. Incidence of serious long-term non-relapse complications by TP53 alteration status.

| Complication | TP53 altered, n (%) | TP53 WT, n (%) |
| --- | --- | --- |
| Pulmonary infection or sepsis | 5(8.5%) | 3(4.8%) |
| Tuberculosis^a^ | 2(3.4%) | 1(1.6%) |
| Herpes zoster | 2(3.4%) | 2(3.2%) |
| Hypothyroidism | 2(3.4%) | 1(1.6%) |
| Hepatitis B reactivation^b^ | 1(1.7 %) | 1(1.6%) |
| Atopic dermatitis | 1(1.7 %) | 1(1.6%) |
| Immunotherapy-induced pleural effusion | 0(0.0%) | 1(1.6%) |
| Variant asthma | 0(0.0%) | 1(1.6%) |
| Post-transplant lymphoproliferative disorder | 1(1.7 %) | 0(0.0%) |
| Coronary artery disease | 1(1.7 %) | 0(0.0%) |
| Upper gastrointestinal bleeding | 1(1.7 %) | 0(0.0%) |

^a^: Prophylaxis for tuberculosis was not implemented considering both the relatively low incidence and potential adverse effects of preventive drugs, following consensus guidelines and weighing risks and benefits for patients. ^b^: For hepatitis B, viral reactivation occurred despite entecavir prophylaxis, which was subsequently controlled by combined entecavir and tenofovir therapy.

Table S6. Univariate and multivariate analysis of prognostic factors for OS and PFS in cohort A.

| Variable | OS Univariate | | OS Multivariate | | PFS Univariate | | PFS Multivariate | |
| --- | --- | --- | --- | --- | --- | --- | --- | --- |
|  | HR (95% CI) | *p* | HR (95% CI) | *p* | HR (95% CI) | *p* | HR (95% CI) | *p* |
| Sex: Male vs. Female | 0.91 (0.48 - 1.73) | 0.781 |  |  | 0.79 (0.43 - 1.44) | 0.435 |  |  |
| Age: <60 vs. ≥60 | 1.22 (0.58 - 2.56) | 0.594 |  |  | 1.47 (0.72 - 2.97) | 0.289 |  |  |
| ECOG PS: 0–1 vs. 2 | 1.35 (0.72 - 2.53) | 0.350 | 1.38 (0.73 - 2.62) | 0.317 | 1.28 (0.70 - 2.35) | 0.426 | 1.35 (0.73 - 2.50) | 0.346 |
| Bulky disease: Present vs. Absent | 1.11 (0.60 - 2.06) | 0.735 | 1.17 (0.63 - 2.18) | 0.620 | 1.25 (0.69 - 2.26) | 0.460 | 1.32 (0.72 - 2.41) | 0.368 |
| TP53 altered: Present vs. Absent | 1.23 (0.67 - 2.27) | 0.507 |  |  | 0.99 (0.55 - 1.78) | 0.961 |  |  |
| Double expression: Present vs. Absent | 1.71 (0.90 - 3.27) | 0.103 |  |  | 1.58 (0.85 - 2.93) | 0.149 |  |  |
| CNS involvement: Present vs. Absent | 1.33 (0.59 - 3.01) | 0.488 |  |  | 1.12 (0.50 - 2.51) | 0.784 |  |  |
| Burkitt lymphoma: Yes vs. No | 2.20 (0.53 - 9.18) | 0.278 |  |  | 2.55 (0.61 - 10.64) | 0.199 |  |  |
| Double-Hit: Present vs. Absent | 1.22 (0.56 - 2.67) | 0.619 |  |  | 1.06 (0.49 - 2.30) | 0.878 |  |  |
| TP53 missense mutation groups: Non-missense vs. Missense | 1.19 (0.43 - 3.30) | 0.741 |  |  | 1.19 (0.43 - 3.32) | 0.735 |  |  |
| TP53 disruptive mutation groups: Non-disruptive vs. Disruptive | 1.33 (0.53 - 3.34) | 0.546 |  |  | 1.35 (0.54 - 3.36) | 0.526 |  |  |
| EAp53 score: High risk vs. Low risk | 0.86 (0.30 - 2.50) | 0.780 |  |  | 0.97 (0.33 - 2.79) | 0.948 |  |  |
| Del(17p): Yes vs. No | 0.76 (0.27 - 2.17) | 0.608 |  |  | 0.79 (0.28 - 2.24) | 0.656 |  |  |
| Treatment line number: 2 lines vs. ≥3 lines | 1.49 (0.63 - 3.55) | 0.367 |  |  | 1.27 (0.57 - 2.86) | 0.558 |  |  |

OS, Overall Survival; PFS: Progression-Free Survival; HR, Hazard Ratio; CI, Confidence Interval; ECOG PS, Eastern Cooperative Oncology Group Performance Status; TP53 altered, any TP53 mutation or deletion; Double expression, concurrent MYC and BCL2 overexpression; CNS, Central Nervous System; Double-Hit, lymphoma with MYC and BCL2/BCL6 rearrangements; Del(17p), deletion of chromosome 17p; EAp53 score, Evolutionary Action score of TP53.

Table S7. Univariate and Multivariate analysis of prognostic factors for OS and PSF in cohort B.

| Variable | OS Univariate | | OS Multivariate | | PFS Univariate | | PFS Multivariate | |
| --- | --- | --- | --- | --- | --- | --- | --- | --- |
|  | HR (95% CI) | *p* | HR (95% CI) | *p* | HR (95% CI) | *p* | HR (95% CI) | *p* |
| Sex: Male vs. Female | 2.48 (0.81 - 7.62) | 0.112 |  |  | 2.37 (0.86 - 6.51) | 0.095 | 8.15 (0.52 - 126.83) | 0.134 |
| Age: <60 vs. ≥60 | 1.36 (0.31 - 5.94) | 0.685 |  |  | 1.21 (0.28 - 5.21) | 0.800 |  |  |
| ECOG PS: 0–1 vs. 2 | 2.54 (0.98 - 6.59) | 0.056 | 1.48 (0.54 - 4.11) | 0.447 | 2.34 (0.97 - 5.65) | 0.059 | 1.68 (0.06 - 50.88) | 0.765 |
| Bulky disease: Present vs. Absent | 6.02 (2.26 - 15.99) | <0.001 | 5.25 (1.86 - 14.86) | 0.002 | 4.49 (1.85 - 10.87) | <0.001 | 1.88 (0.16 - 22.25) | 0.618 |
| TP53 altered: Present vs. Absent | 0.98 (0.38 - 2.53) | 0.959 |  |  | 1.15 (0.48 - 2.75) | 0.760 |  |  |
| Double expression: Present vs. Absent | 0.76 (0.28 - 2.10) | 0.598 |  |  | 0.74 (0.29 - 1.88) | 0.525 |  |  |
| CNS involvement: Present vs. Absent | 0.00 (0.000 - Inf) | 0.998 |  |  | 0.00 (0.000 - Inf) | 0.998 |  |  |
| Burkitt lymphoma: Yes vs. No | 0.00 (0.000 - Inf) | 0.998 |  |  | 0.00 (0.000 - Inf) | 0.998 |  |  |
| Double-Hit: Present vs. Absent | 0.78 (0.17 - 3.63) | 0.753 |  |  | 0.63 (0.14 - 2.84) | 0.545 |  |  |
| TP53 missense mutation groups: Non-missense vs. Missense | 0.66 (0.12 - 3.65) | 0.638 |  |  | 1.08 (0.22 - 5.36) | 0.925 |  |  |
| TP53 disruptive mutation groups: Non-disruptive vs. Disruptive | 2.14 (0.43 - 10.64) | 0.354 |  |  | 1.14 (0.27 - 4.79) | 0.855 |  |  |
| EAp53 score: High risk vs. Low risk | 0.33 (0.03 - 3.18) | 0.337 |  |  | 0.15 (0.02 - 1.33) | 0.090 | 0.14 (0.01 - 1.29) | 0.082 |
| Del(17p): Yes vs. No | 1.65 (0.19 - 14.13) | 0.648 |  |  | 0.53 (0.13 - 2.22) | 0.385 |  |  |
| Treatment line number: 2 lines vs. ≥3 lines | 0.67 (0.22 - 2.04) | 0.476 |  |  | 0.87 (0.29 - 2.61) | 0.807 |  |  |

Table S8. Univariate and Multivariate Landmark Cox analysis of prognostic factors for OS and PFS at 3 months in the overall population.

| Variable | OS Univariate | | OS Multivariate | | PFS Univariate | | PFS Multivariate | |
| --- | --- | --- | --- | --- | --- | --- | --- | --- |
|  | HR (95% CI) | *p* | HR (95% CI) | *p* | HR (95% CI) | *p* | HR (95% CI) | *p* |
| Sex: Male vs. Female | 1.15 (0.64 - 2.09) | 0.634 |  |  | 1.15 (0.66 - 2.02) | 0.627 |  |  |
| Age: <60 vs. ≥60 | 1.73 (0.86 - 3.48) | 0.123 |  |  | 1.97 (1.01 - 3.83) | 0.046 | 1.67 (0.85 - 3.28) | 0.138 |
| Treatment option: Cohort A vs. Cohort B | 3.37 (1.78 - 6.38) | <0.001 | 2.34 (1.10 - 4.95) | 0.027 | 3.05 (1.69 - 5.49) | <0.001 | 2.07 (1.01 - 4.23) | 0.046 |
| ECOG PS: 0–1 vs. 2 | 1.90 (1.08 - 3.36) | 0.026 | 1.35 (0.70 - 2.63) | 0.369 | 1.70 (0.99 - 2.92) | 0.053 |  |  |
| Bulky disease: Present vs. Absent | 2.04 (1.15 - 3.6) | 0.014 | 1.83 (0.99 - 3.38) | 0.052 | 1.93 (1.12 - 3.33) | 0.018 | 1.51 (0.86 - 2.66) | 0.156 |
| NRR3: Response vs. No response | 39.30 (11.30 - 136.69) | <0.001 | 29.82 (8.08 - 110.14) | <0.001 | 27.46 (8.04 - 93.75) | <0.001 | 18.39 (5.32 - 63.61) | <0.001 |
| CRS: 0–1 vs. 2 | 2.18 (1.24 - 3.84) | 0.007 | 1.02 (0.50 - 2.11) | 0.954 | 2.36 (1.37 - 4.05) | 0.002 | 1.25 (0.65 - 2.38) | 0.503 |
| ICANS: Present vs. Absent | 1.24 (0.60 - 2.57) | 0.554 |  |  | 1.06 (0.52 - 2.17) | 0.871 |  |  |
| TP53 altered: Present vs. Absent | 1.02 (0.58 - 1.79) | 0.956 |  |  | 0.99 (0.58 - 1.70) | 0.976 |  |  |
| Double expression: Present vs. Absent | 1.27 (0.71 - 2.28) | 0.423 |  |  | 1.10 (0.63 - 1.92) | 0.740 |  |  |
| CNS involvement: Present vs. Absent | 1.84 (0.83 - 4.12) | 0.135 |  |  | 1.59 (0.72 - 3.52) | 0.256 |  |  |
| Burkitt lymphoma: Yes vs. No | 0.63 (0.09 - 4.56) | 0.647 |  |  | 0.59 (0.08 - 4.25) | 0.598 |  |  |
| Double-Hit: Present vs. Absent | 1.42 (0.68 - 2.95) | 0.345 |  |  | 1.26 (0.62 - 2.59) | 0.524 |  |  |
| TP53 missense mutation groups: Non-missense vs. Missense | 0.70 (0.28 - 1.76) | 0.453 |  |  | 0.86 (0.35 - 2.11) | 0.741 |  |  |
| TP53 disruptive mutation groups: Non-disruptive vs. Disruptive | 2.23 (0.91 - 5.47) | 0.081 |  |  | 1.68 (0.72 - 3.9) | 0.227 |  |  |
| EAp53 score: High risk vs. Low risk | 0.62 (0.20 - 1.90) | 0.404 |  |  | 0.49 (0.17 - 1.38) | 0.176 |  |  |
| Del(17p): Yes vs. No | 1.53 (0.44 - 5.32) | 0.506 |  |  | 0.86 (0.31 - 2.39) | 0.773 |  |  |
| Maintenance therapy: Present vs. Absent | 1.07 (0.61 - 1.90) | 0.803 |  |  | 0.93 (0.54 - 1.61) | 0.804 |  |  |

CRS, Cytokine Release Syndrome; ICANS, Immune effector Cell-Associated Neurotoxicity Syndrome; NRR3, not reached remission at 3 months.

**References**

1. Cao, Y. et al. Mutations or copy number losses of CD58 and TP53 genes in diffuse large B-cell lymphoma are independent unfavorable prognostic factors. ***Oncotarget*** **7**, 83294–83307 (2016).
2. Loizou, E. et al. A gain-of-function p53-mutant oncogene promotes cell fate plasticity and myeloid leukemia through the pluripotency factor FOXH1. ***Cancer Discov.*** **9**, 962–979 (2019).
3. Ward, M. C. et al. Cost-effectiveness analysis of endocrine therapy alone versus partial-breast irradiation alone versus combined treatment for low-risk hormone-positive early-stage breast cancer in women aged 70 years or older. ***Breast Cancer Res. Treat.*** **182**, 355–365 (2020).
4. Neskey, D. M. et al. Evolutionary action score of TP53 identifies high-risk mutations associated with decreased survival and increased distant metastases in head and neck cancer. ***Cancer Res.*** **75**, 1527–1536 (2015).
